# Proceedings of the International Symposium: Innovation for the Management of Echinococcosis Besançon, March 27–29, 2014

**DOI:** 10.1051/parasite/2014024

**Published:** 2014-06-25

**Authors:** Dominique A. Vuitton, Laurence Millon, Bruno Gottstein, Patrick Giraudoux

## Session 1. New tools for patient diagnosis and follow-up

### 1A. Imaging tools for diagnosis and follow-up

#### State-of-the art

##### L-01. Innovation in echinococcosis imaging: new tools or better use of old ones?

Liu Wenya

Imaging Center, First Affiliated Hospital, Xinjiang Medical University, Urumqi, China

13999202977@163.com

Hepatic Alveolar Echinococcosis (HAE) is a rare but life-threatening parasitic disease which has the feature of infiltrating growth like tumor. Because HAE is a chronic disease with a latent stage that may last years before signs and symptoms develo, the diagnosis primarily depends on imaging techniques including ultrasonography (US), Computed Tomography (CT), Magnetic Resonance Imaging (MRI) and Positron Emission Tomography (PET)-CT. It is necessary to look into those imaging development to establish an optimal strategy for HAE diagnosis.

US is the first choice due to its widespread availability, radiation-free and low costs. It has limited contribution for small peripheral lesions, and is unsatisfactory in the evaluation of extension into the adjacent periparasitic liver. So, CT/MRI frequently follows for further evaluation. Though radiation is the drawback of CT, it remains the mainstream modality for morphologic imaging assessment of HAE lesions in most of developing countries or districts. CT has a clear superiority over MR and US, particularly in demonstrating calcification, especially in small clusters. It also helps to stage the disease and provide comprehensive information about vascular, biliary, and extrahepatic extension. With high resolution for soft tissue and without any radiation, MRI is the best modality for characterizing the parasitic lesions and depicting vascular or biliary tree involvement and extra-hepatic extension. PET-CT is a noninvasive tool for detection of metabolic activity in HAE by evaluating the glucose metabolism of hepatic lesions. It provides valuable information in surveillance of the efficacy of chemotherapy. Its value in detecting metastases has still to be evaluated.

According to the clinical tasks, there are three steps in the imaging approach to HAE: firstly diagnosis of HAE which is usually done by routine imaging; secondly assessment of the PNM staging of HAE before operation or chemotherapy which mainly relies on those new techniques of CT/MRI such as MR Perfusion (MRP), CT Angiography (CTA)/MR Angiography (MRA), CT Cholangiography (CTC)/MR Cholangiography; and finally surveillance of the treatment: PET-CT is currently considered the best tool for evaluating the effect of treatment. Yet it is costly and not readily available, so it has a limited availability, especially in those developing countries and districts. Fortunately, new techniques such as contrast enhanced US (CEUS), CT Perfusion (CTP), spectral CT, and MRI-Diffusion Weighted Imaging (DWI) have received attention recently, and could take place of PET-CT in some degree with their advantage of low-cost and easy reach.

Micro-bubble contrast agents have been developed to improve US imaging and the technique of CEUS was proposed for the diagnosis and evaluation of HAE lesions in both human beings and in the rat model (Zeng Hongchun et al., 2012). It was used to identify “disease activity” and for the follow-up of imaging changes. With the development of the HAE, advanced lesions present with the typical “ring enhancement” in arterial phase and no enhancement in the centre of lesions in arterial and portal vein phase (). CT perfusion provides an interesting functional imaging for detecting the micro-circulation of HAE. It shows different levels of blood perfusion on the margin, center of HAE and nearby hepatic perenchyma. There was good correlation between blood flow, blood volume and microvessel density (MVD) in the same region of HAE (Wang Jing et al., 2011). Energy spectral CT, with much lower radiation, demonstrates the same changes in blood supply of HAE by measuring iodine concentration instead of CT perfusion. In addition, comparison between spectral CT and PET-CT has found that the enhancement and images of well-perfused region of AE in spectral CT were consistent with PET-CT finding. This result indicates that spectral CT imaging based on the spectral differentiation of iodine is technically feasible and can quantitatively identify the micro-perfusion status and indirectly reflect the activity of AE (Jiang Yi et al., ImE-2014). Studies about the utility of MRI-DWI in detection and characterization of HAE have achieved optimized result. Firstly, the initial study revealed that the value of DWI both in detection and characterization of the HAE as well as distinguishing the peri-lesional intense fibrogenesis zone which may represent a protective response of the host (Ren Bo et al., 2012). It appears also to reflect the activity of the parasite in some degree. DWI showed a clear advantage over conventional MR protocols, especially in the small lesions less than 1 cm. It may be used in experimental animals to detect early lesions (Zeng Hongchun et al., ImE-2014). Secondly, a correlated study between DWI parameters (apparent diffusion coefficient – ADC-values) and two pathologic markers including micro-vessel density (MVD) and percentage of fibrosis area (using MASSON staining) was performed in 27 cases (Xie Weidong et al., 2012), respectively. Statistical analysis showed that there were significant differences among ADC values in different part of HAE lesion. Further, there was a significantly inverse correlation between the ADC values and the percentage of fibrosis area in the peripheral area of HAE (: = −0.767, *p* = 0.001). Finally, the potential “viability zone” of HAE in DWI was estimated and compared with that of PET/CT. Concordance was tested in 7 cases who underwent PET/CT at same period of time. The peri-lesional hyper-intense zone of the HAE lesion in DWI imaging was just similar to the distribution of the “hot spot” in PET/CT imaging (Wang Jing et al., ImE-2014).

In Summary, the first diagnosis of HAE is generally made by US owing to its abdominal symptoms or because of general check-up. The next step is frequently CT or MR examination for further characterization of the lesion. PET-CT used to be the only noninvasive tool for detection of metabolic activity in HAE by evaluating the glucose metabolism of hepatic lesions, but of limited use because of its availability and cost. More easily available functional techniques of CT or MRI were explored in the recent years, and compared with PET/CT results. The initial study showed that new techniques such as CE-US, CTP and MRI-DWI can detect the blood supply and metabolism of HAE, and in some degree, with further evaluation, these new techniques could become an alternative to PET-CT.

**References**

Zeng HC, Wang J, Xie W, Liu W, Wen H. Assessment of early hepatic *Echinococcus multilocularis* infection in rats with real-time contrast-enhanced ultrasonography. Ultrasound Med Biol. 2012;38 (11):1982-8.

Zeng HC, Xiao H, Wang J, Zhang M, Liu W, Wen H. Evaluation of experimentally induced early hepatic alveolar echinococcosis in rats with MRI-diffusion weighted imaging (DWI). ImE-2014: O-06.

Wang J, Ren B, Liu W, et al. Analysis between perfusion CT and microvessel density, vascular endothelial growth factor in hepatic alveolar echinococcus. Chin J Radiol 2011;45 (11):1036–1039.

Jiang Y, Wang J, Liu W. Evaluation of the HAE vascularity with energy spectral CT: correlation between iodine quantification and histopathology. ImE-2014, Besançon: P-02.

Xie W, Wang J, Liu W. The correlation between ADC and the area of fibrosis in border of hepatic alveolar echinococcosis. J Pract Diagn Ther 2012;26 (5):467–70.

Ren B, Wang J, Liu W. Comparative study between diffusion weighted imaging and histopathological features in hepatic alveolar echinococcosis. Chin J Radiol 2012;46 (1):57–61.

Wang J, Zeng HC, Chen H, Yi B, Wen H, Liu W. The comparison of MR-DWI and PET/CT in assessing the viability of hepatic alveolar echinococcosis. ImE-2014, Besançon: P-01.

#### Oral communications

##### O-01. Alveolar echinococcosis: correlation between MRI aspect of hepatic lesions and the metabolic activity visualized in FDG-PET/CT

Azizi Amel^1^, Blagosklonov Oleg^2,3,4^, Lounis Ahmed^1^, Berthet Louis^2^, Vuitton Dominique A.^4^, Bresson-Hadni Solange^4^, Delabrousse Eric^1,3,4^

^1^Department of Radiology, University Hospital, 25030 Besançon, France

^2^Department of Nuclear Medicine, University Hospital, 25030 Besançon, France

^3^EA 4662 Nanomedicine Lab, Imagery and Therapeutics, University of Franche-Comté, Besançon, France

^4^WHO Collaborating Centre for Prevention and Treatment of Human Echinococcosis, 25030 Besançon, France

amelramdani@hotmail.com; edelabrousse@chu-besancon.fr

**Background:** To correlate the appearance of Alveolar Echinococcosis (AE) hepatic lesions in Magnetic Resonance Imaging (MRI) as defined by Kodama, to the metabolic activity visualized in 18-Fluoro-DeoxyGlucose Positron Emission Tomography combined with Computed Tomography (PET/CT).

**Methods:** Forty-two patients (25 men; mean age: 62.2) diagnosed with AE and who underwent both MRI and PET/CT were included. Three independent readers blinded with regard to the PET/CT information, divided the forty-two hepatic lesions into five types according to Kodama’s classification. Concerning PET/CT, two independent readers, unaware of the MRI information, considered the results as positive when an increased FDG-uptake was observed at 1 or 3 hours after FDG injection, and as negative when no increased uptake was noted. Inter-observer agreement was assessed by using κ statistics.

**Results:** Forty-two lesions were counted and the mean diameter of overall evaluated lesions was 6.3 cm. One lesion (2.4%) was categorized as type 1, 11 (26.2%) as type 2, 24 (57.1%) as type 3, 3 (7.1%) as type 4 and 3 (7.1%) as type 5. The inter-observer analysis found a κ coefficient of 0.96. All type-1, 90.9% of type-2 and 87.5% of type-3 lesions showed an increased FDG-uptake on PET/CT images. All non-microcystic AE liver lesions (types 4, 5) showed no abnormal increased FDG uptake on PET/CT images. The inter-observer analysis at one and three hours found a κ coefficient of: 0.95 and 0.92, respectively.

**Conclusion:** In patients with AE liver lesions, the absence of micro-cysts on MRI is strongly correlated to a metabolically inactive disease.

##### O-02. Pulling alveolar echinococcosis into general radiology: two polar imaging patterns easily recognisable with a relevant impact on clinical management

Stojkovic Marija^1^, Mickan Christina^1^, Weber Tim^2^, Junghanss Thomas^1^

^1^Section Clinical Tropical Medicine, University Hospital, Heidelberg, Germany

^2^Department of Radiology, University Hospital, Heidelberg, Germany

marija.stojkovic@med.uni-heidelberg.de; thomas.junghanss@urz.uni-heidelberg.de

**Background:** Imaging plays an important role in the diagnosis and follow-up of patients with alveolar echinococcosis (AE) of the liver. The objective of this study is to illustrate the range of radiological features in AE patients relevant for differential diagnosis and treatment decision.

**Methods/Principal Findings:** 57 patients with AE of the liver managed according to the protocol of our centre were included into the study. MRI/CT scans on admission (t0) and MRI/CT on the latest (t1) follow-up were analyzed for radiological morphology of hepatic lesions, changes of lesion solidity and size, infiltration (hepatic veins, portal vein, biliary duct, caval vein, hilus and capsule of the liver) and duration of follow-up. Two major radiological patterns were observed: 1. Infiltrative lesions with a distribution (a) along the major biliary and hepatic blood vessels and (b) not associated with the major biliary and hepatic blood vessels; 2. Non-infiltrative spherical lesions.

**Conclusion:** To pull AE into general radiological practice we suggest two polar patterns which are easily recognizable with major implications on differential diagnosis and clinical decision making: Infiltrative lesions with a distribution along the major biliary and hepatic blood vessels with very few alternative differential diagnoses to AE and non-infiltrative spherical lesions which are non-specific with a wide range of differential diagnoses. With the promising endoscopic intervention to rescue the liver with biliary dilatation and stenting the recognition of the former has major implications for patients.

##### O-03. Acoustic structure quantification (ASQ): a new tool in the sonographic diagnosis of liver lesions in hepatic alveolar echinococcosis

Graeter Tilmann^1^, Kaltenbach Tanja Eva Maria^2^, Akinli Atilla Serif^2^, Kratzer Wolfgang^2^, Oeztuerk Suemeyra^2^, Haenle Mark Martin^2^, Gruener Beate^3^

^1^Department of Interventional and Diagnostic Radiology, University Hospital, Ulm, Germany

^2^Department of Internal Medicine I, University Hospital, Ulm, Germany

^3^Department of Internal Medicine III, University Hospital, Ulm, Germany

tilmann.graeter@uniklinik-ulm.de; beate.gruener@uniklinik-ulm.de

**Background:** Acoustic Structure Quantification (ASQ) is a sonographic imaging method based on B-scan. Structural changes of the hepatic parenchyma can be visualized on the basis of the statistical distribution of echo amplitudes in the tissue. The primary object of this study was the comparative analysis of the ASQ statistical parameters of mode (M), average (A), standard deviation (SD) and the focal disturbance (FD) ratio. These parameters were measured in patients with hepatic alveolar echinococcosis (HAE) in the liver lesions as well as in the adjacent normal hepatic parenchyma.

**Methods:** A total of 24 patients with HAE were examined with ASQ using a Toshiba Aplio 500 (Toshiba Medical Systems Corporation, Tokyo, Japan). The quantitative analysis (ASQ) was performed with the TusCLCQFunctio software, version 1.0.0.1. The resulting ASQ parameters (M, A, SD and FD ratio) were analyzed statistically.

**Results:** The median FD-ratio in the lesions was 3 (0.1–3), compared to 0.5 (0.1–1.8) in normal liver parenchyma (*p* < 0.0001). The statistical comparison of the other ASQ parameters showed results that are similarly significant with *p*-values ranging between *p* < 0.0001 and *p* < 0.0018.

**Discussion/Conclusion:** ASQ is a useful and promising sonographic method for the detection and quantification of structural changes of liver parenchyma in HAE lesions.

##### O-04. 1H Magnetic Resonance Spectroscopy characteristics of cerebral alveolar echinococcosis

Wang Jian^1^, Yao Weihong^1^, Liu Chen^1^, Liu Wenya^1^, Wen Hao^2^

^1^Imaging Center, First Affiliated Hospital of Xinjiang Medical University, Urumqi 830011, China

^2^Dept. of Hepatic Surgery, First Affiliated Hospital of Xinjiang Medical University, Urumqi 830011, China

jeanw1265@sina.com

**Background:** Cerebral alveolar echinococcosis (CAE) grows infiltratively like a malignant tumor, causing great harm to the human body. Xinjiang has one of the highest global incidences of AE. As the imaging features of CAE are not typical and serology of hydatid disease has false negative/positive results, this kind of disease has a high rate of misdiagnosis before surgery stage. Magnetic resonance spectroscopy (MRS) can perform quantitative studies on concentrations of metabolites and the functional status of organization and cells, thus acting as a virtual biopsy. This research focused on using proton magnetic resonance spectroscopy (1HMRS) technology to find the characteristics of CAE.

**Objective:** To evaluate the 2D multi-voxel 1H MRS Characteristics of patients with Cerebral Alveolar Echinococcosis (CAE), in order to complete lack of conventional MRI, and improve the accuracy of preoperative diagnosis.

**Materials and Methods:** 13 patients with 33 lesions histologically and clinically proven to be CAE were examined by conventional MRI and 2D multi-voxel spectra with a 3.0T double gradient superconductivity magnetic resonance scanner. Concentrations of the metabolites containing N-acetyl-aspartic-acid (NAA), Choline (Cho), Creatine (Cr), lipids and lactic acid (Lip+Lac), myo-Inositol (mI) and the value of Cho/Cr, NAA/Cr, (Lip+Lac)/Cr, mI/Cr were calculated. Concentrations of the metabolites changes were compared in the AE region with the relative contralateral part of the normal brain parenchyma area (control group). The data were statistically analyzed.

**Results:** CAE 1H MRS spectrum characteristics (in the substantial region) are as follows: Cho, NAA decreased to varying degrees in CAE 1H MRS spectrum, and were shown as a visible lipid with or without lactate peak. Compared with the control group, the value of Cho/Cr, NAA/Cr, (Lip+Lac)/Cr, mI/Cr in the AE region were 1.81 ± 0.62, 1.31 ± 0.39, 35.06 ± 13.82 and 0.94 ± 0.36, respectively; The value of Cho/Cr, NAA/Cr, (Lip+Lac)/Cr and mI/Cr of control group were 0.87 ± 0.19, 2.04 ± 0.33, 0.87 ± 0.17 and 0.25 ± 0.09, respectively; The value of the 4 group pair-wise comparisons for all above measurements were statistically significant (*P* < 0.01) between the AE region and the control group.

**Conclusions:** Multi-voxel 1H MRS can reflect pathological characteristics of CAE. 1HMRS provides metabolic information for diagnosis of CAE and may be a necessary supplement of conventional magnetic resonance examination.

##### O-05. Study of Magnetic Resonance Imaging features in brain metastases of hepatic alveolar echinococcosis

Tang Guibo, Yang Guocai, Wang Yu, He Yan, Yan Chunlong, Zhang Qingxin

Medical Imaging Center, Qinghai Provincial People’s Hospital, Qinghai Province 810007, China

qhtgb@sohu.com

**Objective:** The purpose of this study was to obtain a better understanding of brain metastasis of alveolar echinococcosis via MRI manifestation evaluation.

**Methodology:** A comprehensive analysis was conducted on twenty patients with alveolar echinococcosis by using MRI, CT, ultrasound and laboratory-related examinations. The findings were further confirmed by surgical pathology.

**Result:** All of the 20 patients with brain metastatic alveolar echinococcosis had history of primary hepatic alveolar echinococcosis. Twelve of them had received surgical treatment, while the other eight patients had undergone ultrasound-guided puncture treatment or biopsy. Out of the 20 cases, 4 of them had single lesion (20%) and 16 patients presented multiple lesions (80%). There were pulmonary metastases in 6 patients, kidney and adrenal gland metastases in 3 patients. MRI features highlighted round or irregular-shaped multiple aggregations of small vesicle-like changes, where the cross section was honeycomb shaped. T1WI and T2WI were mainly featured with low signals. The lesions were accompanied by noticeable edema. Alveolar echinococcosis embolism in hepatic vein and portal vein were found in 4 cases. MRI features of 10 patients indicated that T2WI lesions had coal-like low-signal shadow, with multiple small vesicles inside the lesions. The MRS performance suggested significant reduction of NAA, Cho and Cr, associated with an abnormally high and steep crest at 1.4 ppm. The phase diagram and strength diagram of SWI presented iso-intensity.

**Conclusion:** Brain is one of the most vulnerable organs where hepatic alveolar echinococcosis may occur. MRI manifestations such as honeycomb-shaped small vesicles and T1WI/T2WI low signals are distinguishing characteristics of brain metastasis of alveolar echinococcosis. Moreover, MRS and SWI could provide supplementary information to diagnosis.

##### O-06. Evaluation of experimentally induced early hepatic alveolar echinococcosis in rats with Magnetic Resonance-Diffusion Weighted Imaging (DWI)

Zeng Hongchun^1^, Xiao Hu^2^, Wang Junhua^3^, Zhang Mei^4^, Liu Wenya^2^, Wen Hao^3^

^1^Department of Ultrasonography, First Affiliated Hospital of Xinjiang Medical University, Urumqi, Xinjiang 830011, China

^2^Imaging center, First Affiliated Hospital of Xinjiang Medical University, Urumqi, Xinjiang 830011, China

^3^Xinjiang Key Lab of Fundamental Medical Research and Xinjiang Hydatid Clinical Research Institute, First Affiliated Hospital of Xinjiang Medical University, Urumqi, Xinjiang 830011, China

^4^Department of Radiology, Affiliated Traditional Chinese Medical Hospital of Xinjiang Medical University, Urumqi, Xinjiang 830011, China

hongchunzeng@yahoo.cn; dr_lwyykdx@163.com

**Background:** The typical features of Hepatic alveolar echinococcosis (HAE) by ultrasound (US), computed tomography (CT) and magnetic resonance imaging (MRI) have been reported. However, early diagnosis is often difficult by imaging because of atypical features. In light of the severity of HAE, it is worthwhile to obtain new imaging techniques to diagnose the disease at an early stage, when surgery is feasible to cure the patient. Although MRI had wide use for diagnosing HAE, the features of early hepatic alveolar echinococcosis in MRI have not been known well. The aim of this study focused on demonstrating the features of early HAE in rat using MRI-DWI.

**Methods:** The experimentally induced HAE in 40 rats were studied after testified by US. All rats were studied on a 3T Unit (GE 3. 0 T Signa Eexcite) with a wrist coil. All rats were anesthetized, and were laid in wrist coil. After adjusting the different scan conditions in order to determine the most appropriate scan parameters, routine T1W1, T2W1 and DWI were performed. After MRI, all rats were sacrificed by excessive anesthesia, and the livers were removed for pathological exam.

**Results:** The best image quality and highest detection rate of early HAE lesions of rat with DWI can be obtained with *b* = 500 in DWI. Thirty-one lesions were found in 40 rats by MRI. MRI failed to detect lesions in 9 rats because of size smaller than 2.7 mm. All lesions were low signal compared with the liver parenchyma in T1-weighted MR images, and high signal in T2-weighted MR images. T2-weighted images showed multiple vesicles presented as round high signal in 16 lesions. All lesions showed a ring of lower signal compared to internal lesions in DWI imaging. ADC values of marginal zone between the lesion and hepatic parenchyma were lower than in the center of lesions, and higher than in surrounding liver parenchyma. Comparing the DWI imaging with pathologic features, the lower signal surrounding the lesion corresponded to the inflammatory belt, which was rich in micro-blood vessels and fibrotic tissue, as well as inflammatory cells.

**Conclusion:** The results suggested that MRI-DWI can demonstrate the characteristic features in early HAE in rat. MRI-DWI can be recognized as a diagnostic method for early HAE lesions in rat.

##### O-07. Alveolar echinococcosis metabolic imaging: from in vitro testing to small animal Positron Emission Tomography*

Porot Clémence^1,2^, Knapp Jenny^1^, Wang Junhua^1,3^, Camporese David^4^, Germain Stéphane^5^, Boulahdour Hatem^1^, Seimbille Yann^6^, Gottstein Bruno^3^, Vuitton Dominique A.^2^, Blagosklonov Oleg.^1,2^

^1^Dept. of Nuclear Medicine, University Hospital, 25030 Besançon, France

^2^WHO Collaborating Centre for Prevention and Treatment of Human Echinococcosis, University Hospital and University of Franche-Comté, 25030 Besançon, France

^3^Institute of Parasitology, University of Bern, CH-3001 Bern, Switzerland

^4^Advanced Accelerator Application SA, 01630 Saint Genis Pouilly, France

^5^Cyclotron unit, University Hospital of Geneva, 4205 Geneva, Switzerland

cporot@chu-besancon.fr; oleg.blagosklonov@univ-fcomte.fr

**Background:** Positron emission tomography with [18F]-fluorodeoxyglucose ([18F]-FDG-PET) including delayed acquisition is a valuable method for initial staging and follow-up of patients with alveolar echinococcosis (AE). However, the cells responsible for [18F]-FDG uptake have never been formally identified. The main goal of the IsotopEchino Project was to identify such cells and to test *in vitro* the most widely used fluorinated tracers in order to provide clinicians with more specific staging tool of AE.

**Methods:** We designed a radiolabelling protocol which could be transposed to each type of cells composing the AE host-parasite interface. Candidate molecules – [18F]-fluorotyrosine (FET), [18F]-fluorothymidine (FLT), [18F]-fluorometylcholine (FMC), and sodium [18F]-fluorine (NaF) – were tested and compared to [18F]-FDG *in vitro* with human leukocytes, human hepatocytes and *in vitro* cultivated *E. multilocularis* vesicles. Each experiment was performed in triplicate. We determined mean radiolabelling efficiency (RE, in %) and mean uptake by volume of cells (MBq/μm^3^) from 3 tests.

**Results:** As anticipated, [18F]-FDG was mainly uptaken by periparasitic immune cells and showed that [18F]-FLT (a proliferation tracer) was the best candidate tracer for parasite metabolism. Based on average uptake value by cell volume, human white blood cells were 1,000–10,000 times more avid for fluorinated tracers than *E. multilocularis* vesicles. Leukocytes had the worst RE with [18F]-FLT (6%) and the best one with [18F]-FDG (52%). In parasitic vesicles, [18F]-FLT showed the highest radiolabelling efficiency (93%); RE with FDG was poor (32%). The difference of RE with other tracers was not discriminant between the parasitic vesicles and human leukocytes. As [18F]-FLT might be suitable for direct functional imaging of AE, further in vivo experiments were performed: [18F]-FDG-PET/CT and [18F]-FLT-PET/CT scans were performed in mice intraperitoneally infected with *E. multilocularis* metacestodes at late infection stage. We observed a moderate [18F]-FDG uptake by immune cell granuloma-rich parasitic lesions and no [18F]-FLT uptake by alveolar echinococcosis lesions.

**Discussion/Conclusion:** Our study confirmed *in vitro* and *in vivo* the role of inflammatory periparasitic process in [18F]-FDG–PET imaging of AE. Furthermore, this study showed that none of the four tested markers was more efficient than [18F]-FDG. Assessment of PET in experimental mice still requires further studies. A specific radio-immunotracer could be the next step for the development of a specific PET tracer for AE lesions in order to improve detection and treatment of human echinococcosis.

*Research supported by Operational Program for cross-border cooperation INTERREG IVA France-Switzerland 2007–2013; project “IsotopEchino”.

#### Posters

##### P-01. The comparison of MR-DWI and PET/CT in assessing the viability of hepatic alveolar echinococcosis

Wang Jing^1^, Zeng Hongchun^2^, Chen Hong^1^, YI Banu^1^, Wen Hao^3^, Liu Wenya^1^

^1^Imaging Center, First Affiliated Hospital of Xinjiang Medical University, Urumqi 830054, China

^2^Department of abdominal ultrasound, First Affiliated Hospital of Xinjiang Medical University, Urumqi 830054, China

^3^Department of Liver and Laparoscopy Surgery, First Affiliated Hospital of Xinjiang Medical University, Urumqi 830054, China

wjing7997@163.com; wenyaliu2002@163.com

**Background:** MRI with its conventional sequences, such as T1WI, T2WI and enhanced T1-weighted sequences, had been used in diagnosing hepatic alveolar echinococcosis (HAE) and the corresponding complications in some studies. To our knowledge, however, a very important MR sequence, diffusion-weighted imaging (DWI), was already widely established in diagnosing the hepatic occupying lesions, is rare used in HAE. The purpose of our search is to reveal DWI in evaluation of HAE viability by comparing DWI with PET/CT.

**Methods:** 18F-FDG-PET/CT and DWI (*b*-values, 0, 800 s/mm^2^) were retrospectively analyzed in 8 patients with clinically verified HAE, and the ADC map was generated consequently. The metabolic activity of HAE lesions in both techniques were determined by two independent radiologists according to the following grade Standard: (++) marked focally or perilesionally increased FDG uptake/hyper-signal intensity, (+) slightly focally or perilesionally increased FDG uptake/hyper-signal intensity, (−) a hepatic defect without FDG uptake/no any hyper-signal intensity, in PET/CT and DWI respectively. Pearson’s correlation coefficient was assessed between the results of two observers.

**Results:** 16 lesions (composed of 14 HAE and 2 cystic echinococcosis, CE) were detected, 8 lesions (diameter more than 2 cm) showed peri-lesional hyper-signal intensity in DWI, which could be visualized in PET/CT as increased FDG uptake, mainly existed in the lesion’s border with normal liver parenchyma. 5 lesions were detected as nodular hyper-signal intensity in DWI and “hot spot” in PET/CT in the same distribution. 1 cases had oral drug therapy for three years, the peri-lesional hyper-signal intensity in DWI persisted but had significantly decreased and not continuous than the image taken at initial diagnose, while, the lesion presented as hepatic defect without any FDG uptake in post-treatment PET/CT. 2CE lesions showed negative viability in both DWI and PET/CT. The k-value of 0.88 (*P* < 0.01) indicated a good concordance between DWI and PET/CT in depicting the metabolic activity of HAE.

**Conclusion:** This preliminary study is the first to show the value of DWI in assessing HAE viability, DWI should be routinely used in HAE evaluation (at initial and treatment follow-up).

##### P-02. Assessment of disease vascularity in alveolar echinococcosis with Dual Energy CT: a correlation between iodine quantification and histopathologic parameters

Jiang Yi, Liu Wenya

Imaging Center, First Affiliated Hospital of Xinjiang Medical University, Urumqi, Xinjiang 830054, China

jackyyi1001@gmail.com; wenyaliu2002@163.com

**Background:** To date, there is rare related report for accurate assessment of vascularization of hepatic lesions in patients who have alveolar echinococcosis (AE) due to the difficulty of gaining the reliable quantitative indicators related to poor vascularization of the parasitic mass using traditional image equipment. Dual-energy CT (DECT) allows quantification of intravenously injected iodinated contrast media in lesions, and therefore may be considered as a surrogate marker for perfusion and tumor vascularity. Thus it is meaningful to use DECT to evaluate the vascularization of AE and thereby to indirectly depict parasitic activity.The objective of the present study was to investigate the correlation between DECT quantitative iodine concentration with microvascular density (MVD) in alveolar echinococcosis lesions.

**Methods:** In this prospective study, 24 patients with confirmed hepatic alveolar echinococcosis (HAE) were included (11 female and 13 male; average age, 52 ± 14 years) and were then examined using dual-source mode (100 kV/140 kV). The CT pattern of HAE lesions was classified into three types: solid form (6 cases), pseudocystic form (4 cases) and “geographic map” (mixed) form (14 cases). Regions of interest were placed on the iodine image over marginal zone, solid and cystic component of the lesion while avoiding calcification. For the analysis, two investigators measured the following parameters of HAE lesion in the artery, portal vein phases: CT attenuation value in Hounsfield units (HU) and iodine concentration (mg/ml). MVD was detected by using anti-CD34 monoclonal antibody within each lesion. Statistical analyses were performed using the Spearman rank correlation and the Mann–Whitney t-test.

**Results:** The lesion iodine concentration in marginal zone was significantly higher than in solid and cystic component (*p* < 0.0001). The iodine concentration measurements were significantly different between marginal zone and solid component of HAE lesion both in the artery phase (1.63 mg/ml vs. 0.42 mg/ml, *p* = 0.001) and in the portal vein phase (1.84 mg/ml vs. 0.62 mg/ml, *p* = 0.001), while mean attenuation values were not significantly different in both phases (55.4 HU vs.47.2 HU, *p* = 0.063 and 64.4 HU vs. 52.6 HU, *p* = 0.052, respectively). There was excellent correlation between iodine concentration measurements and MVD (*r* = 0.940, *p* < 0.05) in marginal zone of HAE lesion.

**Conclusions:** DECT quantitative iodine concentration was significantly correlated with MVD. Dual-energy CT using a quantitative analytic methodology can be used to evaluate the vascularity of AE.

##### P-03. New CT-classification of hepatic alveolar echinococcosis

Graeter Tilmann^1^, Kratzer Wolfgang^2^, Oeztuerk Suemeyra^2^, Junghanns Florence^2^, Haenle Mark Martin^2^, Akinli Atilla Serif^2^, Kern Peter^3^, Gruener Beate^4^

^1^Department of Interventional and Diagnostic Radiology, University Hospital, Ulm, Germany

^2^Department of Internal Medicine I, University Hospital, Ulm, Germany

^3^WHO Informal Working Group on Echinococcosis & Comprehensive Infectious Diseases, Ulm University, Ulm, Germany

^4^Department of Internal Medicine III, University Hospital, Ulm, Germany

tilmann.graeter@uniklinik-ulm.de; beate.gruener@uniklinik-ulm.de

**Background:** Computed tomography, mostly combined with PET, provides one of the most important diagnostic tools in suspected alveolar echinococcosis. Aim of the study was to establish a new CT-classification based on a large patient collective with confirmed hepatic alveolar echinococcosis.

**Methods**: In 224 patients, CT-morphology of liver lesions due to an alveolar echinococcosis was retrospectively examined. The findings were grouped into the new classification scheme.

**Results:** Within the classification a lesion was dedicated to a “primary morphology” as well as to a “pattern of calcification”. The primary morphology distinguishes following types: I. Diffuse infiltrating (with/without cystoid portion), II. Primarily circumscribed tumor-like (with/without cystoid portion and with/without offshoot at the edge), IIIa. Primarily cystoid, intermediate (with/without more solid portions at the edge), IIIb. Primarily cystoid widespread (with/without more solid portions at the edge), IV. Small-cystoid/metastatic* and V. Mainly calcified. Except for the “primary morphology” type V., following patterns of calcification were attributed additionally: without calcifications; with feathery calcifications; with focal (p.r.n. central – just possible with*) calcifications; with diffuse calcifications; with calcifications primarily at the edge. The various classification patterns are demonstrated by image examples.

**Conclusion**: The proposed CT-morphological classification will facilitate the interpretation of lesions due to a hepatic alveolar echinococcosis. This could help to interpret different clinical courses better and will assist in the context of scientific studies to improve the comparability of CT findings.

##### P-04. Comparison of parametric contrast enhanced ultrasound with quantified PET-CT in determining the vitality of liver lesions by alveolar echinococcosis

Graeter Tilmann^1^, Kaltenbach Tanja Eva Maria^2^, Akinli Atilla Serif^2^, Kratzer Wolfgang^2^, Oeztuerk Suemeyra^2^, Haenle Mark Martin^2^, Gruener Beate^3^

^1^Department of Interventional and Diagnostic Radiology, University Hospital, Ulm, Germany

^2^Department of Internal Medicine I, University Hospital, Ulm, Germany

^3^Department of Internal Medicine III, University Hospital, Ulm, Germany

tilmann.graeter@uniklinik-ulm.de; beate.gruener@uniklinik-ulm.de

**Objective:** Objective of the study was to qualitatively and quantitatively compare contrast enhanced ultrasound (CEUS) and F-18-FDG-PET-CT in the follow-up of hepatic alveolar echinococcosis (HAE).

**Methods:** 36 patients with proven, medically-treated HAE have been included in this study. Abdominal ultrasound and CEUS have been carried out using the ultrasound contrast amplifier SonoVue^®^. As part of the monitoring, patients were examined by F-18-FDG-PET-CT. Quantitative analysis of CEUS was performed using the VueBox™ Quantification Toolbox. Maximum contrast enhancement in lesions (“peak enhancement”, PE) was used as a measurement parameter. For quantification of F-18-FDG-PET-CT, the maximum Standardized Uptake Value (SUV_max_) of lesions was determined and compared with PE.

**Results:** F-18-FDG uptake in parasitic lesions was detected by F-18-FDG-PET-CT in 32 of 36 patients. Vascularization of liver lesions was detected by CEUS in 22 of 32 FDG-positive patients (sensitivity, 69%; specificity, 100%). Doppler Ultrasound detected vascularized lesions with a sensitivity of 25% and specificity of 75%. Mean maximum diameter of lesions was 69.5 mm in CEUS and 63.7 mm in B-scan ultrasound (*p* < 0.0001). No significant correlation was found between SUV_max_ and PE (*p* = 0.8879).

**Conclusion:** Compared with F-18-FDG-PET-CT as the gold standard in determining the metabolic activity of HAE liver lesions, CEUS visualizes the vascularization of active lesions with a high specificity and moderate sensitivity. CEUS must therefore be considered an important tool in monitoring HAE. Dimensions of parasitic lesions are displayed more precisely through CEUS than in B-scan. Compared with currently available methods, CEUS quantification provides no additional benefit in routine monitoring HAE lesions.

##### P-05. New ultrasonographic classification of hepatic alveolar echinococcosis

Graeter Tilmann^1^, Kratzer Wolfgang^2^, Oeztuerk Suemeyra^2^, Junghanns Florence^2^, Haenle Mark Martin^2^, Akinli Atilla Serif^2^, Kern Peter^3^, Gruener Beate^4^

^1^Department of Interventional and Diagnostic Radiology, University Hospital, Ulm, Germany

^2^Department of Internal Medicine I, University Hospital, Ulm, Germany

^3^WHO Informal Working Group on Echinococcosis & Comprehensive Infectious Diseases, Ulm University, Ulm, Germany

^4^Department of Internal Medicine III, University Hospital, Ulm, Germany

tilmann.graeter@uniklinik-ulm.de; beate.gruener@uniklinik-ulm.de

**Background:** Ultrasonography provides one of the most important diagnostic tools in suspected alveolar echinococcosis. Aim of the study was to establish a new sonographic classification based on a large patient population with confirmed hepatic alveolar echinococcosis.

**Methods:** In 225 patients, ultrasound morphology of liver lesions due to an alveolar echinococcosis was retrospectively examined. The findings were grouped into the new classification scheme.

**Results:** The following classification has been established: storm and hail pattern, pseudo hemangioma-like pattern, ossification pattern and metastasis-like pattern. The respective classification patterns are demonstrated by imaging examples.

**Conclusion:** The proposed ultrasonographic classification improves the diagnosis of hepatic alveolar echinococcosis. This makes it possible to interpret different clinical courses better and helps in the context of scientific studies to improve the comparability of ultrasonographic findings.

##### P-06. The diagnostic value of PET/CT imaging in hepatic alveolar echinococcosis and its biology boundary

Qin Yongde, Xie Bin, Li Xiaohong

Department of nuclear medicine, First Affiliated Hospital of Xinjiang Medical University, Urumqi, China

qyd199013@163.com

**Background and aims:** To analyze the biology boundary of the hepatic alveolar echinococcosis (AE) with PET/CT and improve the diagnostic value of PET/CT by comparing PET/CT images with pathology findings.

**Methods:** Among patients whose diagnosis of AE was confirmed, 23 cases in our hospital (male: 14 cases, female: 9 cases, age 40 ± 25 years old; 27 lesions in total) have taken the PET/CT examinations. All the patients underwent surgical treatment. We analyzed the performance of PET/CT imaging, and then compared with pathology findings.

**Results:** The distribution of radiotracer was non-uniform in the liver, and sometimes, there was no uptake of radiotracer in the lesion, but around the lesion the uptake was obvious; so, 18F-FDG can draw the biology boundary of the lesion, and then show the characteristics of radiopharmaceuticals uptake in the boundary. SUVmax values were between 3.7 ± 0.9, showing the characteristics of biological activity. Two hours later, the delayed phase showed that the up-taking of radiopharmaceuticals was increased, SUVmax values were between 4.7 ± 1.2, the SUV values of every lesion increased compared to the initial phase. Statistical analysis between the biology boundary of AE lesion and pathology findings showed a sensitivity of 95.45% (21/22), a specificity of 60% (3/5), an accuracy of 88.89% (24/27), a positive predictive value of 91.30% (21/23), and a negative predictive value of 75% (3/4).

**Conclusions:** PET/CT not only can detect the lesion location, shape, number, border, calcification and the surrounding tissue, but also can have diagnostic value for the activity characteristics of the lesions and development process, thus the prognosis.

##### P-07. Imaging evaluation of hepatic cystic echinococcosis biological activities and outcome

Tang Guibo, Yang Guocai, Wang Yu, He Yan, Yan Chunlong, Zhang Qingxin

Medical Imaging Center, Qinghai Provincial People’s Hospital, Qinghai Province 810007, P.R. China

qhtgb@sohu.com

**Objective:** The study aimed to evaluate and analyze the biological activities and natural course of hepatic cystic echinococcosis by applying imaging technology.

**Methodology:** A comprehensive analysis was performed with respect to 300 patients with hepatic cystic echinococcossis and imaging features collected by CT, MRI and Ultrasound. The diagnosis of echinococcosis was confirmed by surgery pathology.

**Result:** Out of the 300 patients, there were 98 patients with single cysts, 54 patients with daughter cysts, 40 patients with anechoic content with detachment of laminated membrane from the cyst wall and 108 patients with solid cysts.

**Conclusion:** The biological activities of echinococcosis are closely related with its growth pattern, parasitic duration and imaging classification.

##### P-08. Sonographic classification of hepatic hydatid cyst and its therapeutic implication

Alkhouja Alaa, Talioua Lamiae, Afifi Rajaa, Benazzouz Mustapha, Essaid Abdellah

Gastroenterology Dept. –Medical C, CHU Ibn Sina, Rabat, Morocco

feydi2001@yahoo.fr; alaa.elkhouja@gmail.com

**Background:** Hydatid cyst is a parasitosis which can affect all the viscera. The hepatic localization is the most frequent. There are several classification schemes for liver hydatid cysts based on their ultrasound appearances, the initial classification by Gharbi remains the most used. Our study aims to highlight the contribution of ultrasound in both diagnosis and percutaneous treatment of hepatic hydatid cyst (HHC) through the experience of our unit.

**Materials and Methods:** This is a descriptive retrospective study including 183 cases of HHC performed at the gastroenterology service –Medical C-Ibn Sina Rabat during a 5-years period between 2009 and 2013. For each patient, epidemiological and clinical data, localization of HHC, size and Gharbi grade, therapeutic and evolutionary measures were collected.

**Results:** Our study included 128 patients, 46 men and 82 women with a sex ratio of 0.56, diagnosed with 183 hydatid cysts. The average age was 37 years [15–81 years], Twenty five percent of our patients have a history of previous HHC surgery. The discovery circumstances were dominated by atypical abdominal pain in 77% of cases and jaundice in 9% of cases. The cysts were located on the right liver in 80.3%, on the left liver in17.4% and bilobar in 2.3% of cases. Extrahepatic localization was noted in 6 patients: pulmonary in 3 patients and peritoneal and splenic in 3 patients. The average diameter of cysts was 83.7 mm [15–200 mm]. Sixty three percent of cysts were Gharbi grade I, 15% grade II, 12% grade III, 8% grade IV and 2% grade V. A PAIR (puncture-aspiration-injection and re-aspiration) was realized in 52.5% of cases (83% of them grade I and 17% grade II), percutaneous drainage in 13% of cases for complicated HHCs (fistulated or infected), endoscopic sphincterotomy in 1.6% of cases, surgical treatment in 22% of cases (63% grade III and 37% grade I–II superficially localized cysts) and surveillance in 7% of cases grade IV–V). Evolution for hydatid cysts treated by percutaneous technique was favorable with a success rate of 89% held by a decrease of more than 50% in cyst size or its solidification during US follow up.

**Conclusion:** Abdominal ultrasound plays a fundamental role in the diagnostic and therapeutic approach to hepatic hydatid cyst by providing an alternative of surgical treatment through the percutaneous ultrasound-guided treatment. Our study included patients primarily classified grade I-II, according to the classification of Gharbi, and was efficient in 89% of cases.

##### P-09. Direct parasitological examination *vs* ultrasonography in the diagnosis of cystic echinococcosis in sheep

Scala Antonio, Dore Francesco, Pipia Anna Paola, Moi Michela, Sanna Giuliana, Tamponi Claudia, Corda Andrea, Pinna Parpaglia Maria Luisa, Nieddu Daniela, Varcasia Antonio

Dipartimento di Medicina Veterinaria, Università degli Studi di Sassari, Sassari, Italy

scala@uniss.it

**Background:** Ultrasonography has already been evaluated as diagnostic tool for CE in humans and also for intra vitam screening in sheep. However, the recent advance of imaging technology has led to a drastic improvement of the performance of portable ultrasound, e.g. by allowing monitoring of the full liver parenchyma in live animals with a microconvex transducer. Therefore, the aim of this study was to evaluate ultrasonography as an intra vitam screening tool for ovine CE under field conditions. For this reason, a survey for cystic echinococcosis (CE) diagnosis in sheep was carried out in Sardinia.

**Methods:** The study was carried out in three farms: farm A, (Municipality of Nule, Sassari), farm B (Municipality of Sassari), farm C (Municipality of Monastir, Cagliari) which had been pre-selected according to different levels of prevalence for CE (A: >80%, B: 50–80%, C: <50%). A total of 129 sheep were examined (A: *n* = 51, B: *n* = 30, C: *n* = 48) and ultrasounds were performed with sheep in an upright position without any sedation. Within 20 days after the ultrasound diagnosis sheep were slaughtered and a post-mortem examination diagnosis was carried out in the liver and lungs.

**Results:** Comparing ultrasonography *vs* post mortem examination, a sensitivity of 88.7% and a specificity of 75.9% of ultrasound were found, while the positive and negative predictive values were 81.8% and 84.6%, respectively. The sensitivity of the test increased to 100% if we consider only fertile cysts.

**Discussion:** Ultrasonography of the liver in sheep can be considered a useful intra vitam diagnostic tool to identify CE positive animals and it could be an important instrument as part of a program of epidemiological surveillance for control plans of this important metacestodosis, like vaccination trials.

### 1.B. Biological tools for diagnosis and follow-up

#### State-of-the-art

##### L-02. Viable or non-viable, that is the question!

Gottstein Bruno

Institute of Parasitology, Vetsuisse Faculty and Faculty of Medicine, University of Bern, Bern, Switzerland

bruno.gottstein@ipa.unibe.ch

In infected humans the *E. multilocularis* metacestode (larva) develops primarily in the liver. The typical proliferating lesion appears microscopically as a conglomerate of small vesicles and cysts composed of a thin outer (PAS-positive) laminated layer and an inner germinal, which represents the actual living part of the metacestode. A granulomatous host reaction surrounds the metacestode, including a vigorous synthesis of fibrous and germinative tissue. In contrast to infections in susceptible rodent hosts, lesions from infected human patients rarely exhibit brood capsule and protoscolex formation within vesicles. The kind of immune response developed by the host accounts for the subsequent dichotomy concerning the parasite development (Stojkovic et al., 2013): (i) resistance as shown by the presence of “dying out” or “aborted” metacestodes; (ii) controlled susceptibility as shown by a slowly growing metacestode tissue – this group refers to the normal AE patients who first experience clinical signs and symptoms 5–15 years after infection, and (iii) uncontrolled hyperproliferation of the metacestode due to an impaired immune response (AIDS or other immunodeficiencies, e.g. following orthotropic liver transplantation). The host immune mechanisms modulating the course of infection include primarily T cell interactions. Thus, the periparasitic granuloma contains a large number of CD4^+^ T cells in patients with abortive or died-out lesions, whereas in patients with active metacestodes the number of CD8^+^ T cells is increased (Vuitton et al., 2006). The parasitic metacestode himself seems to initiate, predominantly by means of bioactive metabolites, immunosuppressive and/or immunoregulatory processes that are assumed to correlate to parasite survival and proliferation dynamics. Cytokine mRNA levels during AE show initially elevated transcription levels of pro-inflammatory cytokines, e.g. IL-1 beta, IL-6 and tumour necrosis factor alpha (TNF-alpha), which are gradually re-oriented towards Th2, including elevated IL-3, IL-4 and IL-10 as well as TGF-beta transcripts (Vuitton et al., 2006). Thus, TGF-beta-driven regulatory T cells are thought to play a crucial role in the parasite-modulated progressive course of AE (Mejri et al., 2011). The response characteristics of AE-resistant persons could so far not be elucidated. Conversely, impairment of Th cell activity such as in advanced AIDS or other immunological disorders is associated with a rapid and unlimited growth and dissemination of the parasite in AE. CD4^+^ recovery in AIDS patients by means of appropriate therapy, however, reinstates control over progression of AE treated with benzimidazoles (Zingg et al., 2004).

From the clinical point of view, in vivo assessment of the metacestode activity status is essential in view to design an optimal individual treatment strategy for a given AE-patient. Laboratory testing of parasite viability can be performed with RT-PCR of biopsies and fine-needle aspirates (Diebold-Berger et al., 1997) upon various constitutively expressed gene targets (e.g. 14-3-3), within the limits of the sensitivity of this method (Zhang et al., 2003; Matsumoto et al., 2006). However, such an approach is not been validated for routine monitoring of the course of treated AE-patients.

Better than for CE, some specific serologic tests are valuable to assess the efficacy of treatment in combination with imaging during follow-up of patients. After successful surgery and/or chemotherapy leading to inactivation of the parasite, anti-Em18 (and to a certain extent anti-Em2^+^) antibodies decline is rapid, and seroconversion to undetectable levels correlates well with curative resection (Ammann et al 2004; Tappe et al 2009). Prospectively, there is a remarkably strong demand by clinicians for improved imaging tools to assess in vivo the viability or non-viability status of treated hepatic and extra-hepatic AE-lesions. [18F]-fluorodeoxyglucose (FDG) is a validated tracer of AE lesions; however, it does not directly reflect parasite viability but rather peri-parasitic host inflammatory processes. The ideal tracer should be able to assess the course of AE upon direct uptake by the metacestode through its metabolic activity (Porot et al., 2013). The search for such new methodologies will require appropriate animal models suitable to be tested by micro-PET CT or MRI analyses.

**References**

Ammann RW, Renner EC, Gottstein B, Grimm F, Eckert J, Renner EL, Swiss Echinococcosis Study Group: Immunosurveillance of alveolar echinococcosis by specific humoral and cellular immune tests: prospective long-term analysis of the Swiss chemotherapy trial (1976–2001). J Hepatol. 2004; 41: 551–559

Diebold Berger S, Khan H, Gottstein B, Puget E, Frossard JL, Remadi S. Cytologic diagnosis of isolated pancreatic alveolar hydatid disease with immunologic and PCR analyses – A case report. Acta Cytologica 1997;41: 1381–1386

Matsumoto J, Müller N, Hemphill A, Oku Y, Kamiya M, Gottstein B. 14-3-3- and II/3-10-gene expression as molecular markers to address viability and growth activity of *Echinococcus multilocularis* metacestode. Parasitology 2006;132: 83–94

Mejri N, Mueller J, Gottstein B: Intraperitoneal murine *Echinococcus multilocularis* infection induces differentiation of TGF-β expressing DCs that remain immature. Parasite Immunology 2011;33: 471–482

Porot C, Wang J, Germain S, Seimbille Y, Camporese D, Knapp J, Vuitton DA, Blagosklonov O, Gottstein B: In vitro and in vivo investigations to develop functional imaging by Positron Emission Tomography (PET) for murine and human alveolar echinococcosis. Abstract book, 24th WAAVP Meeting, Perth, Australia, 2013.

Stojkovic M, Gottstein B, Junghanns T. Echinococcosis. In: Manson’s Tropical Diseases, 23rd edition. Eds: Farrar J, Hotez PJ, Junghanss T, Kang G, Lalloo D, White N. Elsevier Saunders, 2014; pp. 795–819

Tappe D, Sako Y, Itoh S, Frosch M, Grüner B, Kern P, Ito A. Immunoglobulin G Subclass Responses to Recombinant Em18 in the Follow-Up of Patients with Alveolar Echinococcosis in Different Clinical Stages. Clin Vaccine Immunol. 2010;17: 944–948

Vuitton D, Zhang SL, Yang Y, Godot V, Beurton I, Mantion G, Bresson-Hadni S. Survival strategy of *Echinococcus multilocularis* in the human host. Parasitol Int 2006:Suppl:S51–55.

Zingg W, Renner-Schneiter EC, Pauli-Magnus C, Renner EL, van Overbeck J, Schläpfer E, Weber M, Weber R, Opravil M, Gottstein B, Speck RF, and the Swiss HIV Cohort Study: Alveolar echinococcosis of the liver in an adult with human immunodeficiency virus type-1 infection. Infection 2004;32: 299–302

Zhang W, Li L, McManus DP: Concepts in immunology and diagnosis of hydatid disease. Clin Microbiol Rev 2003;16: 16–36

#### Key-note

##### L-03. *Echinococcus granulosus* genomics; an opportunity to improve diagnosis, treatment and control of echinococcosis

McManus Donald P.^1^, Zhang Wenbao^2^, Wang Shengyue^3^

^1^Molecular Parasitology Laboratory, QIMR Berghofer Institute of Medical Research, Brisbane, Queensland, Australia

^2^State Key Laboratory Incubation Base of Xinjiang Major Diseases Research, Clinical Medical Research Institute, First Affiliated Hospital of Xinjiang Medical University, Urumqi, China

^3^Shanghai-Ministry of Science and Technology Key Laboratory of Health and Disease Genomics & Chinese National Human Genome Center at Shanghai, Shanghai, China

donM@qimr.edu.au

In 1984, one of us (DPM) undertook a working sabbatical at the National Institute for Medical Research, Mill Hill, London, UK learning new techniques in molecular biology; skills and training vital to future research on echinococcosis. Three papers resulted. One described the first successful isolation of functional DNA and RNA from the *Echinococcus* tapeworms (McManus et al., 1985), a study that pioneered many subsequent molecular studies on cestodes. The second publication was the first to show the applicability of molecular methods, through the use of the restriction fragment polymorphism (RFLP) analysis, for unambiguous identification of *Echinococcus* species and strains (McManus and Simpson, 1985). This is an area that has subsequently blossomed into the burgeoning field of strain characterisation, genotyping and genetic epidemiology of *E. granulosus* and *E.multiolocularis*, especially following the later advent and use of the polymerase chain reaction and related procedures. The third article was the first to describe the cloning of genomic DNA from *E. granulosus* (Rishi and McManus, 1987); the cloning of *Echinococcus* cDNAs soon followed, opening up cestode biology to the full armamentarium of genetic engineering, transcriptomics and other omics tools. This culminated in 2013 in two complementary landmark and revolutionary papers on *Echinococcus* genomes. Tsai et al. (2013) described a high-quality genome for *E. multilocularis*, together with draft genomes of three other tapeworm species including *E. granulosus.* Zheng et al. (2013) reported the sequence and analysis of the *E. granulosus* genome. The two studies provide a rich source of information that provide new insights into the biology, differentiation, development, evolution, mechanisms of pathogenesis and host interaction of *E. multilocularis* and *E. granulosus.* Further, these comprehensive data sets can facilitate the development of urgently needed new echinococcosis public health intervention tools given the inefficiencies of currently available drugs, the lack of appropriate diagnostic procedures and the current difficulties in treatment and control.

**References**

McManus DP, Knight M and Simpson AJG. Isolation and characterisation of nucleic acids from the hydatid organisms, *Echinococcus* spp. (Cestoda). Mol Biochem Parasitol 1985;16: 251–266.

McManus DP and Simpson AJG. Identification of the *Echinococcus* (hydatid disease) organisms using cloned DNA markers. Mol Biochem Parasitol 1985;17: 171–178.

Rishi AK and McManus DP. Genomic cloning of human *Echinococcus granulosus* DNA: isolation of recombinant plasmids and their use as genetic markers in strain characterization. Parasitology 1987;94: 369–383.

Tsai IJ, Zarowiecki M, Holroyd N, Garciarrubio A, Sanchez-Flores A, Brooks KL, Tracey A, Bobes RJ, Fragoso G, Sciutto E, Aslett M, Beasley H, Bennett HM, Cai J, Camicia F, Clark R, Cucher M, De Silva N, Day TA, Deplazes P, Estrada K, Fernández C, Holland PW, Hou J, Hu S, Huckvale T, Hung SS, Kamenetzky L, Keane JA, Kiss F, Koziol U, Lambert O, Liu K, Luo X, Luo Y, Macchiaroli N, Nichol S, Paps J, Parkinson J, Pouchkina-Stantcheva N, Riddiford N, Rosenzvit M, Salinas G, Wasmuth JD, Zamanian M, Zheng Y; Taenia solium Genome Consortium, Cai X, Soberón X, Olson PD, Laclette JP, Brehm K, Berriman M. The genomes of four tapeworm species reveal adaptations to parasitism. Nature 2013;496: 57–63.

Zheng H, Zhang W, Zhang L, Zhang Z, Li J, Lu G, Zhu Y, Wang Y, Huang Y, Liu J, Kang H, Chen J, Wang L, Chen A, Yu S, Gao Z, Jin L, Gu W, Wang Z, Zhao L, Shi B, Wen H, Lin R, Jones MK, Brejova B, Vinar T, Zhao G, McManus DP, Chen Z, Zhou Y, Wang S. The genome of the hydatid tapeworm *Echinococcus granulosus*. Nature Genetics 2013;45: 1168–1175.

#### Oral communications

##### O-08. Circulating Antigen B in cystic echinococcosis patients antibody-negative against hydatid cyst fluid antigens

Li Jun^1,2^, Zhang Wenbao^1^, Lin Renyong^1^, Wang Hui^1^, Li Liang^1^, Wang Junhua^1^, McManus Donald P.^2^, Wen Hao^1^

^1^State Key Laboratory Incubation Base of Xinjiang Major Diseases Research, Clinical Medical Research Institute, First Affiliated Hospital of Xinjiang Medical University, Urumqi, Xinjiang, 830011, China

^2^Molecular Parasitology Laboratory, QIMR Berghofer Institute of Medical Research, Brisbane, Queensland, Australia

chinawenbaozhang2013@163.com

**Background:** Hydatid disease (HD) is a neglected zoonosis caused by *Echinococcus granulosus* (Eg), which distributes nearly around the world. The disease is hyper-endemic in western China with prevalence up to 12% of the population. Serological test for identification of cystic echinococcosis (CE) infection is still problematic in term of practical use in control program in large. Hydatid cyst fluid (HCF) antigens are the usual source of antigenic material for immunodiagnosis of CE. However, there are difficulties related to their lack of sensitivity (they miss about 30% of CE patients) for using these antigens to probe circulating antibodies in CE patients. We identified that *E. granulosus* antigen B (AgBs) were the genes with the highest gene expression in cyst stage by using transcriptome and proteomic analysis based on our recently finished genome analysis.

**Methods:** In the present study, we used an ELISA-based method to detect circulating antigens in 58 CE patients with type I–III cysts being serologically negative against HCF antigens in ELISA.

**Results and Conclusion:** We found more than 90% of these patients were circulating antigen-positive. Circulating antigen B positively existed in the sera of most of these antibody-negative patients, indicating that this antigen may be involved in regulation of B cells. Detection of both circulating antibodies and antigens may increase sensitivity and specificity of serological tests of cystic echinococcosis.

##### O-09. Comparative performance of the 2B2t recombinant antigen and hydatid fluid in ELISA and immunostrips for the diagnosis of cystic echinococcosis

Brunetti Enrico^1^, Mariconti Mara^1^, Meroni Valeria^1^, Delgado José Manuel^2^, Rojas José^2^, Santivañez Saul^3^, Hernández-González Ana^4^, Siles-Lucas Mar^5,^*

^1^Pavia University Hospital, Pavia, Italy

^2^Vircell SL, Granada, Spain

^3^INNPACE, Lima, Peru

^4^Instituto Carlos III, Madrid, Spain

^5^IRNASA, CSIC, Salamanca, Spain

mmar.siles@irnasa.csic.es

**Background:** The diagnosis of cystic echinococcosis (CE) is made by imaging methods but serology is generally used as a confirmatory test with the detection of specific antibodies. Hydatid fluid (HF) is currently the main source of antigens for this detection, but causes a number of false positive and negative reactions. In an attempt to overcome these drawbacks, several recombinant antigens have been produced and characterized. Among them, the 2B2t recombinant antigen has shown high specificity and sensitivity for the serodiagnosis of CE when used for the detection of specific IgG in ELISA (Hernández-González et al., 2010). We present the development and testing of a pre-commercial immunostrip containing the 2B2t antigen.

**Methods:** A total of 385 sera from CE patients, 86 sera from donors, 50 sera of alveolar echinococcosis (AE) patients and 104 sera of patients with neurocysticercosis (NCC) were tested. The HF and 2B2t antigens were used for the detection of specific IgG in ELISA and immunochromatography (strips) and their specificity and sensitivity compared.

**Results:** Overall sensitivity was of 86.8% and 91.5% for HF, and of 83.4% and 61.3% for the 2B2t in strips and ELISA format, respectively. For active cysts (CE1–CE3), sensitivity of strips containing either HF or 2B2t were comparable (~95%), with lower sensitivity for patients with CE1 cysts when the 2B2t strip was applied compared with the HF strip. For inactive cysts (CE4-5), 70.7% of patients tested positive with the HF strips, and only 59.4% were positive against the 2B2t antigen. Overall specificity was of 70.7% and 73.3% for HF and 2B2t strips, respectively. The main cross-reactivity found with HF was with AE patients and with NCC patients for the 2B2t antigen. Remarkably, cross-reaction of the 2B2t strips with NCC patients was mostly of very low intensity.

**Discussion/Conclusion:** Our results show that the 2B2t recombinant protein, a homogeneous and standardized antigen, could substitute HF in specific test formats maintaining sensitivity at comparable levels for active cysts and giving rise to a lower number of cross reactions with AE patients.

*The participation of MSL in this congress is partially funded by the European Community’s Seventh Framework Programme [FP7/2007-2013] under grant agreement n° HEALTH-F3-2013-602051-2 HERACLES

##### O-10. Correlation of serum sHLA-G levels with cyst stage in patients with cystic echinococcosis: an immune-evasion strategy?

Badulli Carla^1^, Mariconti Mara^1,4^, Tinelli Carmine^1^, Meroni Valeria^1,2^, Tamarozzi Francesca^2^, Genco Francesca^1^, Martinetti Miryam^1^, Brunetti Enrico^2,3^

^1^IRCCS, San Matteo Hospital Foundation, Pavia, Italy

^2^University of Pavia, Pavia, Italy

^3^WHO Collaborating Centre for Clinical Management of Cystic Echinococcosis, Pavia, Italy

c.badulli@smatteo.pv.it; f_tamarozzi@yahoo.com; enrico.brunetti@unipv.it

**Background:** Patients with cystic echinococcosis (CE) can harbour cysts for years or even decades, apparently without effect of the immune system on the metacestode. Although several immune evasion mechanisms by echinococcal cysts have been described, it is unclear whether the Human Leukocyte Antigen (HLA) system plays a role in the susceptibility or resistance to CE. HLA-G molecules are known to exert a suppressive action on Dendritic Cells maturation and on Natural Killer (NK) cells functions, therefore hampering T cell responses and NK cytolysis. HLA-G plays an important role in immune tolerance, is involved in foetus and in allotransplant tolerance, and may be involved in tumoral and viral immune evasion. In this study, we aimed to determine whether host’s soluble HLA-G (sHLA-G) may have a role in the course of human CE.

**Methods:** We analysed retrospectively 133 serum samples from 32 patients with hepatic CE. Sera were collected between June 2004 and September 2006 at the San Matteo Hospital Foundation, Pavia, Italy, and stored at −20 °C. For each patient, 3–4 serum samples obtained at different time points were available, before and after change in cyst stage. Sera from 38 healthy subjects were included as controls. For patients with more than 1 cyst, the one in active stage, according to the WHO classification, was considered for the analysis. All patients were diagnosed by ultrasonography and routine serology. Serum levels of total sHLA-G proteins, comprehensive of G1 and G5 isoforms, were measured using a commercial ELISA assay (sHLA-G ELISA kit, BioVendor, Praha, Czech Rep).

**Results:** i) sHLA-G levels in patients’ serum ranged from 0–86.5 ng/ml and no statistically significant difference was found between baseline values and those of controls (*p* = 0.61); ii) upregulation of sHLA-G correlated significantly with cyst activity (*p* = 0.003); iii) variations in sHLA-G levels were unrelated with patient’s sex.

**Discussion/Conclusion:** Our data suggest a role for HLA-G in the host-parasite interplay, with high or low levels of circulating sHLA-G seemingly correlating with presence of active/transitional or inactive cysts respectively.

##### O-11. Sensitive and specific immunohistochemical diagnosis of human alveolar echinococcosis with monoclonal antibody Em2G11

Gruener, Beate^1^, Barth Thomas F.E.^2*^, Herrmann Tobias S.^2*^, Tappe Dennis^3^, Stark Lorenz^2^, Buttenschoen Klaus^4^, Hillenbrand Andreas^5^, Juchems Markus^6^, Henne-Bruns Doris^5^, Kern Petra^7^, Seitz Hanns M.^8^, Moeller Peter^2^, Rausch Robert L.^9^, Kern Peter^1^, Deplazes Peter^10^

*Equally contributed to the work; published in: PLoS Negl Trop Dis. 2012;6(10):e1877

^1^Division of Infectious Diseases, Department of Internal Medicine III, University Hospital, Ulm, Germany

^2^Institute of Pathology, Ulm University, Ulm, Germany

^3^Bernhard-Nocht-Institute for Tropical Medicine, Hamburg, Germany

^4^Department of Surgery, Division of General Surgery, University of Alberta, Canada

^5^Department of General, Visceral and Transplantation Surgery, University Hospital, Ulm, Germany

^6^Department of Diagnostic and Interventional Radiology, University Hospital, Ulm, Germany

^7^Institute of Epidemiology and Medical Biometry, Ulm University, Ulm, Germany

^8^Institute of Medical Parasitology, University of Bonn, Bonn, Germany

^9^Department of Comparative Medicine, School of Medicine, University of Washington, Seattle, Washington, USA

^10^Institute of Parasitology, University of Zurich, Zurich, Switzerland

beate.gruener@uniklinik-ulm.de; thomas.barth@uniklinik-ulm.de

**Background:** Differential diagnosis of cystic echinococcsis (CE) caused by *E. granulosus* and alveolar echinococcosis caused by *E. multilocularis* (AE) is challenging. We aimed at improving diagnosis of AE on paraffin sections of infected human tissue by immunohistochemical testing of a specific antibody.

**Methods:** We have analysed 96 paraffin archived specimens, including 6 cutting needle biopsies and 3 fine needle aspirates, from patients with suspected AE or CE with the monoclonal antibody (mAb) Em2G11 specific for the Em2 antigen of *E. multilocularis* metacestodes.

**Results:** In human tissue, staining with mAb Em2G11 is highly specific for *E. multilocularis* metacestodes while no staining is detected in CE lesions. In addition, the antibody detects small particles of *E. multilocularis (spems)* of less than 1 μm outside the main lesion in necrotic tissue, liver sinusoids and lymphatic tissue most probably caused by shedding of parasitic material. The conventional histological diagnosis based on haematoxylin and eosin and PAS stainings were in accordance with the immunohistological diagnosis using mAb Em2G11 in 90 of 96 samples. In 6 samples conventional subtype diagnosis of echinococcosis had to be adjusted when revised by immunohistology with mAb Em2G11.

**Conclusion:** Immunohistochemistry with the mAb Em2G11 is a new, highly specific and sensitive diagnostic tool for AE. The staining of small particles *(spems)* outside the main lesion including immunocompetent tissue, such as lymph nodes, suggests a systemic effect on the host.

##### O-012. Cytokines and chemokines as predictive marker for cured, stable and progressive alveolar echinococcosis

Huang Xiangsheng^1^, Lechner Christian^1^, Gruener Beate^2^, Hoffmann Wolfgang^1^, Kern Peter^2^, Soboslay Peter^1^

^1^Institute for Tropical Medicine, University Clinics of Tübingen, Tübingen, Germany

^2^Division of Infectious Diseases and Clinical Immunology & Comprehensive Infectious Diseases Center, University Hospital, Ulm, Germany

peter.soboslay@uni-tuebingen.de

**Background:** In humans, *E. multilocularis* infection may remain asymptomatic for decades, the larval metacestode may persist in any organ, infiltrate other tissues and without adequate treatment its progressive growth may result in case fatality. Therefore, early detection of the metacestodes and appropriate intervention will result in better prognosis.

**Methods:** In the present works, we analysed *Echinoccoccus multilocularis*-specific cellular gene expression profiles in mononuclear peripheral blood cells (PBMC) from patients with cured and progressive Alveolar Echinococcosis (AE). Furthermore, PBMC were collected from AE patients (cured, stable, progressive) and infection-free controls, and were stimulated *in vitro* with *E. multilocularis* metacestode (Em) antigens. The cellular cytokine and chemokine productions by PBMC were quantified by ELISA aiming to identify distinctive immune response profiles in AE patient groups.

**Results:** Cellular gene (microarray) expression analyses in patients with progressive versus cured AE showed that the strongest inducible chemokine genes in healed AE were MCP4 > PARC > MPIF1. In patients with progressive AE, the chemokine genes for LARC > TARC > MCP3 were most highly expressed. The spontaneous cellular release of pro-inflammatory IL-31 and IL-33 was clearly depressed in all AE patients, while regulatory IL-27, anti-inflammatory SDF-1/CXCL12 and eosinophil granulocyte attracting Eotaxin-1, -2 and -3 (CCL11, CCL24, CCL26) were enhanced with disease progression. Such distinctive response profiles could be applied for monitoring of AE disease progression or regression. *E. multilocularis* metacestode (Em) antigens (entire metacestode EmAg as well as EmVesiclesAg) stimulated *in vitro* IL-31, IL-33, Eotaxin-1, -3 and CXCL12 cytokine and chemokine responses, which were similarly present in all AE patient groups, while regulatory IL-27 was suppressed and pro-inflammatory Eotaxin-2 was enhanced.

**Conclusion:** This study demonstrated dynamic profiles of the cellular immune responses during disease progression and regression, and these observations may help to improve monitoring and staging of AE.

##### O-13. May combined PET and serological follow-up predict a parasitocidal effect of chemotherapy in a subset of patients with non-resectable alveolar echinococcosis?

Ammann Rudolf W.^1^, Stumpe Katrin^2^, Grimm Felix^3^, Deplazes Peter^3^, Huber Sabine^1^, Bertogg-Seegers Kaja^1^, Fischer Dorothee R.^4^, Muellhaupt Beat^1^, the Swiss Echinococcosis Study Group (SESG)

^1^Department of Gastroenterology and Hepatology and Swiss Hepato-Pancreato-Biliary Center, University Hospital, Zürich, Switzerland

^2^Institute of Radiology, Hirslanden Hospital, Zürich, Switzerland

^3^ Institute of Parasitology, University of Zurich, Zürich, Switzerland

^4^Division of Nuclear Medicine, University Hospital, Zürich, Switzerland

beat.muellhaupt@usz.ch

**Background:** Benzimidazoles treatment has changed the natural history of non-resectable alveolar echinococcosis (AE). However it is commonly believed that they only have a parasitostatic effect and therefore long-term benzimidazole treatment is usually recommended. The aim of this study was to prospectively analyze the potential parasitocidal effect of benzimidazoles and whether normalization of FDG-PET-CT and anti-Emll/3-10-antibody levels are reliable parameters of AE-inactivation permitting abrogation of chemotherapy without risk for AE-recurrence.

**Method:** This study includes 34 patients with non-resectable AE subdivided into group A (*n* = 11) with newly diagnosed AE, (followed-up since diagnosis at month 6, 12 and 24 months and group B (*n* = 23) on long-term chemotherapy with a medium duration of 10 (2–25) years. Chemotherapy was stopped after normalization of FDG-PET-CT and serum anti-EmII/3-10 levels. A close follow-up for AE recurrence was performed. Endpoint (parasitocidal efficacy) was defined by absence of AE-recurrence >24 month after stopping treatment.

**Results:** Normalization of FDG-PET-CT scan and anti-EmII/3-10 titers occurred in 11 of 34 patients (32%). After stopping of chemotherapy no evidence of AE-recurrence was observed after a median of 70.5 (16–82) months.

**Conclusions:** Benzimidazole treatment was probably parasitocidal in one third of 34 patients of our series. Normal anti-EmII/3-10-level and no FDG uptake on PET-CT-scans were reliable parameters for assessing AE-larval viability in vivo.

#### Posters

##### P-10. Serological follow-up of alveolar echinococcosis in Japan using recombinant Em18: usefulness of a commercially available immunochromatography kit

Ito Akira^1^, Sako Yasuhito^1^, Akabane Hiromitsu^2^, Takahashi Masahiro^2^, Aoki Takanori^3^, Hagiwara Masahiro^3^, Ishikawa Yuji^4^, Yanagida Tetsuya^1^, Nakaya Kazuhiro^1^

^1^Asahikawa Medical University, Asahikawa, Japan

^2^Hokkaido Kouseiren Asahikawa-Kosei General Hospital, Asahikawa, Japan

^3^Hokkaido Kouseiren Engaru-Kousei General Hospital, Engaru, Japan

^4^Ishikawa Clinic, Asahikawa, Japan

akiraito@asahikawa-med.ac.jp

**Background:** In Japan, the first serological screening of alveolar echinococcosis (AE) in endemic area, Hokkaido, was established by Hokkaido Institute of Public Health (HIPH). The first and second screenings from 1987 until 2000 was ELISA and Western blot (WB) to detect 66, 55, 30–33 kDa bands using crude antigens of *E. multilocularis* metacestodes (EmCA). However, HIPH changed the diagnostic markers from higher to lower, 26–28, 18, 7–8 kDa bands from 2001. When HIPH introduced the new criteria and reexamined 1,745 stock samples which were pseudo-positive by EmCA-ELISA but negative by EmCA-WB (848 cases from screenings vs 897 cases from hospitals), 102 cases (5.8%) were confirmed to be AE: 81 AE cases were serologically confirmed (79.4%) whereas other 21 AE cases were accidentally confirmed to be AE through surgical treatment of other diseases (20.6%). Among 81 AE cases, 76 AE cases (93.8%) were 18 kDa (Em18) positive, whereas the rest 5 AE (6.2%) were Em18 negative but 26–28 kDa positive. Alternative diagnostic marker for AE in Japan has been Em18, especially using recombinant Em18 (rEm18) developed at Asahikawa Medical University (AMU) from 1999. As Em18 did not detect 100% of AE cases, HIPH does not introduce serology using rEm18, and still applies EmCA-ELISA and -WB which are time consuming and require special facilities, equipment, and technicians with charges of 1,400 (=14 US$) and 11,300 Jpn Yen (=113$), respectively. HIPH does not want to do blind test using rEm18 serology to compare or evaluate better and simpler tools. The Ministry of Education, Japan recommended AMU producing commercially available rapid immunochromatographic (ICT) kits for AE (ADAMU-AE), CE (ADAMU-CE) (2007–2011) and cysticercosis (ADAMU-CC) (2010–2012). ICT kits have been commercially available from March 2013 (ICST Co. Ltd., Saitama, Japan). In this study, we applied these serological tools using rEm18 for AE cases in Hokkaido and found interesting results.

**Methodology:** We conducted serology using rEm18 for detection of AE, follow-up studies of AE in Japan. REm18 serology could detect most of active AE cases and was highly useful for monitoring the progression of AE after treatment. Several hepatic AE cases with curative surgical resections showed unexpected rapid drop in antibody responses during the perioperative period. Most recently, we faced one early hepatic AE case which was expected to be metastasis of colon cancer treated several years before. This case was pseudo-positive by EmCA-ELISA but negative by EmCA-WB and also negative by rEm18 serology.

**Conclusion:** Our serological studies have revealed that rEm18 serology is highly useful for detection of the majority of active AE cases and for monitoring the progression. As ICT is simple and reliable, we do recommend its application.

##### P-11. Experimental whole blood test to diagnose and monitor cystic echinococcosis disease

Petrone Linda^1^, Vanini Valentina^1^, Petruccioli Elisa^1^, Ettorre Giuseppe Maria^2^, Busi-Rizzi Elisa^3^, Girardi Enrico^4^, Ludovisi Alessandra^5^, Pozio Edoardo^5^, Teggi Antonella^6^, Goletti Delia^1^

^1^Translational Research Unit Department of Epidemiology and Preclinical Research, “L. Spallanzani” National Institute for Infectious Diseases (INMI), Roma, Italy

^2^Unit of Surgery and Transplantation “Interaziendale” Department P.O.I.T., Polo Ospedaliero Interaziendale San Camillo-INMI Lazzaro Spallanzani, Roma, Italy

^3^Department of Radiology, “L. Spallanzani” National Institute for Infectious Diseases (INMI), Roma, Italy

^4^Department of Epidemiology and Preclinical Research, National Institute for Infectious Diseases (INMI), Roma, Italy

^5^Department of Infectious Parasitic and Immunomediated Diseases, Istituto Superiore di Sanità (ISS), Roma, Italy

^6^Department of Infectious and Tropical Diseases, Sant’Andrea Hospital University of Rome “Sapienza”, Roma, Italy.

delia.goletti@inmi.it

**Background:** The diagnosis and clinical management of human Cystic Echinococcosis (CE) is based on imaging examination and serology. However, currently used serological tests for CE diagnosis have some limitations as the high percentage of false-negative results and cross-reactions with other helminth infections. Therefore, improved diagnostic systems and identification of new biomarkers will provide powerful tools to defeat CE. Th2 response, play a crucial role in chronic helminthiasis. The aim of this study was to evaluate tools for improving CE diagnosis by analyzing the IL-4 response to Antigen B (AgB) of *Echinococcus granulosus* in a short- (1d) and long-term (3 and 5d) response using whole blood (WB) and peripheral blood mononuclear cells (PBMC).

**Methods:** We enrolled 37 CE patients with a confirmed diagnosis. IL-4 and IFN-γ response to AgB in PBMC and WB at day 1, 3 and 5 post-culture was evaluated by ELISA (high-sensitive for IL-4). Ten healthy donors (HD) were also included.

**Results:** The WB 1-day-stimulation was the best experimental condition for evaluating IL-4 in response to AgB. IL-4 level was significantly higher in CE patients than HD (*p* = 0.0001) whereas no difference regarding the IFN-γ response was found. Based on the significant difference, we performed a ROC analysis to evaluate IL-4-specific response potentials for CE diagnostics. Significant area under the curve (AUC) analysis results were obtained (AUC, 0.88; *p* = 0.0004). For scoring purposes we chose a cut-off point to maximize the sum of sensitivity and specificity: the cut-off point of 0.3 pg/ml predicted CE with 72.7% sensitivity and 90% specificity. Furthermore, we found that among the CE patients, IL-4 level was significantly increased in patients with active cysts compared to those with inactive cysts (*p* < 0.0001). Therefore we performed an additional ROC analysis and found significant AUC results (AUC, 0.91; *p* = 0.0003). We identified a cut-off point of 4.6 pg/mL which predicted active cysts diagnosis with 77.8% sensitivity and 91.3% specificity. Moreover, in two patients analyzed before and after surgery, IL-4-specific response decreased after cyst resection.

**Discussion/Conclusion:** Our preliminary data demonstrate that IL-4 specific response in WB after 1 day of specific stimulation is significantly associated with CE. Moreover, we show that IL-4 specific response higher than 4.6 pg/mL is associated with the presence of active cysts. Finally, IL-4 response seems to be a useful biomarker for CE monitoring in patients undergoing surgery for an active surveillance of CE relapse.

##### P-12. Expression of HIF-1α in the infiltrative belt surrounding hepatic alveolar echinococcosis in rat

Song Tao^1^, Li Haitao^2,3^, Wen Hao^2,3^

^1^Department of Ultrasonography, First Affiliated Hospital of Xinjiang Medical University, Urumqi, China

^2^Hepatobiliary & Hydatid Department, Digestive and Vascular Surgery Centre, First Affiliated Hospital of Xinjiang Medical University, Urumqi, China

^3^State Key Lab Incubation Base of Xinjiang Major Diseases Research (2010DS890294) and Xinjiang Key Laboratory of Echinococcosis, First Affiliated Hospital of Xinjiang Medical University, Urumqi, China

doctorsongtao@163.com

**Background:** To further investigate the expression of Hypoxia inducible factor -1 (HIF-1α) in the surrounding invasion range of hepatic alveolar echinococcosis (HAE) lesions and get the pathological basis of angiogenesis.

**Methods:** 23 Wistar rats with hepatic *Echinococcus multilocularis* infection were killed and then their livers were obtained which had 27 HAE lesions. The specimen segments were obtained from 119 paraffin blocks. Tissue samples containing the HAE nodules and the surrounding hepatic parenchyma were processed and then we proceeded to the comparative analysis using the immune-histo-chemical method. Expression of HIF-1α was compared in surrounding invasion range of the lesions and in the hepatic parenchyma.

**Results:** HIF-1α positive expression rate was 97.5% (116/119). The expression of HIF-1α in the active multiplying infiltrative region of the HAE lesion was also significantly higher than in the hepatic parenchyma (*P* < 0.05).

**Conclusions:** The overexpression of HIF-1α in the active multiplied infiltrative region of the HAE lesion of the rats is closely related with angiogenesis and microvasculature. HIF-1α is very sensitive and representative. It can indicate that the invasion range of HAE lesions was based on extrusion and compression and caused the hepatic tissue anoxic and ischemic. It might be a valuable index in evaluating activity of HAE.

##### P-13. New molecular diagnosis of polycystic echinococosis by *E. vogeli* in human and *Cuniculus paca* in South America

Vizcaychipi Katherina Alicia^1^, Naidich Ariel^1^, Noya-Alarcón Oscar^2^, Colmenares Cecilia^3^, Gutierrez Ariana^1^, Sanchez Pablo Omar^4^, Casas Natalia^5^, D’Alessandro Antonio^6^

^1^Departamento de Parasitología, INEI-ANLIS “Dr. Carlos G. Malbrán”, Buenos Aires, Argentina

^2^Unidad Ecoepidemiología, Centro Amazónico de Investigación y Control de Enfermedades Tropicales “Simón Bolívar”, Puerto Ayacucho, Estado Amazonas. Venezuela

^3^Sección de Inmunología, Instituto de Medicina Tropical, Universidad Central de Venezuela, Caracas, Venezuela

^4^Clínica y Maternidad Suizo Argentina (Swiss Medical SA), Buenos Aires, Argentina

^5^Programa Nacional de Control de Enfermedades Zoonóticas, Ministerio de Salud de la Nación, Argentina, Buenos Aires, Argentina

^6^Department of Tropical Medicine, Tulane University, New Orleans, LA, USA

kvizcaychipi@anlis.gov.ar

**Background and objective:** The polycystic echinococcosis by *Echinococcus vogeli* represents a severe medical problem in South America. Cyst *E. vogeli* is distinguished from other species of *Echinococcus* present in Central and South America based on the shape and size of the hooks of the protoscoleces rostellar. Our aim was to design a method for molecular diagnosis of *E. vogeli* in human and *Cuniculus paca* in South American samples to make a diagnosis possible when parasitology criteria are missing.

**Methods:** We worked with 8 samples of polycystic hydatid located in the liver of humans and *Cuniculus paca*. The origin of human samples were from Venezuela (*n* = 3) and Colombia (*n* = 1). *Cuniculus paca* samples were from Venezuela (*n* = 1), Argentina (*n* = 1) and Colombia (*n* = 2). All samples were positive for *E. vogeli* morphometrically.

The extraction of genomic DNA from protoscoleces, was performed by the automated method (Kit MagNA Pure Compact Nucleic Acid Isolation I- ROCHE). 2 sets of first (EV3 and EV5) were designed to amplify DNA of *E. vogeli* (fragments of 324 and 188 bp of the mitochondrial gene (cox1) based on the complete mitochondrial genome *E. vogeli* with samples of Colombia (GenBank accession number: AB208546), the specificity of these first was tested with DNA from *E. multilocularis*, *E. granulosus, E. vogeli*, *E. oligarthrus*, *Taenia hydatigena*, *Dipylidium caninum*, *Taenia saginata*, *Hymenolepis nana.* DNA extracted from each sample was amplified by MIT-PCR using primers designed for certain fragments of mitochondrial genes CO1.

**Results:** Preliminary results indicated that specific primer could be amplified EV3: *E. vogeli* (single band), *E. granulosus* (2 bands of 300 bp and a minor in 240 bp) and *E. oligarthrus* (single band with a size smaller than the fragment of *E. vogeli*) and amplified EV5: *E. vogeli* (single band) and *E. oligarthrus* (single band with a size smaller than *E. vogeli*). ItWe could identify the sequence by PCR of the samples from Venezuela (2 human, 1 *C. paca*). The sequences of the amplification of these isolates products showed 99% and 100% nucleotide identity to the reference sequence described for *E. vogeli*. We were unable to amplify and identify the sequence of the samples from Colombia and Argentina. This may be due to insufficient or degraded DNA.

**Discussion/Conclusion:** This is the first specific primer that would differentiate PCR samples of *E. vogeli* and *E. oligarthrus*, zoonotic species of importance to public health in South America. We are doing more studies to see if they can discriminate *E. vogeli* and *E. oligarthrus* depending on the length of the amplified fragments.

##### P-14. Assessment of a new immunochromatographic test for the diagnosis of cystic echinococcosis

Moreau Elise^1^, Zait Houria^2^, Grenouillet Florence^1^, Hamrioui Boussad^2^, Millon Laurence^1,3^, Grenouillet Frédéric^1,3^

^1^National Reference Centre for Alveolar Echinococcosis and WHO Collaborating Centre for Prevention and Treatment of Echinococcosis, Parasitology-Mycology Department, University Hospital, Besancon, France

^2^Parasitology-Mycology Department, Mustapha University Hospital, Alger, Algeria

^3^UMR CNRS-UFC 6249 Chrono-Environnement, University of Franche-Comté, Besancon, France

fgrenouillet@chu-besancon.fr

**Background:** Cystic echinococcosis (CE) is considered as neglected parasitic disease presenting high endemicity in several developping countries. Serological confirmation of CE diagnosis is often difficult to perform in these regions, regarding unavailability of close laboratories. In this context, immunochromatographic test (ICT) could be of potential interest for rapid CE diagnosis. Thus, we assess the performance of a newly commercialized ICT (Virapid^®^ Hydatidosis, Vircell, Spain), based on *E. granulosus* (*Eg*) 5/B antigen

**Patients & Methods:** Virapid^®^ Hydatidosis was assessed on two separate panels of sera. First retrospective panel included 224 sera of patients with well-documented disease: probable/proven CE (94/225; 42.0%), probable/proven alveolar echinococcosis AE (25/224), others parasitic diseases (43/224), non-parasitic liver and/or autoimmune diseases (62/224). Second prospective panel included 115 sera, analyzed in our lab for *Echinococcus* serology (primary diagnosis only) during a 4-month period (CE : 3/115, 2.6%). All sera were also analyzed using our standard serological scheme (Indirect hemagglutination Fumouze, Levallois-Perret, France; *Eg* and Em2^+^ Elisa Bordier, Crissier, Switzerland; Western Blot *Echinococcus* LDBioDiagnostic, France). Sensitivity (Se), specificity (Sp), positive and negative likehood ratio (LR+, LR−) were determined for all test considering two potential diagnosis: CE, or “Echinococcosis” (i.e AE or CE).

**Results:** Considering equivocal test as negative, assessment of retrospective sera showed following ICT performances for diagnosis of echinococcosis: Se 0.782, Sp 0.875, LR + 6.31, LR − 0.25. For specific diagnosis of CE, ICT test showed decreased Sp and LR+, 0.746 and 3.05 respectively. 20 out of 25 AE sera were positive using Virapid ICT^®^. False positive ICT results were observed with cysticercosis (*n* = 2), fasciolosis (*n* = 4), cirrhosis (*n* = 4), and polycystic liver disease (Caroli disease, *n* = 2). Assessment of prospective panel led to higher Se but lower Sp (Se 0.840; Sp 0.743; LR + 3,268; LR − 0,215 considering diagnosis of echinococcosis).

**Conclusion:** Virapid^®^ Hydatidosis is an interesting tool for easy and rapid CE diagnosis, using two separate panel with significative different prevalence of CE (42.0% and 2.6%). Cross-reactivity with AE limits its use for specific diagnosis of CE to AE free-area. However, this cross-reactivity could be an asset for the use of ICT as rapid screening test in AE and CE co-endemicity area. Complementary field assessment in highly endemic country, compared to ultrasound screening, could be of interest.

##### P-15. External quality assessment for *Echinococcus* serology: a French initiative

Roussel Sandrine^1,2^, Grenouillet Florence^1^, Demonmerot Florent^1^, Scherer-Didier Emeline^1,2^, Millon Laurence^1,2^, Grenouillet Frédéric^1,2^

^1^National Reference Centre for Alveolar Echinococcosis and WHO Collaborating Centre for Prevention and Treatment of Echinococcosis, Parasitology-Mycology Department, University Hospital, Besancon, France

^2^UMR CNRS-UFC 6249 Chrono-Environnement, University of Franche-Comté, Besancon, France

fgrenouillet@chu-besancon.fr

**Background:** Accreditation to ISO 15189 or to others international standards (i.e. ISO/IEC 17025 and ISO 9001) is an essential step for medical laboratories to demonstrate the quality and competence of their services. It addresses especially the qualifications and on-going competency of personnel, pre-analytical and analytical factors, quality assurance considerations, and post-analytical factors. It includes requirement of participation to external quality assessment (EQA) or Inter Laboratory Comparison (ILC) program for each parameter. UKNEQAS organize EQA program for cystic echinococcosis (CE) serology. As no EQA program was available for alveolar echinococcosis (AE) serology, the French National Reference Centre for Alveolar Echinococcosis (NRC) implemented such EQA program in 2013 at national level, and renewed it in 2014 at European level.

**Patients & Methods:** For first EQA program (2013), ten French laboratories were directly solicited for participation to this program. Choice of solicited labs was based either on their high-level activities (annual number of serologies performed) or on share of Elisa techniques with the French NRC. Two anonymous sera were sent for expertise to participating labs in March 2013 (one from AE patient, one from CE patient). Inter-laboratory variability of Elisa results was assessed using these two sera. For renewal of this EQA program in 2014, large proposal to all French laboratories and to several European laboratories was done.

**Results:** 2013 EQA program revealed high inter-laboratory variability with commercialized Elisa techniques shared by participants (i.e. *E.g* and Em2^+^ Elisa, Bordier, Switzerland) with CV values equal to 0.50 (for Em2^+^) and 0.25 (for *E.g*). Similar levels of intra-laboratory variability were observed in French NRC. 3 laboratories failed to identify positive CE serology (sera showing low reactivity using screening tests).

2014 EQA Program will include two sets of two sera. First set was sent to 21 French and 5 European labs in January 2014 (second set in September 2014). Results of this EQA 2014 will be presented

**Discussion:** This work underlines the major interest of inter-laboratories controls for each participating lab in knowledge of its own laboratory techniques. This EQA program, financially supported by French NRC, let to an increased confidence level of analytical results, and to the implementation of a European network of labs involved in echinococcosis diagnosis.

## Session 2. New tools for epidemiology and prevention

### 

#### State-of-the-art

##### L-04. New tools for new challenges: *Echinococcus* epidemiology on the move

Romig Thomas

Universität Hohenheim, Stuttgart, Germany

Thomas.Romig@uni-hohenheim.de

Epidemiology is a catch-all term for a wide range of aspects. Consequently, lots of different tools are in use, ranging from diagnostic methods through parasite characterization techniques to various models for risk assessment, spatial/temporal occurrence and parasite development. Methods for molecular characterization of parasite isolates are currently in focus to address new questions. For example, presence and frequency of *E. multilocularis* are now well established for the largest parts of Europe and eastern Asia, and new records are emerging even from countries with few previous data (e.g. North America and central Asia). Consequently, the research foci have now shifted towards issues like parasite spread or invasion/introduction into non-endemic areas, or the quantitative role of individual host species in transmission. Various genetic markers have been successfully applied to this, e.g. microsatellite profiles (France, Italy, Svalbard), or mitochondrial sequence markers (Asia, North America). The situation is far more complex in case of cystic echinococcosis (*E. granulosus* sensu lato). The various species have been shown to differ in epidemiologically relevant characters, but only very recent studies have taken this into account. This means that much epidemiological and geographical information needs rebuilding using specific diagnostic tools. Various mitochondrial markers (from short sequences to complete mt genomes) have been used, some for >20 years, but there are recent additions to the toolbox, e.g. a LAMP PCR system that allows specific diagnosis without expensive equipment. Intraspecific microvariation (e.g. mitochondrial haplotypes) has proven useful to address questions of biogeography. Geographical differences in haplotype diversity of *E. granulosus* sensu stricto has led to a hypothesis of Western Asia being the region of origin for this taxon, and demonstrated founder effects in China and South America. Haplotype networks are currently being used to study possible routes of introduction and expansion in sub-Saharan Africa, and to analyse interactions between domestic and silvatic transmission cycles. A challenging task for field workers has always been the specific identification of infection status and host species of faecal samples collected in the environment, which is the method of choice to study the role of domestic or wild carnivores. Such samples are often degraded, and single cestode eggs have to be analyzed to avoid mixing of different taxa. New techniques may in future overcome some of the problems. Molecular determination of the host species of such samples is equally important, as identification by field signs is often unreliable. A number of PCR-based techniques are now available for this purpose. As rather neglected field were imaging techniques for animals, where new generation ultrasound seems now to become a suitable tool to monitor CE infection in livestock under field conditions, e.g. in the context of control trials or vaccine studies.

**References**

Davidson RK, Romig T, Jenkins E, Tryland M, Robertson LJ. The impact of globalisation on the distribution of *Echinococcus multilocularis*. Trends Parasitol. 2012 Jun;28(6):239–47.

Dinkel A, Kern S, Brinker A, Oehme R, Vaniscotte A, Giraudoux P, Mackenstedt U, Romig T. A real-time multiplex-nested PCR system for coprological diagnosis of *Echinococcus multilocularis* and host species. Parasitol Res. 2011 Aug;109(2):493–8.

Jenkins EJ, Peregrine AS, Hill JE, Somers C, Gesy K, Barnes B, Gottstein B, Polley L. Detection of European strain of Echinococcus multilocularis in North America. Emerg Infect Dis. 2012 Jun;18(6):1010–2.

Knapp J, Bart JM, Maillard S, Gottstein B, Piarroux R. The genomic *Echinococcus* microsatellite EmsB sequences: from a molecular marker to the epidemiological tool. Parasitology. 2010 Mar;137(3):439–49.

Knapp J, Nakao M, Yanagida T, Okamoto M, Saarma U, Lavikainen A, Ito A. Phylogenetic relationships within *Echinococcus* and Taenia tapeworms (Cestoda: Taeniidae): an inference from nuclear protein-coding genes. Mol Phylogenet Evol. 2011 Dec;61(3):628–38.

Knapp J, Staebler S, Bart JM, Stien A, Yoccoz NG, Drögemüller C, Gottstein B, Deplazes P. *Echinococcus multilocularis* in Svalbard, Norway: microsatellite genotyping to investigate the origin of a highly focal contamination. Infect Genet Evol. 2012 Aug;12(6):1270–4.

Mogoye BK, Menezes CN, Wong ML, Stacey S, von Delft D, Wahlers K, Wassermann M, Romig T, Kern P, Grobusch MP, Frean J. First insights into species and genotypes of *Echinococcus* in South Africa. Vet Parasitol. 2013 Sep 23;196(3–4):427–32.

Nakao M, Li T, Han X, Ma X, Xiao N, Qiu J, Wang H, Yanagida T, Mamuti W, Wen H, Moro PL, Giraudoux P, Craig PS, Ito A. Genetic polymorphisms of *Echinococcus* tapeworms in China as determined by mitochondrial and nuclear DNA sequences. Int J Parasitol. 2010 Mar 1;40(3):379 85.

Rojas CA, Romig T, Lightowlers MW. *Echinococcus granulosus sensu lato* genotypes infecting humans-review of current knowledge. Int J Parasitol. 2014 Jan;44(1):9–18.

Romig T, Omer RA, Zeyhle E, Hüttner M, Dinkel A, Siefert L, Elmahdi IE, Magambo J, Ocaido M, Menezes CN, Ahmed ME, Mbae C, Grobusch MP, Kern P. Echinococcosis in sub-Saharan Africa: emerging complexity. Vet Parasitol. 2011 Sep 8;181(1):43–7.

Romig T. *Echinococcus multilocularis* in Europe-state of the art. Vet Res Commun. 2009 Sep;33 Suppl 1:31–4.

Wahlers K, Menezes CN, Wong ML, Zeyhle E, Ahmed ME, Ocaido M, Stijnis C, Romig T, Kern P, Grobusch MP. Cystic echinococcosis in sub-Saharan Africa. Lancet Infect Dis. 2012 Nov;12(11):871–80.

Wassermann M, Mackenstedt U, Romig T. A loop-mediated isothermal amplification (LAMP) method for the identification of species within the *Echinococcus granulosus* complex. Vet Parasitol. 2014 Feb 24;200(1–2):97–103.

#### Oral communications

##### O-14. GP/EFSA/AHAW/2012/01: *Echinococcus multilocularis* infection in animals

Casulli Adriano, Pozio Edoardo, for the European consortium

Istituto Superiore di Sanità, Rome, Italy

adriano.casulli@iss.it

In recent years the presence of EM has been reported from areas of Europe in which it previously had not been recognised. At the same time, increases of EM prevalence in foxes have been observed in several European countries. In addition, urban fox populations have become established in many central European cities, reaching high population densities and sometimes also high prevalence of EM infections, increasing the risk of transmission to humans.

Till now, four EU Member States (Finland, Ireland, Malta and the UK) have submitted documentation supporting the evidence of the absence of the parasite. Regulation (EU) No 1152/2011 provides that a pre-movement anti-parasite treatment has to be applied to dogs entering these countries and that a pathogen-specific surveillance programme, adhering to certain requirements regarding sampling and detection techniques has to be operated by these countries. The Commission has to review Regulation (EU) No 1152/2011 no later than December 2016 in the light of scientific developments regarding EM infection in animals. To assist in this review, EFSA founded the project “*Echinococcus multilocularis* infection in animals” to provide a scientific opinion on EM infections in animals by the end of 2015. In order to be able to provide a comprehensive and quantitative assessment of EM infections in animals the current knowledge and data on the epidemiology and risk factors related to this disease will be collected in the European Union and adjacent countries. Information and data on the aspects listed above will be gathered by means of systematic reviews of literature and data.

The project “*Echinococcus multilocularis* infection in animals” is funded by EFSA. Duration of the project: 2013–2015. The Consortium is composed by the Applicant (ISS, Italy), the Partners (ANSES, France; EVIRA, Finland; RIVM, Netherlands; FLI, Germany; NVRI, Poland; CSIC, Spain), the Systematic Review Advisor (Sapienza University, Rome) and External Experts.

##### O-15. HERACLES (Human Cystic Echinococcosis Research in Central Eastern Societies)

Casulli Adriano^1^, Pozio Edoardo^1^, for the HERACLES European consortium

Istituto Superiore di Sanità, Rome, Italy

adriano.casulli@iss.it

Cystic echinococcosis (CE), one of the most widespread helminthic zoonosis, is a chronic disease caused by infection with larval stage of the tapeworm *Echinococcus granulosus* complex (Egc). The diagnosis of human CE is based on clinical findings, imaging techniques and serology. Decision making for treatments is difficult depending on the fact that the natural evolution of the cyst is basically unknown. De facto CE is chronic, complex and still neglected.

HERACLES project is designed to provide new insights into parasite/host relationship associated with the epidemiology, clinical manifestation, parasite infectivity, host immunity, improvement of therapeutic treatment and new tools for the detection, diagnosis and follow-up of CE. HERACLE will translate the research results into cheap and easy-to-use, point-of-care lab on a chip (POC-LOC) commercial tools for its use in less favored Central and Eastern Europe (CEE) countries affected by CE. The final project stakeholders, such as the rural populations, in which CE is endemic, as well as Small-medium enterprises (SMEs) will be engaged as an integral part of the work.

The key objectives of HERACLES are:

- Identify the population affected by CE in endemic rural areas of CEE countries;
- Create CEE national registries;
- Establish a representative collection of genetic Egc isolates and blood/serum/plasma samples;
- Validate new molecular-based POC-LOC kits;
- Identify factor/s associated with CE response to therapy or lack thereof through host-parasite interplay;
- Increase drug bioavailability in an in vivo model and to synthesize a new enantiomeric drug based on ABZ.

HERACLES project (GA 602051) was funded under the 7th Framework Program of EU (HEALTH.2013.2.3.4-1: Neglected infectious diseases of Central and Eastern Europe).

The duration of the project is 2013–2017. The Consortium is composed by the Applicant (ISS, Italy) and the following Partners from European universities, research institutes, hospitals and SMEs (UNIPV, Italy; CSIC, Spain; CCHUMFCD, Romania; HUSM, Turkey; SHATIPD, Bulgaria; PVCI, Turkey; ALTA, Italy; Vircell, Spain).

##### O-16. Antibody responses to recombinant antigen B8/1 in cystic echinococcosis, Mongolia, based on molecular identification of the genotypes or species

Ito Akira^1^, Dorjsuren Temuulen^2^, Davaasuren Anu^3^, Sako Yasuhito^1^, Yanagida Tetsuya^1^, Bat-Ochir Oyun-Erdene^4^, Ayushkhuu Tsendjav^5^, Gonchigsengee Nyamkhuu^6^, Agvaandaram Gurbadam^2^, Davaajav Abmed^3^

^1^Asahikawa Medical University, Asahikawa, Japan

^2^Health Sciences University of Mongolia (HSUM), Ulaanbaatar, Mongolia

^3^National Center for Communicable Diseases (NCCD), Ulaanbaatar, Mongolia

^4^National Center of Pathology (NCP), Ulaanbaatar, Mongolia

^5^National Center for Maternal and Child Health (NCMCH), Ulaanbaatar, Mongolia

^6^State Central Clinical Hospital (SCCH), Ulaanbaatar, Mongolia

akiraito@asahikawa-med.ac.jp; yanagida@asahikawa-med.ac.jp

**Background:** *Echinococcus granulosus* sensu stricto (s.s.) cycles between dog and sheep and is the major causative agent of Cystic echinococcosis (CE). However, recent molecular epidemiological studies of CE cases have revealed that *Echinococcus canadensis* is also distributed worldwide. CE cases caused by *E. canadensis* have been reported not to be rare and even rather common where camels are present. As one of such countries where camels are present is Mongolia, we identified the causative species of human CE and analyzed antibody responses.

**Method:** We conducted molecular identification of 43 CE pathological specimens surgically removed at SCCH and NCMCH from Jan 2009 until Feb 2011 in Ulaanbaatar, Mongolia using mitochondrial cytochrome *c* oxidase subunit 1 (*cox1*) gene, and applied ELISA using recombinant Antigen B8/1 (rAgB).

**Results:** Molecular analysis of *cox1* gene revealed the etiological agents of CE cases as *E. canadensis* (*n* = 31, 72.1%) [G6/7 (*n* = 29) and G10 (*n* = 2)] and *E. granulosus* s.s. (G1) (*n* = 12, 27.9%). The majority of *E. canadensis* was G6/7 (29/31, 93.5%). Serum samples were available from 31 (20 and 1 of G6/7 and G10, respectively, and 10 of G1) of 43 CE samples confirmed by molecular tools. Antibody responses to rAgB were positive in 9/10 (90%) and 13/20 (65%) of G1 and G6/7, respectively, (Wilcoxon rank run test, *p* = 0.2103), and absorbance values of rAgB-ELISA were statistically higher in CE cases caused by *E. granulosus* s.s. (G1) than those by *E. canadensis* (G6/7) (Fisher’s exact test, *p* = 0.0137). Average age of CE cases was much higher by *E. granulosus* s.s. (G1) (47.6) than by *E. canadensis* (23.3).

**Discussion:** The present study shows the majority of CE cases are caused by *E. canadensis* (G6/7) but not *E. granulosus* s.s. (G1) and differs from Jabbar et al. (2011). It may be due to the difference in the patients’ living areas, since CE patients were from central to western parts of Mongolia in this study, whereas those were from eastern to central in Jabbar et al. (2011). As the definitive host of *E. canadensis* is *Canis lupus* (Ito et al. 2013) and that of *E. granulosus* s.s. should be *Canis canis* (no data), the crucial difference in the definitive host species and their living environment may have some influence on the average age of CE caused by these two species.

**Conclusion:** This is the first report to show antibody response to rAgB in *E. canadensis* cases. As CE confirmed from young generation may be caused by *E. canadensis* (G6/7) more than by *E. granulosus* s.s. (G1), we have to confirm the causative species for epidemiological studies of CE.

##### O-17. The transcriptome of adult *Echinococcus granulosus* and dog vaccination

Zhang Wenbao^1^, Li Jun^1^, Zhang Zhuangzhi^2^, Shi Baoxin^2^, Zhen Huajun^3^, Zhou Yan^3^, Wang Shengyue^3^, McManus Donald P.^4^, Wen Hao^1^

^1^Clinical Medical Research Institute, First Affiliated Hospital of Xinjiang Medical University, Urumqi, China

^2^Veterinary Research Institute, Xinjiang Academy of Animal Sciences, Urumqi, China

^3^Shanghai-Ministry of Science and Technology Key Laboratory of Health and Disease Genomics & Chinese National Human Genome Center at Shanghai, Shanghai, China

^4^Molecular Parasitology Laboratory, QIMR Berghofer Institute of Medical Research, Brisbane, Queensland, Australia

wenbaozhang2013@163.com

**Background:** Despite the substantial efforts that have been made to control echinococcosis there is a clear need for new tools in its prevention. Dogs are pivotal in *Echinococcus granulosus* transmission and we contend that interruption of the parasite life cycle in the definitive host stage provides a very practical and cost-effective vaccination strategy. Identification of vaccine candidates is a crucial step for vaccine development.

**Methods:** Adult *E. granulosus* worms aged two weeks were obtained from experimentally infected dogs. The worms were fixed in a worm fix buffer and stored in ethanol. Each of the worms was cut into two parts, head and neck region. mRNA were extracted from the two parts respectively and RNA-seq technique was used to precisely quantify transcript levels in the two parts. To identify genes specifically expressed in the rostellum, a laser microdissection microscope was used to isolate rostellum and neck tissues. A real time PCR was used to confirm the gene expression in the tissues.

**Results:** We identified 953 genes specifically or differentially expressed in the rostellum and sucker region. Most of the differential genes are novel genes, indicating these genes may compose a network in regulating specific function of worm head, and they may play an important role in settlement of *E. granulosus* in dog intestinal surface. We have identified several genes specifically expressed in the rostellum of *E. granulosus*. We have expressed several genes for evaluating their vaccine efficacy for dogs against *E. granulosus* infection.

**Discussion/Conclusion:** Our previous vaccination trials showed that dogs vaccinated with *E. granulosus* adult specific proteins EgM9 and EgM123 emulsified with Freund’s and Quil A adjuvants induced a significant protection efficacy in terms of reduction of worm burden and eggs at day 45 post-infection. However, our recent trial for the longevity of protection showed that dogs vaccinated with three doses of EgM123 produced discontinuously a small amount of eggs after 50 days post-infection, which indicates that a single subunit vaccine is difficult to induce a complete and long term of projection. To search more vaccine candidates, we isolated a range of genes expressed in the rostellum of the worms, which are likely to be vaccine candidates.

##### O-18. Genetic diversity of *Echinococcus multilocularis* – comparative results from mitochondrial and microsatellite markers

Schroer Sandra^1^, Knapp Jenny^2^, Gottstein Bruno^3^, Dinkel Anke^1^, Romig Thomas^1^

^1^Parasitology Unit, University of Hohenheim, 70599 Stuttgart, Germany

^2^Department of Parasitology, UMR CNRS-UFC 6249 Chrono-Environnement, University of Franche-Comté, Besancon, France

^3^Institute of Parasitology, University of Bern, CH-3012 Bern, Switzerland

sandra.schroer@uni-hohenheim.de

**Background:** By analysis of mitochondrial marker genes, major clades within *E. multilocularis* have previously been identified which are partly correlated with geographical regions (e.g. “Asian” vs. “European” strains). However, descriptions of diversity within smaller geographical units were mainly done by comparing profiles of microsatellite markers (EmsB). To evaluate the use of mt markers for this purpose, we compared both systems for their discriminative potential.

**Methods:** We investigated 350 isolates of adult worms from various European countries (Switzerland, Germany, Austria, Poland, The Netherlands and Slovakia) by partial sequencing of the mitochondrial genes cox1, nd1 and atp6 (~1600 concatenated bp’s) and by analysis of EmsB profiles.

**Results:** In total, our samples contained 30 different EmsB profiles, with the highest diversity in Switzerland and Germany, whereas the number of profiles decreased towards the periphery of the endemic area. Across the three mt genes, 50 haplotypes were identified, most of them no yet recorded before. No significant difference of variation was found between the three genes. Again, the highest number of mt haplotypes was found in Switzerland, Germany and Austria. No obvious correlation was found between individual EmsB profiles and certain mt haplotypes, although some “pairs” seemed to be associated. Distribution of both ms profiles and mt haplotypes are highly uneven across Europe, but few variants occur only in one country.

**Conclusion:** Very similar diversity patterns of *E. multilocularis* in Europe were identified with both – fundamentally different – systems, which adds a new tool for epidemiological application on smaller scales.

##### O-19. Development of Loop-Mediated Isothermal Amplification (LAMP) assay for the differentiation of sub-Saharan African *Echinococcus* species

Wassermann Marion^1^, Mackenstedt Ute^1^, Romig Thomas^1^

^1^Dept. of Parasitology, University of Hohenheim, Stuttgart, Germany

huettner@uni-hohenheim.de

**Background:** Cystic echinococcosis is most prevalent in resource-poor regions where the identification of CE agents is restricted by the lack of sophisticated laboratory equipment. The precise identification, however, is a substantial requirement for understanding the epidemiology of the disease.

**Methods:** To facilitate the specific identification of *Echinococcus* spp. isolates, five LAMP (loop-mediated isothermal amplification) assays were developed to detect the various agents known to cause cystic echinococcosis (*E. granulosus* s.s., *E. equinus*, *E. ortleppi*, *E. canadensis* and *E. felidis*).

**Results:** All assays were strictly species specific. The diagnostic power was adjusted to species level, i.e. intraspecific strains (G1-3 within *E. granulosus* s.s., G6-10 within *E. canadensis*) are not discriminated. The sensitivity of all assays was tested down to one fiftieth of a single protoscolex or egg, respectively.

**Discussion/Conclusion:** The infectivity of the different species and the severity of the disease in humans and livestock vary significantly among those species, and correct molecular identification of large numbers of field isolates is crucial to understand their epidemiology. However, funding constraints in many CE endemic countries often prevent PCR-based screening of field isolates. The LAMP method can be employed in laboratories with basic equipment, as thermocyclers and electrophoresis are not necessary. The method allows the amplification of DNA fragments under isothermal conditions which can be achieved using an ordinary water bath, and the detection of amplification products only requires a UV light source. In the present study five different LAMP assays were developed. Wherever this would be necessary for epidemiological purposes, the method can be adjusted to include other *Echinococcus* species or discriminate major intraspecific genotypes.

##### O-20. Development and validation of a multiplex PCR for simultaneous detection and genotyping of the *E. granulosus* complex

Boubaker Ghalia, Spiliotis Markus, Gottstein Bruno

Institute of Parasitology, University of Bern, Bern, Switzerland

markus.spiliotis@vetsuisse.unibe.ch

**Background:** *Echinococcus granulosus* exhibits substantial genetic diversity; ten genotypes have been documented (G1–G10). According to the new molecular phylogeny of the genus *Echinococcus*, the *E. granulosus* complex has been divided into *E. granulosus* sensu stricto (G1–G3), *E. canadensis* (G8/G10), while *E. intermedius* (G6/G7) is still a debatable issue. The two remaining strains G4 and G5 have been elevated to a species status, respectively *E. equinus* and *E. ortleppi*. Up to now, existing molecular genotyping assays for identification of different strains are time consuming and/or do not comprise all *E. granulosus* complex genotypes. Here we describe a standard multiplex PCR-based assay for rapid simultaneous identification and discrimination between all *E. granulosus* genetic variants.

**Methods:** In total, twenty two primers were included to amplify eleven different DNA fragments; we used two sets of primers per genotype, except for G8/G10 (one couple of primers). Additionally, two sets of primers specific for highly conserved regions were designed to cover respectively all *E. granulosus* complex (G1–G10) and all known species within the *Echinococcus* genus (*E. granulosus*, *E. multilocularis*, *E. vogeli*, *E. oligarthrus* and *E. shiquicus*). Thus, for each sample, three levels of discrimination are yielded: the genus, the species and the strain/genotype. Target sequences for amplification are parts of nuclear and mitochondrial genes. This combination allowed us to find specific polymorphic sites for different but genetically closely related strains. The multiplex-PCR was then validated using of 267 samples from 6 countries (Tunisia, Algeria, Sudan, Spain, Bulgaria and Argentina).

**Results:** Specificity of the multiplex PCR was 100% when evaluated with isolates of different 7 species of cestodes. Sensitivity threshold was 5 ng of DNA per sample. We validated accuracy of our approach by comparing with *coxI* sequencing results. Additionally, the multiplex PCR has the advantage of detecting multiple genotype infections, and in avoiding DNA isolation steps, by using only, fresh or frozen, partially treated parasite materials (protoscoleces, hydatid fluid). The Multiplex PCR test applied on 267 samples collected from different geographical regions, including three continents demonstrated a higher presence of the major *E. granulosus* species, *E. granulosus* sensu stricto (G1–G3) and *E. intermedius* (G6/G7).

**Discussion/Conclusion:** Except for copro analysis, the multiplex-PCR described here has a high potential for a worldwide application in large-scale molecular epidemiological studies on the *Echinococcus* genus.

##### O-21. Using the genetics of *Echinococcus multilocularis* to trace the history of expansion from an endemic area

Umhang Gérald^1^, Knapp Jenny^2^, Hormaz Vanessa^1^, Raoul Francis, Boué Franck^1^

^1^Wildlife eco-epidemiology and surveillance unit, National Reference laboratory for *Echinococcus* spp., ANSES Nancy laboratory for rabies and wildlife, 54220 Malzéville, France

^2^UMR UFC/CNRS 6249 usc INRA ‘CHRONO-ENVIRONMENT’, University of Franche-Comte, 25030 Besancon, France

gerald.umhang@anses.fr

**Background:** Alveolar echinococcosis, caused by the cestode *Echinococcus multilocularis*, is the most serious parasitic disease for humans in Europe, with a sylvatic life cycle generally between small rodents and red foxes. General expansion of the range of *E. multilocularis* has been observed across Europe over the last fifteen years. In France, a westward spread of the parasite was described recently.

**Methods:** For genotyping, the microsatellite EmsB was used to trace expansion in five French areas.

**Results:** A total of 22 EmsB profiles were identified, with five similar to those previously described in Europe. An imbalance of genetic diversity was observed between the five areas which also revealed their interconnection with the presence of common profiles, notably the main two profiles both present in all regions except one, where only one was found.

**Discussion/Conclusion:** These two findings are similar to those described at the European level, highlighting transmission of the parasite by a mainland-island system. A spatio-temporal scenario of the expansion of *E. multilocularis* can be proposed with spread from the French historical focus in eastern France to the Lorraine, the Champagne-Ardenne and finally the North, while simultaneously another expansion has occurred from the historical focus into an area of north-west France. The colonization by the parasite into the West and North areas from the historical focus was due to the migration of foxes several decades ago. Recent detection of the parasite in new endemic “départements” may be due to more active research and more accurate diagnoses rather than a recent spread of the parasite, highlighting the need for monitoring of *E. multilocularis* throughout the country. Regarding the numerous data obtained by the different EmsB analyses across Europe, centralization of all the profiles described appears necessary in order to obtain a precise understanding of transmission of the parasite from one country to another.

##### O-22. Occurrence of *Echinococcus multilocularis* eggs in environment in endemic region of Poland using molecular techniques*

Szostakowska Beata, Lass Anna, Pietkiewicz Halina, Nahorski Wacław L., Sulima Małgorzata, Kostyra Katarzyna, Hallmann Sylwia, Abramowska Anna, Cejrowska Natalia, Myjak Przemysław

Medical University of Gdańsk, Gdańsk, Poland

bszost@gumed.edu.pl

**Background:** *Echinococcus multilocularis* is the etiological agent of alveolar echinococcosis (AE), potentially deadly zoonosis occurring in northern hemisphere. According to recent studies, the number of human AE cases increases and endemic area of the tapeworm is larger than previously known. Human becomes an accidental intermediate host after ingestion of tapeworm’s eggs present in environment. Despite this, little is known about the level of environment contamination with the parasite eggs, and thereby, the risk of being infected by contact with different environmental samples.

**Methods:** The environmental samples: soil (*n* = 62), plants (*n* = 103), water (*n* = 21) and air (*n* = 11) from an endemic region of northern Poland were analyzed, to establish the exposure of people to risk of infection with *E. multilocularis*. Samples were collected in natural environment and around homesteads, where foxes were often observed. Each kind of samples was treated in different way to recover eggs of parasites. Molecular investigations were performed to detect DNA of parasite in samples. The fragment of mitochondrial 12S*rrna* gene was examined using nested PCR.

Simultaneously with environmental studies, 410 inhabitants were subjected to medical tests (imaging examinations, serological tests) to establish correlation between the level of environment contamination and new AE cases.

**Results:** DNA of *E. multilocularis* was detected in all kinds of samples. The biggest number of positive reactions was obtained in air samples (36.4%), then in plants (29.1%), water (14.3%) and soil (11.3%). Results demonstrated that environment, both natural and homesteads’ surroundings can be contaminated with eggs of *E. multilocularis* and it can pose direct threat to human’s health. Medical survey did not reveal new AE cases, however, one person with early, asymptomatic stage of cystic echinococcosis was diagnosed using imaging examination, then confirmed by serological tests.

**Discussion and Conclusions:** Efficiency of recovery methods from the environmental samples (especially soil and plants) is unsatisfactory. Moreover, eggs of this parasite are dispersed in the environment. For these reasons, it is highly probable that contamination of environment can be higher than we were able to prove.

Medical tests did not reveal new AE cases. Maybe the persons subjected to medical examinations (not specifically chosen) were not the most exposed to infection.

Environment of endemic areas should be regularly monitored and preventive measures should be introduced (informative campaigns and limitation of the tapeworm eggs spreading in environment) to protect people, especially living in these regions.

*Study supported by Research Grant No. N N402 587140 from the State Committee for Scientific Research, Poland.

##### O-23. Italian Registry for Cystic Echinococcosis (RIEC): preliminary results

Tamarozzi Francesca^1^*, Rossi Patrizia^2^*, Galati Fabio^3^, Mariconti Mara^1^, Nicoletti Jacopo^1^, Rinaldi Francesca^1^, Casulli Adriano^2^, Pozio Edoardo^2^, Brunetti Enrico^1^

*These authors contributed equally to this work.

^1^Department of Clinical, Surgical, Diagnostic and Paediatric Sciences, University of Pavia & WHO Collaborating Centre for the Clinical Management of Cystic Echinococcosis, Pavia, Italy

^2^Department of Infectious, Parasitic and Immunomediated Diseases, Istituto Superiore di Sanita, Rome, Italy

^3^SIDBAE, Information Technology, Istituto Superiore di Sanita, Rome, Italy

francesca.tamarozzi@unimi.it; enrico.brunetti@unipv.it

**Background:** Cystic Echinococcosis (CE) is endemic in Eastern and Southern Europe, Italy included. However, its real prevalence and incidence are largely unknown due to the lack of efficient reporting systems designed for the peculiarities of this disease. Indeed, despite being a notifiable infection in animals, being listed among the reportable professional diseases, and being subject to surveillance according to the European legislation, in Italy the notification of human cases ceased to be compulsory in 1991 and only a yearly summary of regional data is required by the health authorities. As a result, no official data are transmitted to European authorities, witness the 0 (zero) cases in the 2013 annual report of ECDC while also epidemiological data in livestock are incomplete. Even at national level, the prevalence and incidence of human CE are hugely underestimated because only hospitalized cases are registered (963 hospital discharge records with a diagnosis of CE in 2011 were retrieved from the Italian Ministry of Health website). However, this reporting modality is inadequate and only detects the “tip of the iceberg”, as the majority of CE cases are diagnosed and managed on an outpatient basis. Furthermore, the neglect of CE also results in general lack of knowledge on its diagnosis and clinical management outside referral centres, with consequent heterogeneity in clinical practices and often unnecessary procedures with associated risks and costs.

**Methods:** To start tackling this long-standing problem, in 2012 the Istituto Superiore di Sanità (ISS – Italian National Health Institute – Rome) in collaboration with the University of Pavia, WHO Collaborative Centre for the Clinical management of Cystic Echinococcosis, implemented the Italian Registry of Cystic Echinococcosis (RIEC). This is a prospective multicenter registry of CE patients visited from January 2012 in Italian health centres which adhered to RIEC. RIEC was published on the website of the ISS in March 2013 with the aims of: indicating the burden of CE in Italy; bringing to the attention of health authorities the importance of this neglected infection; encouraging public health policies toward its control; stimulating research on CE. Moreover, it provides a useful tool for patient follow-up and evaluation of therapeutic interventions.

**Results:** As of December 2013, 315 patients had been enrolled in just 8 centres, with this figure alone largely outnumbering the national reports of many endemic European countries. So far, the majority of records were entered in Pavia Hospital, WHO Collaborative Centre for the Clinical management of Cystic Echinococcosis.

**Discussion:** Preliminary data and challenges will be discussed, with the perspective of using RIEC as a template for the European Registry of CE, to be implemented within the European FP7 HERACLES project (2013–2017).

##### O-24. A new data management system for the FrancEchino human cases registry

Charbonnier Amandine^1,2^, Knapp Jenny^1^, Demonmerot Florent^1^, Bresson-Hadni Solange^1^, Raoul Francis^1^, Grenouillet Frédéric^1^, Millon Laurence^1^, Damy Sylvie^1^

^1^Chrono-environment Laboratory, UMR 6249 University of Franche-Comté and CNRS, Besançon, France

^2^OSU THETA Franche-Comté Bourgogne, Besançon, France

chrono-env@univ-fcomte.fr; direction@obs-besancon.fr

**Background:** Alveolar echinococcosis in France is responsible for an average of 18 new cases per year, representing an annual incidence of 0.26/1’000’000 habitants with a tendency to increase since this last decade. The FrancEchino network has collected medical and epidemiological data from 1982 onwards on residents on the French territory to better understand the circumstances around the discovery of the disease in patients. Currently 567 patients are registered (2013–2011). These data are available so far under the shape of spreadsheets. Even if basic management can be undertaken with spreadsheets by respecting some rules, this solution does not allow insuring quality of information, tracing, sharing, manipulating and securing data.

**Methods:** To solve these problems we created a database and its online web application. The conceptual data model associated describes all the data and the interactions between them. A model is the common communication medium for each stakeholder in the project. This model takes into account all the data present in the existing spreadsheets. The database is a MySQL database and the web application is developed in PHP. The web application allows performing quickly and with much less effort a large number of previously time-consuming tasks. For example, a dedicated form to add a patient increases the input while correspondingly reduces the probability of errors.

**Results:** The database integrates data such as INSEE (National Institute for Statistics) data for the management of geographical location (country, municipality ...) or of occupation, and it makes possible, for instance, a more accurate follow-up of the patient such as the chronicle of the progression or the list of medication in the treatment. The database must be open to various kinds of users under conditions. Access on data is made by a user management in the web application, in the database and on the hosting server. The hosting server and the database management system have means to archive data and make backup/restoration. Three types of users are defined with specific access possibilities: CNR (National Reference Centre) users can access all data and modify them, medical doctors belonging to the FrancEchino network can consult data related to their patients, and the general public can only access synthesis data. The main functionalities of the application are the addition, modification or removal of patients, the consultation of individual or synthetic information, the export of data and the generation of graphics and statistics for the CNR reports.

**Discussion/Conclusion**: This new database is a reliable and flexible tool to manage simply, efficiently and in a safe secured way the medical and epidemiological data and has the potential to include more similar data.

##### O-25. The ecology of public health: exploring transmission dynamics of *Echinococcus multilocularis* in a North American urban setting

Massolo Alessandro^1^, Liccioli Stefano^1,2^, Smith Anya^1^, Klein Claudia^1^

^1^Department of Ecosystem and Public Health, Faculty of Veterinary Medicine, University of Calgary, AB, Canada

^2^Department of Biological Sciences, Faculty of Science, University of Calgary, AB, Canada

amassolo@ucalgary.ca

**Background:** The ecology of *Echinococcus multilocularis* is key when assessing the risk of transmission to humans. Zoonotic transmission is more likely to occur in urban environments. Most of human alveolar echinococcosis (HAE) cases in North America has been recorded in the Tundra region, and only occasionally in the central continental region. No studies have been carried out to assess the prevalence and transmission dynamics in wild (fox, coyote, rodents) and domestic hosts (dogs) in urban settings.

**Methods:** In 2009 we started a long term research program on the transmission of parasites at the interface of wildlife, domestic animals and people in urban settings, using *E. multilocularis* as model for trophically transmitted parasites. We investigated parasite presence in carcasses collected within the city of Calgary, and from June 2012 to June 2013 we searched for *E. multilocularis* eggs in coyote feces (*N* = 385) collected in five city parks along standardized pathways, as well as parasite metacestodes in wild rodents collected in the same sites.

In 2010 and 2012, we engaged dog owners in collecting fresh dog fecal samples to study dog parasitism in Calgary. In 2010, 635 owners were asked about their perception of risk of parasitic transmission while walking their dogs in parks. In 2012, we surveyed 1,300 dog owners on their dog-walking habits.

**Results:** The prevalence in urban coyote carcasses was 40%. We detected endemic fecal infections in coyotes in city parks (from 5.3% to 61.5%), associated to infections in three intermediate host species (from 0.66 to 1.41%). Infections in coyotes were spatially and temporally heterogeneous with a high endemicity site (up to 83.8% in Autumn). After a HAE case report in Edmonton (May 2013), we searched for *E. multilocularis* in 100 dogs with at risk behavior (high off leash activity; predation on rodents) detecting a positive case, and the concrete possibility of zoonotic transmission through domestic dogs. The survey on dog owners indicated that their perception of risk of parasitism for their dogs and for their family members was very low and did not affect their dog walking behavior. Parasites (adult worms, cysts or eggs) from coyotes, rodent carcasses and coyote or dog feces are undergoing strain typing. Preliminary results indicated that most of the infections in coyotes (11 out of 12) and all the ones in rodents (3 out of 3) were due to the European strain of *E. multilocularis.*

**Discussion/Conclusion:** We detected the presence of an endemic sylvatic cycle of *E. multilocularis* in an urban setting in North America, with high endemicity foci, and infections in domestic dogs. The presence of a HAE case, and the detection of the European strain increase the risk of an emergent outbreak of HAE in urban areas in Western Canada.

##### O-26. Modelling *Echinococcus multilocularis* abundance in foxes in Zurich

Otero-Abad Belen^1^, Hegglin Daniel^2^, Deplazes Peter^2^, Torgerson Paul^1^

^1^Section for Veterinary Epidemiology, Vetsuisse Faculty, University of Zurich, CH-8057 Zurich, Switzerland

^2^Institute of Parasitology, Vetsuisse Faculty, University of Zurich, CH-8057, Zurich, Switzerland.

paul.torgerson@access.uzh.ch; deplazesp@access.uzh.ch

**Background:** *Echinococcus multilocularis* (EM) is an endemic parasite in red foxes (*Vulpes vulpes*) in the northern hemisphere. Humans can become infected through the accidental ingestion of EM eggs, causing a serious parasitic zoonosis called alveolar echinococcosis (AE). The increased reporting of AE cases in endemic areas of Europe coincides with the growth of fox populations and their expansion towards the urban areas. In this study transmission models were fitted to EM abundance data from foxes collected in Zurich (Switzerland). Through the modelling of parasite counts we estimate the value of key infection parameters and provide an additional insight into EM infection dynamics in foxes.

**Methods:** The data set encompassed the worm counts of 458 red foxes of less than three years old. This age group was selected deliberately as during the exploratory analysis it displayed the majority of the zoonotic risk. The foxes were collected during a previous observational study undertook between 1996 and 2000 within the political community of Zurich. The study area was divided into three zones, periurban, border and urban, based on the level of urbanization. Forty-two age-based abundance models, originally developed by Roberts et al. (1986) and later modified by Torgerson et al. (2003), were fitted to the data. Through the employment of maximum likelihood estimation techniques (MLE) epidemiological parameters were calculated. Model selection was performed using the Akaike information criterion (AIC), which assesses the goodness of fit of the model while penalizing for the addition of parameters.

**Results:** The best fit for the EM abundance data was given by a model accounting for spatial differences in infection pressure among urban zones and assuming presence of parasite-induced immunity only in foxes from the periurban area. The preferred model presents a periodic infection pressure in function of fox age over all three zones. Model’s estimate for parasite loss rate was similar to the one experimentally measured by Kapel et al. (2006).

**Discussion:** Multiple factors, such as habitat, diet and low densities of suitable intermediate prey-hosts, might contribute to the marked spatial differences in parasite distribution among areas. Indications of acquired immunity in foxes from areas with high infection pressure, like the periphery of cities, is in line with the hypothesis that foxes might acquire partial immunity after repeated exposure. Evidence of periodic infection pressure is consistent with several studies reporting seasonal variation in EM prevalence in foxes.

#### Posters

##### P-16. Linking ecosystem health and environmental disease ecology: the International Research Network “Ecosystem Health and Environmental Disease Ecology” (IRN-EHEDE)

Giraudoux Patrick, for the IRN EHEDE

Chrono-environment Laboratory, UMR 6249, University of Franche-Comté, Besançon, France, and Institut Universitaire de France, Paris, France

patrick.giraudoux@univ-fcomte.fr; http://gdri-ehede.univ-fcomte.fr

The International Research Network (IRN) or Groupement de recherche international (GDRI) « Ecosystem health and environmental disease ecology » has been created and accredited by the CNRS in 2013, with the objective of promoting exchanges and bringing better legibility to research in Asia and Europe linking ecosystem health (e.g. the long-term sustainability of ecological processes and the integrity of ecosystem services) and disease ecology (e.g. the processes by which diseases can be maintained or controlled in a given ecosystem).

This network brings together specialists of 16 research departments of 6 countries (Australia, China, France, Germany, Japan, UK) in conservation, population biology, landscape and community ecology, geography, parasitology, modelling and health sciences. It also mobilises networks of stakeholders in agriculture, conservation and public health to address questions related to the multi-scale anthropogenic disturbance of regional ecosystems.

Three specific issues are currently covered: (1) Ecology of Cestode transmission in Asia and Europe, (2) Ecosystem health and wildlife management (3) Permanent workshop on adaptive monitoring and data management.

We believe that earlier and current results obtained about interactions between landscape, small mammal and carnivore population dynamics and the transmission of pathogen agents, especially *Echinococcus multilocularis*, provide a solid ground and concepts for broader ecosystem studies and research development on other pathogens and other animal communities, and also for applying those concepts and methods to conservation issues. Stress is on the long term monitoring of host and pathogen parasite population dynamics on local and regional scales, on data management and on advanced methods for statistical and spatial modelling, including those combining remote sensing, GIS and molecular genotyping to characterize populations. Long term monitoring and multi-scale investigation are crucial for the improved management of ecosystems and natural resources (e.g. in the context of global changes, etc.) and also for public health actions (disease emergence, etc.). Data were collected consistently in Europe and Asia, in the spirit of adaptive monitoring schemes during earlier programmes by IRN EHEDE members. This allows us to develop new programmes about the long term evolution of ecosystem health and disease transmission sometimes 10–20 years after the baseline was established (e.g. in France, Ningxia, Gansu, etc.).

##### P-17. In vivo viability testing of *Echinococcus multilocularis* eggs in a rodent model after different thermo treatments

Federer Karin^1^, Armua Fernandes Maria Teresa^1^, Wenker Christian^2^, Hoby Stefan^2^, Deplazes Peter^1^

^1^Institute of Parasitology, University of Zurich, Zürich, Switzerland

^2^Zoo Basel, Basel, Switzerland

karin.federer@walterzoo.ch

**Background and aims:** Great apes and nonhuman primates are highly susceptible for alveolar echinococcosis (AE), and this disease is emerging in zoos in central Europe. Risk factors for *Echinococcus* infections in zoos are still unknown. Most of the zoos are not strictly fox free, and therefore an environmental contamination in the zoos with E. multilocularis eggs cannot completely be excluded. However, a more probable source of infection is food, especially vegetables contaminated with worm eggs. Treatment of the raw vegetables with heat was hypothesized a feasible method to decontaminate food items for primates. The goal of this study was to develop a sensitive in vivo method to determine the viability of *E. multilocularis* eggs and to determine suitable conditions (optimal temperature, exposure time and humidity) to inactivate *E. multilocularis* eggs with minimal alteration of the nutritional properties of the food.

**Methods:** In the first part of the study, the sensitivity of a rodent model (Black6 mice) was evaluated. These mice were more susceptible to subcutaneous inoculation of sodium hypochlorite resistant oncospheres than to oral inoculation of eggs.

In the second part of the study, various combinations of exposure temperature (between 45 °C and 65 °C), time (between 30 min and 3 h) and humidity (70% vs. suspended in water) were tested. After heat treatment in an incubator, the sodium hypochlorite resistance test was used to assess in vitro egg viability at the time of inoculation. Subsequently, the infectivity of the oncospheres was evaluated by subcutaneous inoculation of mice. Eggs exposed to increasing temperatures were more resistant if suspended in water as compared to eggs exposed on a filter paper at 70% humidity. As survival of eggs in water drops in the vegetables cannot be excluded, further experiments were performed with eggs suspended in water only.

**Results:** Eggs were infectious after exposure up to 2 hours at 65 °C, however, no echinococcosis developed after treatment of the eggs for 30 min at 70 °C.

**Discussion/Conclusion:** The suitability of these conditions to decontaminate various primate food items needs further evaluation.

##### P-18. Study of resistance of *Echinococcus multilocularis* oncosphere invasion in a rat model

Armua Fernandez Maria Teresa, Schweiger Alexander, Eichenberger Ramon, Deplazes Peter

Institute of Parasitology, University of Zurich, Zürich, Switzerland

mtarmua@vetparas.uzh.ch

**Background and aims:** Human alveolar echinococcosis has a long incubation period of 5–20 years, with a slow parasite growth progression mainly in the liver. Meanwhile, a number of intermediate hosts (e.g. mice and voles) are highly susceptible to infection and parasite development; rats as humans seem to be resilient to infection. Factors that influence the success or failure in oncosphere invasion and metacestode development are still not well understood. Rats have been shown to be highly resistant to E. multilocularis in nature, but they could be a suitable rodent model for parasite propagation after intraperitoneal inoculation.

**Methods:** In this work, experiments in rats were carried out to study different factors that can influence success of oncosphere invasion and metacestode development. Two types of materials were used: eggs and metacestode tissue. The viability of eggs was assessed by the sodium hypochlorite resistant test. Intraperitoneal and oral infections were carried out using Wistar rats (immunocompetent and iatrogenically immunosuppressed) and also in athymic nude rats (Hsd:RH-Foxn1^rnu^). The rats received by intragastric inoculation from 900 to 10000 hypochlorite-resistant oncospheres depending on group. The immunosuppression treatment started 2 weeks prior and lasted until the 6th week post inoculation.

**Results:** Intraperitoneal inoculation with metacestode material resulted in metacestode infections, independent of the age of rats. In orally inoculated rats (eggs or free oncospheres) no macroscopic evidence of *E. multilocularis* development in both immunocompetent and immunodeficient nude rats was found. However, for the first time, a successful infection after oral inoculation of eggs was observed in 2 out of 6 rats with iatrogenically-induced immunosuppression. Using a crude *E. multilocularis* metacestode antigen from canine origin, only these two mentioned rats seroconverted 16 weeks after egg inoculation.

**Conclusion:** According to our results we hypothesize that innate immunity plays a paramount role during oncosphere invasion.

##### P-19. Changes in *Echinococcus* transmission patterns in a community hyper-endemic for echinococcosis in China

Liu Can^1^, Clements Archie^2^, Gray Darren^2^, Barnes Tamsin^3^, Raoul Francis^4^, Giraudoux Patrick^4^, McManus Donald^5^, Williams Gail^2^, Yang Yurong^1,5^

^1^Ningxia Medical University, Yinchuan, Ningxia, China

^2^School of Population Health, University of Queensland, Brisbane, Australia

^3^School of Veterinary Science, University of Queensland, Brisbane, Australia

^4^UMR 6249 Chrono-environment, University of Franche-Comté, Besançon, France

^5^Molecular Parasitology Laboratory, QIMR Berghofer Medical Research Institute, University of Queensland, Brisbane, Australia

yangyurong@hotmail.com

**Objective:** A study to investigate *Echinococcus* transmission dynamics was undertaken in 2000–2003 and 2012 in a hyper-endemic community (Xiji County), Ningxia, China was performed in animalntermediate hosts populations (small mammal species for *E. multilocularis*; slaughtered livestock for *E. granulosus*) and humans (teenagers).

**Methods:** Human cases cannot be diagnosed by serology alone but seroprevalence was considered as a marker of human *Echinococcus* exposure. Small mammals were trapped, index line transects were walked to detect small mammal indices, their trapping location geo-referenced and habitat characteristics recorded. Animals were dissected to identify potential lesions of larval *Echinococcus* sp. Inspection records of livestock diseases from the slaughter houses of Xiji County were searched for the period 2000–2003. In 2012, due to economic policy changes and the subsequent closure of the majority of public slaughterhouses, the survey was undertaken in Xinglong, the largest outdoor livestock slaughter market in Xiji.

**Results:** Land cover only marginally changed from 2003 to 2012; however some growth was evident in reforested areas gradually shifting from set-aside farmland to bushes. No small mammals were found infected during the survey. There was a decrease in the *Spermophilus* (ground squirrel) population but a large increase of *Myospalax* (Zokor) populations in reforested areas between 2003 and 2012. Taking both trapping and transects results into account, Cricetulus longicaudatus (long-tailed dwarf hamster) represented 57–70% of the animals trapped in the small mammal community for the two periods considered. Overall the survey indicates a virtual absence of Arvicolid species in Xiji. The prevalence of *E. granulosus* in slaughtered sheep in Xiji appears to have decreased dramatically from around 20% (*n* = 877) in 2000–2003 to 0% (*n* = 198) from personal attendance at the Xinglong outdoor slaughter market in 2012. However, there was increased sero-positivity to *Echinococcus* antigens in teenagers, suggesting increased exposure to infection in the survey area (28% in Chengjiao [*n* = 59] and 30% in Haoziwan [*n* = 87] in 2000–2003 compared with 50% in Chengjiao [*n* = 200] in 2012).

**Conclusions:** Our study confirms the dominance of Cricetine (hamster) species and the virtual absence of large Microtine species on farmland in Xiji. It indicates that land cover changes have led to a large increase in Myospalax populations in set-aside fields and shrubby habitats. It also demonstrates a substantial increase of anti-*Echinoccocus* antibodies in the teenage population. Paradoxically, an apparent decrease in sheep infection was observed. This discrepancy raises the issue of the nature of the driver(s) responsible for this increase. Further studies are necessary to answer this question.

##### P-20. Current situation concerning the prevalence of *Echinococcus multilocularis* in red foxes in Poland*

Karamon Jacek, Sroka Jacek, Cencek Tomasz, Różycki Mirosław, Chmurzyńska Ewa, Bilska-Zając Ewa

National Veterinary Research Institute, Puławy, Lubelskie, Poland

j.karamon@piwet.pulawy.pl

**Background and aims:** The aim of the study was to determine the prevalence of *Echinococcus multilocularis* in red foxes in Poland.

**Methods:** Overall, 1546 intestinal samples of red foxes (collected in years 2009–2013) from 15 of the 16 provinces in Poland were examined by the sedimentation and counting technique (SCT).

**Results:** The mean prevalence of *E. multilocularis* in Poland was 16.5% (CI 14.7–18.4%), and was found in 14 of the 15 examined provinces. Distinct differences in prevalence were observed between regions. There was some observable regularity in the distribution of the infection in Poland, namely that those areas with a higher prevalence were located in the eastern half of the country while those areas with a lower prevalence were in western half. The border between these zones seems to lie on a north-south line running through the middle of Poland. The highest level of prevalence was noted in the north-eastern Poland in Warminsko-Mazurskie Province – 50.0% (CI 40.3–59.7%). Very high prevalence was also observed in Podlaskie – 34.0% (CI 25.5–43.7%) and Mazowieckie – 30.8% (CI 23.3–39.6%), as well as provinces in southern Poland: Małopolskie – 30.0% (CI 20.6–38.2%) and Podkarpackie – 47.2% (CI 37.9–56.6%) while being relatively high in Świętokrzyskie – 17.4% (CI 11.2–26.3%). The comparison of our results obtained from the eastern half of Poland with the results of investigations conducted in the same area in the past demonstrated a very distinct and dynamic rise in *E. multilocularis* prevalence in red foxes during the past 15–20 years. Moreover, it can be observed that the rapid increase in the percentage of infected foxes began in the late 1990s and early 2000 s. The situation concerning the prevalence of *E. multilocularis* in foxes was different for the western half of Poland. The percentage share of infected foxes in the western, north-western, south-western and partially central provinces was relatively low, for example: Dolnośląskie – 2.0% (CI 0.3–7.6%), Wielkopolskie 2.5% (CI 0.9–7.6%), Kujawsko-pomorskie – 3.9% (CI 1.5–9.6), Opolskie – 0.0% (CI 0.0–3.7%). However, it is interesting to note that, in contrast to the eastern regions, the western half of Poland lacked the significant increase in the number of infected foxes – the low prevalence in these areas has remained at the same level (or increased minimally) for about 15 years.

**Discussion/Conclusion:** Investigation shows the risk for the human health connected with these parasites in Poland, especially in those regions with a very high prevalence. It must be stressed that most cases of human alveococcosis in Poland have been observed in the Warminsko-Mazurskie Province where 50% of foxes were infected. The dynamic situation observed in the prevalence of this tapeworm indicated the necessity of continuing to monitor the situation concerning *E. multilocularis* in red foxes in Poland.

*More detailed description of results was published: Karamon et al. Parasitol Res. 2014, 113:317–22.

##### P-21. *Echinococcus multilocularis* screening of dog populations in France, a multiscale approach revealing inappropriate deworming practices

Comte Sébastien^1^, Umhang Gérald^2^, Raton Vincent^1^, Hormaz Vanessa^2^, Boucher Jean-Marc^2^, Favier Stéphanie^1^, Combes Benoît^1^, Boué Franck^2^

^1^Entente de Lutte Interdépartementale contre les Zoonoses (ELIZ), 54220 Malzéville, France

^2^Wildlife pathology unit & National Reference Laboratory for Echinococcus spp., ANSES Nancy Laboratory for Rabies and Wildlife, 54220 Malzéville, France

franck.boue@anses.fr

**Background:** Alveolar echinococcosis is a zoonosis of major concern for health authorities in Europe. The life cycle of its parasite *Echinococcus multilocularis* is based on predator-prey interactions, mainly between foxes and rodents. Dogs are also reckoned to be very suitable as definitive host. Due to their close proximity to humans, they represent a high zoonotic risk that should be precisely characterized. During the last decade, we have investigated the presence of *E. multilocularis* in France.

**Methods and Results:** Based on a multiple scale approach (*Département*, middle-size cities and small villages) involving private veterinarians, more than 1800 dog feces were collected in highly endemic areas (40%–60% of foxes being infected). All samples were analysed by a combination of flotation and PCR techniques. Dog fecal contamination was assessed in five stools resulting in prevalence ranging from 0 to 0.5%, similar to most other West European studies. For each feces analyzed, a questionnaire was addressed to the dog owner with focus on dog activities and deworming practices. In the high endemic context of our studies, we highlighted a general underestimated risk of contamination of the dogs. This in turn may be the source of inappropriate pattern of anthelmintic treatment with most of the dogs being dewormed only two times a year when they should be treated in a monthly routine. This particular point was even more worrying for owners of hunting dogs which appeared to be significantly more infected than non-hunting dogs.

**Discussion/Conclusion:** We assume that most of the dog owners accurately follow the recommendations given by their veterinarians and hunting associations. We consequently recommend that, in highly endemic areas, information should be adapted to prevent dog contamination and avoid human transmission.

##### P-22. Evaluation of the infection by *Echinococcus granulosus* in stray dogs in the region of Algiers: ante- and post-mortem exams

Ghalmi Farida, Zebiri Essma, Sekat Nawel Isma

Higher Veterinary School of Algiers, El Harrach, Algiers, Algeria

fghalmi@yahoo.fr

**Background:** In Algeria, the dog is the major reservoir of *Echinococcus granulosus* infection for domestic animals and for humans (the intermediate host). Presently, there is no estimation of the infestation rate in dogs although the prevalence in definitive host is the more reliable indicator or the potential risk of transmission to humans and the others intermediate hosts.

**Methods:** In this work, 192 stray dogs of the Algiers region were exanimate for the presence of Echinococcus granulosus. The feces were analyzed and the dogs were euthanized and a post mortem analysis was performed.

**Results:** The results indicated a prevalence of 14.8% of Taenia spp. in feces. The necropsy indicated the presence of the adult worm of Echinococcus granulosus in 21% of the dogs.

##### P-23. Genetic diversity of *Echinococcus* spp. in Russia

Konyaev Sergey^1^, Yanagida Tetsuya, Nakao Minoru, Sako Yasuhito, Ito Akira

^1^Institute of Systematics and Ecology of Animals, SB RAS, Novosibirsk, Russian Federation

^2^Department of Parasitology, Asahikawa Medical University, Asahikawa, Hokaido, Japan

s.konyaev@yahoo.com

**Background and aims:** In Russia, both CE and AE are endemic in humans and domestic/wild animals, and E. granulosus s.l. and E. multilocularis have long been recognized as the causative agents of human echinococcoses. Since the dissolution of the Soviet Union in 1991, the number of human echinococcosis cases in Russia increased rapidly. While only 190 new cases were reported in 1992, the recorded cases reached 553 in 2001. Among them, only 30 cases were alveolar echinococcosis. The total incidence rate of both echinococcoses in Russia was 0.4 cases per 100,000 persons in 2001 and is still keeping in this high point during the last decade. The incidence rate is above the average in some regions such as Chukotka, Yakutia, Altai region, Yamal-Nenetskiy Area, Bashkiria, Karachaevo-Cherketskaya Republic. However, the serological studies demonstrated that the number of cases is underestimated, and it can be as much as three times higher. Therefore, extensive epidemiological survey is needed to understand the current situation of echinococcoses in Russia. For the epidemiology of echinococcoses, a reliable molecular method to identify the etiological agents is necessary because of the morphological similarity among *Echinococcus* species. However, molecular identification of *Echinococcus* species and genotypes has rarely been made in Russia until recently. The present study aimed to identify the etiological agents of the diseases and to investigate the distribution and host range of each parasite species in Russia.

**Methods:** A total of 61 *Echinococcus* specimens were collected from 14 host species including humans. Based on the mitochondrial gene sequences, they were identified as *Echinococcus granulosus, E. multilocularis* and *E. canadensis*.

**Results:** *E. granulosus* was found in humans, sheep and a cat. Three genotypes of *E. multilocularis* were confirmed; the Mongolian genotype from voles in Baikal lake island and Altai Republic, the Asian genotype from humans, voles, wolves and red foxes in the areas close to Asian countries and also from red foxes in the European part, and the North-American genotype from arctic foxes in Yakutia, Far East Siberia. The three genotypes of *E. canadensis* were detected in Yakutia; G6 from domestic reindeer, G8 from moose and G10 from moose and wolves. G6 was also found from humans and wolves in Altai region.

**Discussion/Conclusion:** The rich genetic diversity of *Echinococcus* spp. demonstrates the importance of Russia in investigating epidemiological importance of genotyping studies.

##### P-24. Diagnosis of *Echinococcus* spp. in dogs using specific LAMP assays

Yang Yurong^1,2^, Jia Wangzhong^3^, McManus Donald^1^

^1^Molecular Parasitology Laboratory, QIMR Berghofer Medical Research Institute, Brisbane, Queensland, Australia

^2^Ningxia Medical University, Yinchuan, Ningxia, China

^3^Gansu Provincial key Laborary of Veterinary Parasitology, CAAS, Langzhou, Gansu, China

yangyurong@hotmail.com

**Objectives:** Human alveolar and cystic echinococcosis (AE and CE) are highly prevalent in northwest China. A cost-effective, easy-operated diagnostic tool with high sensitivity and specificity would greatly facilitate the monitoring of *Echinococcus* infections in canids in order to determine the infection pressure to humans and intermediate hosts due to egg contamination of the environment. We have developed a Loop-Mediated Isothermal Amplification (LAMP)-based assay which can provide the requisite diagnostic performance.

**Methods:** The primers used in the LAMP assay were based on the mitochondrial nad5 gene of *E.multilocularis* (*E.m*) and *E. granulosus* (*E.g*). G1 strain (a common strain globally) and were designed using Primer Explorer V4 software. The developed LAMP assay was compared with a conventional PCR method, using DNA extracted from the faeces of dogs experimentally infected with either *E.m.* or *E.g.* to determine the level of sensitivity. We used several sources of DNA samples to assess the specificity of the LAMP assay. The first comprised various extracted parasite DNAs (including E. g. [G1,G4, G6/G7 genotypes], *E.m., E. shiquicus, T. hydatigena, T. pisiformis, T. taeniaeformis, T. multiceps* and *Dipylidium caninum*). The second comprised DNAs extracted from field collections of domestic dog faeces, including 190 faeces from an area in Qinghai endemic for both E.m and E.g., and 30 faeces, confirmed as free of *Echinococcus* infection by necropsy, from an area in Gansu, where there had been in operation a domestic dog de-worming program over 2 years. Additionally, we also used negative control DNA extracted from uninfected dog faeces obtained from newly born pups in the laboratory. The concentrations of all DNA samples were determined using a Nanodrop 2000 spectrophotometer.

**Results:** The positivity rates obtained for the field-collected faecal samples were 16.4% and 5.3% for *E.m.* and 12% and 1.6% for *E.g.* by the LAMP and PCR methods, respectively. All samples obtained from the control dogs were negative. Compared with conventional PCR, the LAMP assay provided 88.3% specificity and 100% sensitivity. The higher sensitivity of the LAMP method was also shown by the fact it could detect the presence of laboratory challenge dog infections of *E.m.* or *E.g.* 4 and 5 days earlier than the PCR method.

**Conclusion:** The earlier specific detection of an *E. m.* and *E.g.* (G1) infection in dog copro-samples indicates that the LAMP assays we developed are a realistic alternative method for the field surveillance of canines in echinococcosis-endemic areas.

##### P-25. Microsatellite genetic polymorphism of *E. granulosus* isolates from three endemic regions in Tunisia: preliminary results

Bennour-Ben Abdeljelil Abir^1^, Oudni-M’rad Myriam^1^, M’radn Selim^1^, Nouri Abdellatif^2^, Ben Algia Wissem^3^, Mekki Monji^2^, Belguith Mohsen^2^, Mezhoud Habib^1^, Babba Hammouda^1,4^

^1^Laboratoire de Parasitologie-Mycologie Médicale et Moléculaire (LP3M), LR12ES08, Faculté de Pharmacie, Université de Monastir, Monastir, Tunisie

^2^Service de Chirurgie pédiatrique, E.P.S Fattouma Bourguiba, Monastir, Tunisie

^3^Abattoir Municipal, Kairouan, Tunisie

^4^Laboratoire B, Centre de Maternité, E.P.S. Fattouma Bourguiba, Monastir, Tunisie

myriam.mrad@gnet.tn

**Background:** Cystic echinococcosis is a major public health problem in Tunisia. *Echinococcus granulosus* is a complex in which four or five cryptic species are intermixed. Two species have been described in Tunisia *E. granulosus sensu stricto* in humans, sheep, cattle and dromedary hosts and *E. canadensis* in dromadaries. The G1 genotype is predominant in Tunisia and genetic variability within this genotype has been demonstrated. The aim of this study is to investigate the genetic variation within and between *E. granulosus* sheep, cattle, goat and human populations in Tunisia by using five microsatellite markers: Egmsga1, Egmsca1, EMms2, EMmsB and U1snRNA.

**Methods:** Parasitic material consisted of hepatic and pulmonary fertile cysts from human, ovine, goat and bovine in Tunisia. 39 cysts from children operated at Monastir teaching hospital, 108 cysts from sheep, 27 from cattle and 3 from goat slaughtered at the abattoirs of Kasserine, Gafsa and Kairouan were collected. The genomic DNA obtained from each isolate was analysed by using a direct PCR-based approach and sequencing. The electrophoretic data were interpreted in terms of Fst and Cavalli-Sforza genetic distances.

**Results:** For the Egmsga1 microsatellite, the same band was found on all sample tested, indicating the absence of genetic polymorphism. The EMms2 microsatellite sequence of *E. granulosus* isolates presented 73% homology with the *E. multilocularis* reference sequence and a difference in size due to the deletion of 21 nucleotides. The (CAC)_N_ tandem repeat, identified in *E. multilocularis* genome, is absent. For the Egmsca1 microsatellite marker, eight and ten CA repetitions have been observed. Three distinct phenotypes were recorded (CA_8_/CA_8_, CA_10_/CA_10_ and CA_8_/CA_10_ individuals), independently of the host origin or the localization (liver or lung) of the cysts. The U1snRNA and EMmsB multilocus sequences presented respectively three and four different electrophoresis patterns. Various profiles were observed within the same organ.

**Discussion/Conclusion:** Genetic polymorphisms were detected between our isolates but they were not related to the host or the location of the cyst (lung and liver). Moreover, no genetic structuration (genetic differences between regions) due to geographic distance could be observed. Analysis of multilocus microsatellite has proven to be a powerful and interesting tool for genetic polymorphism studies of *Echinococcus granulosus*.

##### P-26. Retrospective study of human cystic echinococcosis in Italy based on hospital discharge record between 2001 and 2011

Brundu Diego, Piseddu Toni, Masu Gabriella, Ledda Salvatore, Masala Giovanna

Istituto Zooprofilattico Sperimentale della Sardegna & Italian National Reference Centre for Echinococcosis (CeNRE), 07100 Sassari (SS), Italy.

brundu.diego3@gmail.com; cenre@izs-sardegna.it

**Background:** Cystic Echinococcosis (CE) is an important zoonotic parasitic infection. CeNRE analyzed Hospital Discharge Records (HDRs) drawn from the National Ministry of Health. The aim of this study is analyze data of HDRs with CE related diagnosis in Italy in 2001–2011 to assess the current status and trend of disease epidemiology.

**Methods:** 15,825 HDRs of 10,237 patients were analyzed according to the patient’s place of residence (Regions, Provinces) in order to evaluate the annual incidence rates of hospital cases (AIh) in different administrative divisions and in rural and urban areas (Tab.1). Furthermore, Provinces were classified according to the IDs index, IDS = Nsh × Df (Nsh = average number of sheep per farm; Df = density of sheep farms per Km^2^), it was divided into quartiles in order to evaluate correlation between the human CE incidence and presence of sheep breeding. We use direct method of standardization (age-gender) to compare AIh between rural and urban areas and between the quartiles of IDs index. The confidence intervals (CI) for the relative risk (R.R.) was calculate by methods described by Armitage and Berry with 95% CI.

**Results:** The highest average AIh was registered in the Islands 4.95/10^5^ inhabitants (7.2 in Sardinia and 4.2 in Sicily), followed by South with a average AIh of 2.1/10^5^ inhab. (5.6 in Basilicata) and the Centre 1.2/10^5^ inhab (1.7 in Latium). The analysis for trend showed a statistically significant decrease in the AIh throughout the study period (eg. in the Islands *r* ^2^ = 0.96, *p* < 0.001). An AIh over 2 cases/10^5^ inhab. was observed in 32/110 Provinces. Provinces with “rural areas with comprehensive development problems” had a relative risk of CE of 7.02 (95% CI, 6.31–7.81) compared to Provinces with urban areas. R.R. increases when we compare areas where sheep breeding is widespread to those it is less widespread.

**Conclusion:** This study underlined as situation of human CE in Italy continues to be a public health concern. The epidemiological GIS analysis is useful to target preventive measures and to plan a proper CE control programme with a reliable assessment of the degree of intervention needed in various regions. Options for control include horizontal and vertical approaches. The former emphasises long-term primary health care (eg. healt Education). The second is based on specific control measures targeted to the parasite (eg. dog-dosing) and must include a surveillance of intermediate animal hosts to monitor progress. The last control measures developed is the vaccination of intermediate hosts with EG95 recombinant vaccine, which could potentially be used to reduce the level of *E. granulosus* transmission and decrease the incidence of human infections.

##### P-27. Relationship between *Echinococcus granulosus* dog infections and regional characteristics in Tunisia

Chaabane-Banaoues Raja^1^, Oudni-M’rad Myriam^1^, Cabaret Jacques^2^, M’rad Selim^1^, Mezhoud Habib^1^, Babba Hamouda^1,3^

^1^Laboratoire de Parasitologie-Mycologie Médicale et Moléculaire (LP3M), LR12ES08, Faculté de Pharmacie, Université de Monastir, Tunisie

^2^UMR 1282, IAP INRA & Université F. Rabelais, Nouzilly-Tours, France

^3^Laboratoire B, Centre de Maternité, EPS. Fattouma Bourguiba, Monastir, Tunisie

myriam.mrad@gnet.tn; jcabaret@tours.inra.fr; hamouda.babba@gnet.tn

**Background:** Tunisia is considered an Echinococcosis endemic region with an annual surgical incidence of 15 cases per 100,000 inhabitants. The endemic status differs from one region to another. The infection is transmitted via the eggs of *Echinococcus granulosus* which are passed in the faeces of the definitive dog host. Although the infection is propagated by dogs, the persistence of the parasite life cycle is linked to the durability of the *E. granulosus* eggs in the environment. Determining the rate at which eggs are shed into the environment and their capacity to survive is fundamental to ascertain the real endemic status of echinococcosis in an area. Our objectives were to: assess the contamination rate of dog faecal samples in regions of differing endemicity in Tunisia, explore factors which may explain differences in the contamination index of dogs between regions and finally relate the human incidence with dog contamination.

**Methods:** 1095 dog faecal samples were collected from four different climatic zones in Tunisia: Kef (sub-humid), Monastir, Sousse and Kasserine (semi-arid), Gafsa, Zarzis and Djerba (arid), and Tataouine (desertic). Parasite eggs were recovered from faecal samples using flotation in modified Sheather’s solution. Taeniid eggs were subsequently identified morphologically. *E. granulosus* DNA was analyzed by Eg1121/1122 PCR. Genotype G1 was confirmed using Egss1 PCR. Relationships between regional characteristics were established using non-parametric Spearman rank correlations. Infection rates, climatic characteristics and livestock density of studied region were analyzed using principal component analysis with MVSP software (Version 3.1. KCS).

**Results:** The overall contamination index of dog faeces by *E. granulosus* was 27.21% and different infection levels were observed between regions (chi-square = 81; ddl = 7; *p* = 0.0006). The Metlaoui region had a significantly higher index (41.6%) than all other regions, followed by Djerba (27.6%). Genetic polymorphisms were observed between the isolates of the G1 genotype of *E. granulosus,* but no genetic structuration due to geographic distance could be found. The PCA analyses based on human prevalence and dog contamination index did not find the two to be related.

**Discussion/conclusion:** Regions with high levels of canine echinococcosis have previously been found to be hypoendemic for human echinococcosis. This could reflect the extension of the endemic zone and the increase of the potential contamination risk for humans and livestock. Echinococcosis in the dog was observed to spread without positive relation to livestock density of the studied regions, showing a higher contamination index in dogs of warmer regions. The development of the dog parasite in dry areas with a limited number of herbivores might be related to the canine high densities in these regions rather than to climatic conditions since the survival of eggs is favored in humid areas.

##### P-28. Genetic diversity of *Echinococcus granulosus sensu stricto* in Armenia

Ebi Dennis^1^, Gevorgyan Hasmik^2^, Wassermann Marion^1^, Romig Thomas^1^

^1^University of Hohenheim, Stuttgart, Germany

^2^Scientific Centre of Zoology and Hydroecology, National Academy of Sciences, Yerevan, Armenia

dennis.ebi@uni-hohenheim.de

**Background:** Most taxa of the *E. granulosus* complex are today transmitted in domestic lifecycles and therefore distributed worldwide. However, the phylogenetic division into species and major genotypes predates by far the domestication of livestock, so all the major taxa must have derived from original wildlife hosts. For *E. granulosus* s.s., it has been proposed that the switch from silvatic to domestic transmission had occurred in western Asia and the Middle East (Nakao et al., 2013). The hypothesis rests largely on the greater diversity of mitochondrial haplotypes there as compared to other regions (Yanagida et al., 2012, Casulli et al., 2012), which points to western Asia as an ancient endemic area.

**Methods:** To add data on this, we analysed 66 isolates of *E. granulosus* s.s. from different regions of Armenia (southern Causasus). Samples originated from humans, cattle, sheep and pigs, and from different regions of the country.

**Results/Discussion:** We identified 38 different haplotypes of the complete mitochondrial cox 1 gene (1609 bp), a diversity, which adds complexity to data from other studies in the region (Yanagida et al., 2012). We present haplotype networks from Armenia and the western Asian region, compare them to data from other parts of the world and discuss the applicability of haplotype diversity as a tool to solve epidemiological and biogeographical questions.

##### P-29. Tibet 2014: is cystic echinococcosis coming to town?

Giordani Maria Teresa^1^,Tamarozzi Francesca^2^, Guglielmini Carlo^3^, Xianzhen Wang^4^, Lissandrin Raffaella^5^, Brunetti Enrico^2^

^1^Infectious and Tropical Diseases Unit, San Bortolo Hospital, Vicenza, Italy

^2^Division of Infectious and Tropical Diseases, San Matteo Hospital Foundation and University of Pavia, Pavia, Italy

^3^Radiology Service, Eretenia Clinic, Vicenza, Italy

^4^Department of Burns, the Affiliated Hospital of Qinghai Medical College, Xining, China

^5^Department of Infectious Diseases, San Matteo Hospital Foundation and University of Pavia, Pavia, Italy

giordanimt@gmail.com; f_tamarozzi@yahoo.com

**Background:** Nomadic populations in Tibet are at high risk of cystic echinococcosis (CE) as they live in close contact with dogs and sheep. This risk is compounded by extreme poverty, poor hygiene and education, lack of .healthcare facilities, diffidence towards health services and preference for traditional medicine. Battery-powered, hand-held ultrasound (US) scanners are available in China for field studies, and treatment with albendazole (for humans) and praziquantel (for dogs) is also available for free. Sheep breeding is currently discouraged and herders are compelled to get rid of livestock, thus they move to town looking for employment.

**Methods:** A collaborative humanitarian project was started in 2003 in Yushu, Qinghai, Oriental Tibetan Plateau, PR China, which includes free medical assistance to nomadic people on an outpatient basis. Beginning in 2007, free ultrasound (US) examinations were also offered. After finding a high rate of CE infection, a more structured cooperation with local staff was set up in 2009 at “Yushu People’s Hospital”, which included treatment and follow-up of infected patients. In 2010, a violent earthquake hit the area, making the program unviable for two years. The program restarted in 2012 in Zaduo among the earthquake refugees, with US irregularly available due to lack of electricity; at the same time, a survey program was run at the Hygiene Department, including US screening and pharmacological treatment with albendazole of the active cysts. CE patients were encouraged to seek this service.

**Results:** In 2007, 578 individuals were screened with US, yielding a 6.7% CE infection rate (39 pt, m/f 12/27, mean age 42.7 ± 14.5 y) Many had active cysts, indicating a high parasitic pressure. In 2009, a screening of 480 new patients yielded a CE rate of 5.6%. Overall, 1058 patients were screened over 2 years, with a 6.23% (66 pt) CE infection rate. Twenty-one patients (45,6% of active CE) had a post-surgical relapse. Selected cases (1 in 2007 and 3 in 2009) were treated with PAIR or catheterisation plus oral albendazole. In 2010, after the earthquake, the hygienic situation in this area worsened. Of the 68 patient scanned in 2012, 6 (8.8%) had cysts. Local doctors reported a 10% prevalence of CE in displaced nomads living in town. No surgery was available at the local hospital damaged by the earthquake, so patients needing surgery were sent to referral Centres elsewhere.

**Conclusions:** As per the government’s statement of purpose (Chin J Schisto Control 2011, Vol. 23, No. 5), efforts are needed to fight CE in this area, by reducing new cases in the pastoralist population and reducing inappropriate surgery by rational allocation to treatment (according to cyst stage and presence of complications).

##### P-30. *Echinococcus ortleppi* in humans and cattle in France: a silent endemic?

Grenouillet Frédéric^1,2,#^, Umhang Gerald^3,#^, Arbez-GIindre Francine^4^, Mantion Georges^5^, Millon Laurence^1,2^, Boué Franck^3,#^

^1^National Reference Centre for Alveolar Echinococcosis & WHO Collaborating Centre for Prevention and Treatment of Echinococcosis, Parasitology-Mycology Department, University Hospital, 25030 Besançon, France

^2^UMR CNRS-UFC 6249 Chrono-Environnement, University of Franche-Comté, Besancon, France

^3^Wildlife pathology unit & National Reference Laboratory for *Echinococcus* spp., ANSES, Nancy Laboratory for Rabies and Wildlife, 54220 Malzéville, France

^4^Pathology department, University Hospital, 25030 Besançon, France

^5^Digestive and abdominal surgery, University Hospital, 25030 Besançon, France

# Equally contributed to the work

fgrenouillet@chu-besancon.fr

**Background:** *Echinococcus granulosus* is now recognized as a complex of at least four distinct species, gathering ten genotypes with more or less host specificities. Among them, *Echinococcus ortleppi* (genotype G5) describe a dog/cattle lifecycle (“Swiss cattle strain”). Its pathogonicity is probably low for humans, with only six *E. ortleppi* cystic echinococcosis (CE) cases reported worldwide. However, we described here first evidence of *E. ortleppi* in France, as well in humans as in cattle.

**Patients & Methods:** Since 2010, human CE fresh specimens obtained through surgical resection were systematically genotyped using PCR-sequencing of mitochondrial genes (COX1, +/− NAD1 and/or ATP6) in NRC-AE. Concomitantly, a nation-wide slaughterhouse survey of CE was performed in France in 2012. Cysts observed during meat inspection were systematically sampled and stored for further identification/genotyping using same molecular biology tools.

**Results:** Two human cases and seven cattle CE cases due to *E. ortleppi* were identified in France in 2011–2012. *In* Humans: a 63 year-old male originating from Jura, Eastern France, presented with two liver cysts in 2011. Epidemiological questioning allowed us to hypothesize contamination occurred around 10 years before, due to the dog of its son, living in a cattle middle mountain area (Haute-Savoie). A 39 year-old salt marsh-working female, originating from Vendée, Western France presented with typical unique liver cyst. She had lived for twenty years near farmers, with many stray dogs and lack of quick carcass elimination of dead cattle. *In Cattle*: all were lung cysts. Six out of seven exhibited fertile cysts with numerous protoscoleces. The mean age of the animals was ten years (3–14 years). All infected animals were distributed in two separate foci: one in the center (*n* = 4) and one in the south-west (*n* = 3) of the country.

**Discussion**: Here, we described first evidence of autochtonous *E. ortleppi* infections in France, as well in humans as in cattle, with at least four distinct spatial distributions. Lack of travels reporting by patients and close proximity of cattle and dogs argue for autochthonous infection of both human cases. Although experts have suggested that *E. ortleppi* may become extinct in Europe, our report highlights the need for enhancing the national surveillance concerning *E.granulosus sensu lato* in animals as well in humans.

##### P-31. Genotyping of *Echinococcus granulosus* from formalin fixed-paraffin embedded tissues in Tunisia

Hizem Amani^1^, M’rad Selim^1^, Oudni-M’rad Myriam^1^, Mestiri Sara^3^, Mezhoud Habib^1^, Zakhama Abdelfattah^2^, Mokni Moncef^3^, Babba Hamouda^1,4^

^1^Laboratoire de Parasitologie-Mycologie Médicale et Moléculaire (LP3M), LR 12ES08, Faculté de Pharmacie, Université de Monastir, Monastir, Tunisie

^2^Laboratoire d’Anatomie et de Cytologie Pathologiques, E.P.S Fattouma Bourguiba, Monastir, Tunisie

^3^Laboratoire d’Anatomie et de Cytologie Pathologiques, E.P.S Farhat Hached, Sousse, Tunisie

^4^Laboratoire B, Centre de maternité EPS, Fattouma Bourguiba, Monastir, Tunisie

selim.mrad@gnet.tn

**Background:** Genotyping of *Echinococcus granulosus* requires collection and delivery of fresh cysts to laboratory which represents a constraint during investigations carried on remote areas. This study aims to exploit Formal Fixed-Paraffin Embedded Tissues (FF-PETs) in order to identify *E. granulosus* genotypes in Tunisia. This would facilitate samples’ collection step and allow the exploitation of rare and atypical localizations of hydatid cysts.

**Methods:** Paraffin blocks (FF-PETs) were collected in the departments of Anatomy-Pathology at the University Hospital Fattouma Bourguiba of Monastir and at the University Hospital Farhat Hached of Sousse, in Tunisia. Genotyping of 44 samples was performed with a PCR using primers specific to genotype G1 (predominant genotype in Tunisia) which belongs to the specie *E. granulosus* sensu stricto.

**Results:** Our results showed that the target sequence was amplified in all analyzed samples. A difference in the bands’ intensity, obtained on 1.5% agarose gel, was noticed for some isolates.

**Discussion/Conclusion:** We conclude that the infestation of patients was caused by genotype G1 which corroborates our previous results confirming that this genotype predominates in Tunisia. The difference of amplified bands’ intensity is owed to a difference in the amount of DNA extracted from sections of each sample. This amount depends on the size of tissue initially embedded in paraffin and on the abundance of parasitic elements present in these sections, as well.

The use of FF-PETs allowed us to genotype hydatid cysts with rare localizations (brain, heart, submandibular region, spine…) which could not be feasible with classic sampling method. In addition, the exploration of remote areas became possible by using FF-PETs as parasitic material.

##### P-32. Identification of *Echinococcus granulosus* species and case distribution of hydatid cysts in children in Tunisia

M’rad Selim^1^, Oudni-M’rad Myriam^1^, Chaabane-Bennaoues Raja^1^, Hizem Ameni^1^, Bannour-Ben Abdeljelil Abir^1^, Ksia Amine^2^, Lamiri Rachida^2^, Mekki Mongi^2^, Nouri Abdellatif^2^, Mezhoud Habib^1^, Babba Hamouda^1,3^

^1^Laboratoire de Parasitologie-Mycologie Médicale et Moléculaire (LP3M) LR12ES08, Faculté de Pharmacie, Université de Monastir, Monastir, Tunisie

^2^Service de Chirurgie Pédiatrique, Centre de maternité, E.P.S Fattouma Bourguiba, Monastir, Tunisie

^3^Laboratoire B, Centre de maternité, E.P.S Fattouma Bourguiba, Monastir, Tunisie.

selim.mrad@gnet.tn; hamouda.babba@gnet.tn

**Background:** Tunisia is one of the most endemic areas among the Mediterranean countries for cystic echinococcosis. The surgical incidence averages 15/100 000 inhabitants per year and the annual disease cost is estimated approximately to US$ 15 millions. Children are often more vulnerable and may be affected at any age.

The present study is a 13-years (1999–2012) analysis of children hydatidosis in Tunisia. Its purpose was to identify the *Echinococcus granulosus* genotypes responsible of the children disease and to analyze the distribution and the fertility of the hydatid cysts in function of the age and the sex of patients.

**Methods:** 331 cysts coming from 276 children aged 2–16 years operated on at Monastir teaching hospital were analyzed. Identification of strains was carried out by PCR-RFLP of the DNA ITS1 fragment and mitochondrial cytochrome C oxidase gene sequencing. For each cyst, the localization and the fertility of the metacestode as well as age, sex and origin of the patient are listed.

**Results:** Children’s infection was more frequent in the male than in the female sex. The lung was the primary localization of cyst followed by the liver. The most frequent species associated with hydatidosis was *E. granulosus* sensu stricto (G1 genotype). For two children, the G3 genotype (*E. granulosus* s.s.) and the G6 genotype (*E. canadensis)* were observed for the first time in Tunisia. Thus, *E. canadensis* is of greater public health significance than previously believed. The fertility of the cyst was independent of its site or its size and no incidence of age of children was detected.

**Discussion/Conclusion:** Hydatidosis remains a serious problem of public health in Tunisia. *E. granulosus* sensu stricto G1 genotype remains the predominant genotype in our country.

##### P-33. *Echinococcus granulosus* G1 genotype in three hosts (sheep, cattle and man) in Tunisia: same or several?

Oudni-M’rad Myriam^1^, Cabaret Jacques^2^, M’rad Selim^1^, Mekki Mongi^3^, Belguith Mohsen^3^, Sayadi Taoufik^4^, Nouri Abdellatif^3^, Mezhoud Habib^1^, Babba Hamouda^1,5^

^1^Laboratoire de Parasitologie-Mycologie Médicale et Moléculaire (LP3M), Faculté de Pharmacie, Université de Monastir, Monastir, Tunisie

^2^Infectiologie animale et santé publique, INRA, Nouzily, France

^3^Service de Chirurgie pédiatrique, E.P.S Fattouma Bourguiba, Monastir, Tunisie

^4^Abattoir central, Sousse, Tunisie

^5^Laboratoire B, Centre de Maternité E.P.S. Fattouma Bourguiba, Monastir, Tunisie.

myriam.mrad@gnet.tn; jcabaret@tours.inra.fr; hamouda.babba@gnet.tn

**Background:** In Tunisia, *E. granulosus* G1 genotype is the most frequent genotype associated with human, bovine and ovine cystic echinococcosis and is essentially located in the liver and the lung. The existence of genetic and phenotypic variants inside this genotype has been previously shown.

The aim of the present study is to extend the genetic identification of the common G1 genotype in Tunisia in order to determine if it is:

- The « same » G1 genotype in sheep, cattle and human. Is it host species dependent?
- The « same » G1 genotype ovine strain in the lung and the liver. Is it organ dependent?

**Methods:** The G1 genotype identification was based on CO1 mitochondrial DNA. Hundred and four hydatid cysts (sheep, human and cattle) were studied using isoenzymes (DIA, MPI, EST, GPI, MDH, PGM, and PEP) and nuclear DNA of two genes coding for cytosolic MDH (Ag4) and a Calcium linkage protein (Ag6).

The electrophoretic data were interpreted in terms of Fst and Cavalli-Sforza genetic distances. In the three host species, lungs and liver isolates were differentiated since they may originate from different lineages. Reticulograms (based on distances) were constructed using split decompositions, or neighbour-net (Softwares: Splistree and T-Rex).

**Results:** The G1 from the same host were more alike than those from different host species. The human G1 isolates are in intermediary between sheep and cattle isolates, whereas cattle and sheep isolates are fairly different. The human liver samples were more related to sheep whereas lung samples were more related to cattle isolates.

Human infection may result from parasite originating from sheep and cattle isolates although sheep being the main recorded isolate in human infection. The reticulated relationship between the liver or the lung human samples and isolates from bovine lung, is indicative of recombination (sexual reproduction) or lateral genetic transfer.

**Discussion/Conclusion:** Intraspecific evolution cannot always be represented by a bifurcated tree. Evolutionary processes commonly acting at the population level, such as recombination and hybridisation between lineages, generate reticulate relationships within the population. The standard population genetics of worms such as *E. granulosus* is hazardous due to violations of Hardy-Weinberg laws regarding size of sample and cohort synchrony among others. Since the relationships are intraspecific we will use preferably network rather than tree constructions.

##### P-34. Epidemiological and clinical research on spreading of cystic echinococcosis in part of South Central Bulgaria

Muhtarov Marin^1^, Rainova Iskra^2^, Jordanova Diana^2^, Marinova Irina^2^

^1^Gastroenterology Ward, Multi-Profile Hospital for Active Treatment “Kardzhali”, Kardzhali, Bulgaria

^2^Department of Parasitology and Tropical Medicine, National Center of Infectious and Parasitic Diseases, Sofia, Bulgaria

mukhtarov@abv.bg

**Background:** Cystic echinococcosis is one of the widespread parasitic zoonoses which is severely harmful to both humans and animals. From the EU countries, Bulgaria has the highest rate in morbidity of hydatid disease (4.37 per 100 000 in 2012). Since the registered cases are distributed unevenly in different regions of the country the aim of our study was to obtain epidemiological and clinical data of cystic echinococcosis in humans for three Bulgarian regions.

**Methods:** We conducted a retrospective study on 282 patients with human cystic echinococcosis during the 2008–2013 period in three regions located in the southern central part of Bulgaria – Kardzhali, Haskovo and Smolyan. Two hundred and fifty two of investigated patients were confirmed at surgery, or PAIR, and 30 were probable (clinical history, epidemiological history, imaging findings and serology positive for CE). The number of male and female patients was 128 (45.4%) and 154 (54.6%) respectively which were distributed in five age groups. Studied patients were asked to complete a questionnaire regarding epidemiological aspects of hydatidosis. Ultrasound examinations were used for determination and characterization of cysts location and size.

**Results:** The percentage distribution of patients depending on gender showed a significantly higher incidence of this disease in women, compared to men. The highest incidence rate was observed in the 41–65 age group followed by the age groups of 20–40 years old and that of 65 years old. Localization of the cysts at liver was found in 237 of patients after ultrasound examinations and significantly higher percent of patients (80.7%) were with damage of the right hepatic lobe. The largest number of patients was with cyst size 5–10 cm (55.4%) compared to those with size of <5 cm and >10 cm. The percent of patients with primary echinococcosis was significantly higher (79.6) than those with relapses.

**Conclusion:** Our observations after performing the retrospective study on diagnosed patients with echinococcosis in some regions of Bulgaria showed a decrease of the incidence rate for Kardzhali region in 2012 compared to 2008 – from 16.02 to 13.88/100000 and no change regarding Haskovo region (from 6.56 to 6.62/100000), but both values were higher than the national average – 4.37/100000. The probable cause is the presence of a lot of private farms where home slaughter is common and a lot of domestic and stray dogs. On the contrary, significant decreasing of incidence rate from 5.53 to 1.68/100000 was observed for the studied period in Smolyan region.

In order to clarify the actual prevalence of cystic echinococcosis in these regions is necessary to carry out mass screening studies using ultrasonography for the estimation of undiagnosed or asymptomatic cases.

##### P-35. Hydatid disease, a zoonotic threat in Bangladesh; overview on current status and control strategies

Rahman Moizur^1^, Azad Thoufic Anam^2^, Siddiki Amam Zonaed^3^

^1^Department of Animal Husbandry and Veterinary Science, University of Rajshahi, Rajshahi, Bangladesh

^2^Department of Pathology and Parasitology, Chittagong Veterinary and Animal Sciences University, Chittagong, Bangladesh

moizur@gmail.com; thouficdvm305@gmail.com; zsiddiki@gmail.com

**Background:** In Bangladesh, zoonotic diseases are common because of the close proximity of animals to people. Hydatidosis is an important zoonosis caused by metacestode (hydatid cyst) of the dog worm *Echinococcus granulosus*. The parasite utilizes dogs and other canids as definitive hosts whereas many herbivorous and omnivorous species act as intermediate hosts. Since the definitive and intermediate hosts of the *Echinococcus* are abundant in Bangladesh, the magnitude of *Echinococcus granulosus* and hydatid cyst infection is very high.

**Methods:** This study was carried out to understand the current status and control strategies of hydatid disease in Bangladesh. Current status was determined by reviewing the available research article as well as by observation on status of infection in slaughtered domesticated animals. The control strategies were observed in consideration to current activities of public health and livestock department.

**Results:** The traditional rearing of domesticated livestock in scavenging condition and slaughtering of food animals along the roadsides and/or open market provide ample opportunity to complete the *Echinococcus* life cycle. The lack of public attention in slaughterhouse, unhygienic livelihood of poor people’s and interdependence with animals are responsible for widespread hydatidosis in human and animals. There is no exact data about the prevalence of human cases of hydatid disease in Bangladesh but it is not uncommon. A very little study has been conducted on the exact occurrence of hydatidosis in domestic/wild animals of Bangladesh. Fecal examination of stray and house dogs showed high infection levels (50.65%) with *Echinococcus granulosus*. Cystic echinococcosis was recorded in sheep (52.11%), buffaloes (36.11%), cattle (30.62%) and goats (14.73%). Currently, no sophisticated and effective control strategy against hydatidosis/echinococcosis is prevailing in Bangladesh. WHO and CDC are trying to initiate a collaborative control strategy with ICDDR-B (International Centre for Diarrheal Disease Research, Bangladesh), IEDCR (Institute of Epidemiology, Disease Control and Research), public health and livestock sector of Bangladesh, to develop a sophisticated surveillance system and control strategies in order to reduce the risk of hydatidosis/echinococcosis.

**Discussion/Conclusion:** This study focused on identifying the factors behind the prevalent of hydatidosis/echinococcosis in Bangladesh, determination of available risk factors and discuss about future/upcoming control and prevention measures. Advances in knowledge and development/design of new control tools for hydatid disease provide an excellent prospect for improved control programs.

##### P-36. General consideration on control measures used in the semi-nomadic communities in Western China

Zhang Zhuangzhi, Shi Baoxin, Zhang Xu, Zhao Li, Wang Jincheng, Zhang Wenbao

Veterinary Research Institute, Xinjiang Academy of Animal Science, Urumqi, Xinjiang 830000, China

wenbaozhang2013@163.com

**Background:** In the last 20 years, the epidemiological situation of cystic echinococcosis in China has been changed. The high prevalence of the disease in sheep before 1990’s in some areas such as some counties in Xinjiang, Gansu Province and Inner Mongolia has been dropped to low levels due mainly to the changes in economic and style of animal production, and control program processed in these areas. A monthly deworming *Echinococcus* parasite in dogs with praziquantel (PZQ) has been a key measure of control program used in western China. However, the approach has been difficult in the local semi-nomadic pastoral communities. How to treat dogs with PZQ and who give the PZQ medicine to dogs are two key questions for control program in the pastoral semi-nomadic areas.

**Methods:** In Yuming County, Xinjiang, with all sheep rose in traditional semi-nomadic ways, we initially copied the control measures used in Hutubi County, in which we selected a villager in charging of hydatid disease control in each of the villages to dose all the registered dogs with PZQ. A survey after two years of control in Yuming County showed that the prevalence in one-year old sheep was still high (35.7%) and 10.8% of dogs were found copro-antigens positive compared to 21.5% of these dogs copro-antigen positive before the commence of the control study. We then changed to dose dogs by vets in April, May, September and October during “vaccination and treatment season” of animals in “Spring and Autumn pasture” with the rest 8 doses given by the dog owners, who were informed by the vets through mobile during summer and winter seasons in the remote pasture areas. Results: The change of management had increased the control efficacy in the last three years in term of sheep and dog infection with *E. granulosus* dropped to very low rate at 4.9% and 0.9% respectively.

**Discussion/Conclusion:** it is difficult to control cystic echinococcosis in semi-nomadic pastoral communities. Education for dog owners is important. Involvement of dog owners and vets in dog treatment in semi-nomadic communities is a practical and efficient way to delivery of PZQ to dogs.

##### P-37. Geographical information systems: a valid tool to study the epidemiology of cystic echinococcosis

Rinaldi Laura^1^, Maurelli Maria Paola^1^, Musella Vincenzo^2^, Bosco Antonio^1^, Alfano Settimia^3^, Galdiero Massimiliano^3^, Cringoli Giuseppe^1^

^1^Unit of Parasitology and Parasitic Diseases, Department of Veterinary Medicine and Animal Productions, University of Naples Federico II & Regional Center for Monitoring Parasitic Infections of Campania Region (CREMOPAR), Naples, Italy

^2^Department of Health Sciences, University Magna Graecia of Catanzaro, Catanzaro, Italy

^3^Department of Experimental Medicine, Second University of Naples, Naples, Italy

lrinaldi@unina.it; settimia.alfano@yahoo.it

**Background:** Cystic echinococcosis (CE) is a parasitic zoonosis caused by the larval stages of the cestode *Echinococcus granulosus*. The aim of this work was to study the distribution and epidemiology of CE in water buffaloes, cattle and sheep bred in the Campania region (southern Italy) using Geographical Information Systems (GIS), in order to better understand the chain of transmission of *E. granulosus*.

**Materials and methods:** To this ends, two abattoir surveys on CE were performed: the first on individual animals (buffaloes, cattle and sheep); the second, on buffaloes and cattle farms. Moreover, in order to display the spatial distribution of CE in cattle and water buffalo farms, as well as the spatial distribution of sheep farms present in the study area, a point map was drawn within the GIS.

**Results:** The results of the two abattoir surveys showed a prevalence of CE in individual cattle and water buffaloes of 10.4% and 10.5%, respectively, whilst in sheep it was of 31.2%. In cattle and buffalo farms the overall CE prevalence was 23.9%.

The elaboration of the data with GIS showed a close proximity of the bovine and/or water buffalo CE positive farms with the ovine farms present in the study area, thus giving important information on the transmission cycles of CE.

**Discussion:** The sheep and free ranging canids could have a key role in the transmission of CE in Campania region. In fact, cattle and water buffaloes are slaughtered only in large, modern and efficient abattoirs, where the offal is destroyed and the presence of canids is strictly forbidden. For these reasons, it is highly unlikely that dogs or other canids may have the opportunity to feed on cattle and water buffalo carcasses. The sheep, instead, are often slaughtered at home and the carcasses of died sheep are generally left on pastures. Therefore, it is likely that free-ranging canids became infected by CE when feeding on sheep carcasses in the sheep farms, and then went on to shed infectious eggs in proximate cattle/buffalo farms.

In conclusion, the use of GIS is a novel and important approach to further understanding of the epidemiology and control of CE.

##### P-38. Epidemiological and serological profile of cystic echinococcosis cases diagnosed in the parasitology laboratory of Charles Nicolle hospital, Tunis

Trabelsi Sonia, Bouchekoua Myriam, Aloui Dorsaf, Khaled Samira

Parasitology Laboratory, Charles Nicolle Hospital, Tunis, Tunisia

samira.khaled@rns.tn

**Objective:** Cystic echinococcosis (CE) is caused by metacestodes of *Echinococcus granulosus*, which is one of the endemic zoonotic diseases in humans in Tunisia. The aim of this retrospective study was to assess the epidemiological profile and serological results of patients affected by CE.

**Methods:** During the year 2013, 274 patients with probable CE were referred to the parasitology and mycology laboratory of Charles Nicolle hospital in Tunis, Tunisia. Specific anti-*Echinococcus granulosus* antibodies were tested by enzyme-linked immunoassay (EIA). Indirect hemagglutination (IH) was used when the result was at the borderline.

**Results:** The EIA test was positive in 44.89% (123 patients), negative in 53.65% (147 patients) and borderline in 1.46% (4 patients). In this last case, IH was negative and the results were concluded as negative. Among the 123 cases, 86 (70%) were female and 37 (30%) were male. Their mean age was 49.35 years. Most of them (49.5%) were from the north-west of the country. The contact with dogs and herbivore breeding were reported respectively in 79.6% and 73.6% of cases. The principal site of the hydatic disease was the liver (85.08%).

**Conclusion:** Generally, the epidemiological characteristics fit with the other Tunisian data. The transmission seems as strong as in the past, in spite of a much better social educational level, specialy through media programs.

##### P-39. Estimating the incidence of cystic echinococcosis in France using the French nationwide hospital medical information database

Van Cauteren Dieter^1^, Grenouillet Frédéric^2^, de Valk Henriette^1^

^1^Institut de Veille Sanitaire (InVS), Saint-Maurice, France

^2^Centre National de Référence Echinococcose alvéolaire, University Hospital, Besançon, France

d.vancauteren@invs.sante.fr; http://cnr-echino-alveolaire-ccoms.univ-fcomte.fr; www.invs.sante.fr

**Background:** In France, little is known about the incidence and disease burden of cystic echinococcosis (CE). We analyzed the national French hospital discharge database (PMSI) to describe the demographic characteristics of CE cases and to estimate the incidence from 2005 to 2012.

**Methods:** The PMSI is a national database which describes public and private hospital activity in France. For each hospitalization, codes for the discharge diagnoses (main, related and secondary) are included in the database according to the International Classification of Diseases codes, 10th revision (ICD-10). Demographical data (age, sex), length of stay, residential location, and death occurring during hospitalization are also recorded. We selected in the PMSI all records between 1 January 2004 and 31 December 2012 containing codes for CE (B67.0 to B67.4) or alveolar echinococcosis (B67.5 to B67.7).

A hospitalization for CE was defined as a hospitalization for which at least one of the ICD-10 codes for CE was listed as a discharge diagnosis (main, related, or secondary). Patients who also had a hospital record of alveolar echinococcosis were excluded from the analysis. Patients with multiple hospitalizations were detected using their unique identifier and only data from the first hospitalization was taken into account.

**Results:** Among 1263 identified hospital stays linked to CE, 886 incident cases were identified in mainland France between 2005 and 2012. There were 447 males and 439 females (sex ratio: 1.0) with a mean age of 48 years (median: 46 years, range: <1–100 years). The mean duration of hospitalization was 10 days (median 8 days, range 0–81 days). The annual incidence rate (AIR) was stable from 2005 to 2012 around 0.18 cases per 100,000 inhabitants (mean of 111 new cases per year). The number of cases and AIR per region of residence varied respectively from 2 to 173 and the AIR from 0.01 to 1.03 cases per 100,000 over the 8-year period. It was highest in Corsica (1.03 cases/10^5^), followed by Alsace (0.36 cases/10^5^), Provence-Alpes-Côte-d’Azur (0.34 cases/10^5^) and Franche-Comté (0.33 cases/10^5^).

**Discussion/Conclusion:** The incidence of hospitalized cases of CE was stable between 2005 and 2012 with a mean of 111 new cases and 158 hospitalizations linked to CE every year in France.

The results of this study confirm the specific epidemiological situation of the Corsica region which is historically an endemic area of CE in France. The incidence rate in Corsica is more than five times higher than in the rest of France. The proportion of autochthonous cases cannot be determined through the PMSI, and needs further study.

Despite the limitations linked to the PMSI database it allows a description of CE cases and an overview of CE incidence trends over the past 8 years in France.

##### P-40. Human hydatidosis in South Bulgaria – Plovdiv and Pazardjik districts (2009–2013)

Vuchev Dimitar^1^, Popova-Daskalova Galia^1^, Stancheva Galina^2^

^1^Chair of Infectious diseases, Parasitology and Tropical medicine, Medical University, Plovdiv, Bulgaria

^2^Regional Health Inspection (RHI), Plovdiv, Bulgaria

dvutchev@yahoo.com; rzipd@plov.net

**Background:** Although almost two-fold reduction in the morbidity of human hydatidosis over the past decade – from 8,32%ooo (2002) to 4,09%ooo (2011) as a result of the National Programme for Control of echinococcosis in humans and animals conducted in the period 2004–2008, it remains the most significant parasitic disease in medical and social aspect for Bulgaria (1). The objective of this study is to represent the main epidemiological characteristics indicating the spread and distribution of hydatidosis by gender, age, residence and organ location in Plovdiv and Pazardzhik regions after the discontinuance of the National Programme against echinococcosis.

**Methods:** Data on officially registered cases of echinococcosis in Plovdiv and Pazardzhik districts in the period 2009–2013 were investigated. Information sources were questionnaires for epidemiological study of patients with hydatid disease, the annual reports of departments of parasitology at RHI – Pazardzhik and RHI – Plovdiv and personal clinical and epidemiological observations.

**Results:** In Plovdiv and Pazardzjik districts – a large part of Central South Bulgaria, were registered 175 cases of echinococcosis (respectively 101 and 74 cases) for a 5-year period (2009–2013) after the termination of the National Programme. The average annual morbidity rate for both areas was 3,58%ooo. Of all diagnosed children and young people under 19 years were 48 (27,43%). Zonal distribution revealed that cases were registered in almost all municipalities of these regions. The average rate of morbidity of urban and rural population is in similar values. Among the patients, male were predominant (58,86% vs. 41,14%). Hydatid cysts located most frequently in the liver – 76,73%, followed by lung – 15,09%. Simultaneously the two organs were affected in 2,52% of the patients, another less common location (spleen, brain, retroperitoneal space, vertebra, myocardium) was recorded at 5,66%.

**Conclusion:** The morbidity of echinococcosis gradually increased in investigated districts from 1,93%ooo (2010) to 4,63%ooo (2013) as well as in the country –4,37%ooo (2012) (2). The data show persistence of disseminated active parasitic foci of hydatidosis. It indicates the need for further activities to control echinococcosis in Bulgaria, including the collection of stray dogs. Human hydatidosis is still an important health problem for Plovdiv and Pazardzhik regions and the entire country.

##### P-41. Genetic characterisation of *Echinococcus granulosus s.l.* isolates from patients treated in a German treatment centre for echinococcosis

Wagner Sarah^1^, Wassermann Marion^1^, Ebi Dennis^1^, Romig Thomas^1^, Stojkovic Marija^2^

^1^Parasitology Unit, University of Hohenheim, Stuttgart, Germany

^2^Dept. of Clinical Tropical Medicine, University of Heidelberg, Heidelberg, Germany

sarah.wagner@uni-hohenheim.de

**Background:** Cystic echinococcosis can be caused by a diverse array of cryptic species and genotypes within the *E. granulosus* cluster. While a large body of literature exists on clinical aspects of CE as a whole, there are only few (and often anecdotal) data on CE caused by those various taxa (and the possible differences between them), as well as their geographical spread.

**Methods:** To obtain additional data on this, we analysed 36 isolates of *E. granulosus* s.l. from patients, which had been surgically treated at the Section of Clinical Tropical Medicine of the University Hospital Heidelberg from 1999 to 2011. The patients originated from 19 different countries of Eastern Europe (10), central and southern Europe (5), western Asia (16), and northern Africa (5). As a marker for genetic identity we analysed the complete mitochondrial gene *cox1* (1609 bp).

**Results:** All samples except two were identified as *E. granulosus* s.s., two (one from a patient originating from Romania and one from a patient originating from Iran) belonged to *E. canadensis* G6/7. In addition, we analysed genetic variants (haplotypes) of our samples and present their geographical distribution. Within the 34 samples of *E. granulosus* s.s. 24 different haplotypes could be found.

**Conclusion:** Our data confirm the predominance of *E. granulosus* s.s. in patients from all geographical areas. The high intraspecific diversity of *E. granulosus* s.s. was confirmed for patients from western Asia, to a lesser degree for patients from other origins.

##### P-42. Analysis of economic burden for patients with cystic echinococcosis in five hospitals in Northwest China

Wang Le, Wen Hao, Feng Xiaohui, Jiang Xiaomijng, Duan Xinyu

First Affiliated Hospital of Xinjiang Medical University, Urumqi 830000, Xinjiang, China

wangle19850408@sina.com

**Background and aims:** China was one of the most important endemic regions for CE; 28 of its 34 provinces had reported cases of CE, covering 44.6% of the total land area in China, with nearly 98% of cases in Xinjiang, Qinghai, Gansu, Ningxia, Tibet, Inner Mongolia, Shanxi and Sichuan. This study was to evaluate the direct and indirect economic burden of echinococcosis disease on patients admitted to five specialist hydatid hospitals in Xinjiang, PR China.

**Materials and methods:** 2018 patients with CE were admitted to five specialist hydatid hospitals in Xinjiang, PR China during the period 2004–2008. Both direct medical cost and direct non-medical cost constituted direct economic burden. Direct medical costs are comprised of the cost of hospitalization and direct non-medical cost was evaluated by the food expenditure of patients, which was calculated on the basis of per capita consumption expenditure and the Engel coefficient between 2004 and 2008. The indirect economic burden was analysed by the human capital method combined with DALYs. The formula was indirect economic burden = per capita GNP × DALY × productivity weight. The formula for DALYs lost by an individual was:

$$-\frac{{\mathrm{DCe}}^{-\beta \alpha }}{{\left(\beta +\gamma \right)}^{2}}\left\{e^{-\left(\beta +\gamma \right)L}\left[1+(\beta +\gamma )(L+\alpha )\right]-\left[1+(\beta +\gamma )\alpha \right]\right\}$$

**Results:** The per-person direct medical cost was US$1493.12 (95% CI 1438.43–1547.80) and the per-person direct non-medical cost was US$19.67. The indirect economic cost was US$ 1435.96 per person, and the disability adjusted life-years (DALY) lost was approximately 1.03 DALY/person.

**Conclusions:** This study is the first to combine the human capital method with DALYs to analyse the indirect CE economic burden in northwest China. Factors such as age, occupation and hospital level should be considered when developing polices to reduce the economic burden of CE.

(Published in Transactions of the Royal Society of Tropical Medicine and Hygiene 2012; 106: 743–748)

##### P-43. Intracardiac cystic echinococcosis in a pig: a case report

Scala Antonio^1^, Baule Antonio^2^, Marrosu Raffaele^2^, Varcasia Antonio^1^, Dore Francesco^1^, Tosciri Gabriele^1^, Pipia Anna Paola^1^, Sanna Giuliana^1^, Tamponi Claudia^1^

^1^Dipartimento di Medicina Veterinaria, Università degli Studi di Sassari, Sassari, Italy

^2^ASL N.1 Sassari, Italy

scala@uniss.it

**Background:** Cystic echinococcosis (CE) caused by *Echinococcus granulosus* is a zoonotic disease of worldwide importance that is widespread on the island of Sardinia (Italy). Livestock breeding occurs extensively in Sardinia, wherein some 3 million sheep are present, representing one third of all sheep in Italy. Less information is available about the epidemiology of CE in pigs in Sardinia, because the infection can be found only in animals raised free in the fields, which are often illegally butchered in the farms. The prevalence of CE in farm-raised pigs has been determined to be 9.4% and two strains, G1 and G7 have been isolated in these ungulates.

Pigs could be massively infected when they are free ranging, as they are occasionally coprophagous. When this happens, not only liver and lungs are parasitized but also other locations like spleen, kidney, heart and skeleton muscles.

**Methods:** Herein, we report the case of a 2-year-old female pig which was slaughtered for family consumption.

Veterinary inspection revealed Hydatid disease with two cysts in the liver, and another one big hydatid located in the left ventricle of the heart (5 cm × 2,5 cm; 26 ml volume).

Fertility was evaluated from each cyst, and laminar layers and protoscoleces were removed and stored at −20 °C. DNA was extracted from 28 samples of hydatid material using a commercial kit (Roche DNA template extraction kit).

Sequencing reactions for strain typing were undertaken on PCR products as described by Bowles J, McManus DP (1993) for NADH and COI mitochondrial genes.

**Results:** Light microscopy allow to detect the viability of all hydatids and strain typing that the parasite was G1 strain or *Echinococcus granulosus sensu stricto*. The heart localization was pretty interesting as develop from the ventricle side but the cyst growing invades the whole ventricular space, finally taking its shape.

**Discussion:** It so surprising as the pig had no apparent symptoms related, even if in its last months it lived confined in a box. Anyway if it had not been slaughtered, it would be probably died for cyst rupture and thromboembolism in systemic or pulmonary circulation.

##### P-44. *Echinococcus equinus* and other taeniid cestodes in wildlife of the Etosha national park, Namibia

Wassermann Marion^1^, Aschenborn Ortwin^2^, Fellhauer Julia^3^, Mackenstedt Ute^1^, Romig Thomas^1^

^1^Dept. of Parasitology, University of Hohenheim, Stuttgart, Germany

^2^Etosha Ecological Institute, Okaukuejo, Etosha, Namibia

^3^Tierärztliche Hochschule Hannover, Hannover, Germany

huettner@uni-hohenheim.de

**Background:** Various species of *Echinococcus* and *Taenia* had been described in the past from wild mammals of southern Africa. No molecular identification had been done to date, which is especially relevant due to the known diversity within *E. granulosus* sensu lato.

**Methods:** A preliminary survey for taeniid cestodes was done in Etosha National Park, Namibia, from August to October 2012. Faecal samples were obtained from 34 individual carnivores (lions, leopards, cheetahs, caracals, spotted hyenas and silverbacked jackals), which were temporarily kept in enclosures in the course of a relocation program. Carcasses of 18 culled herbivores (12 plains zebras and 6 oryx), were examined for larval taeniids. Single eggs and metacestode tissue were lysed and identified by sequencing of the mitochondrial *nad1* gene and comparison with Genbank entries.

**Results:** Several haplotypes of *Echinococcus equinus* were found in lions (4 of 6), jackals (2 of 7) and zebras (11 of 12). The frequency of this parasite in the absence of domestic dogs strongly indicates its transmission in a wildlife cycle. Further, a variety of sequences were obtained from eggs and cysticerci from lions, cheetahs, caracals, hyenas and oryx, which most closely clustered with species of *Taenia* and *Hydatigera*. Only two of them, of lion origin, could be allocated to *H. taeniaeformis* and *T. regis*, respectively. *T. regis* cysticerci were also found in oryx. The remaining sequences were tentatively assigned to 12 distinct *Taenia* taxa, but could not be further identified due to non-availability of intact adult worms, and the lack of Genbank data for most African *Taenia* species.

**Discussion/Conclusion:** For the first time, lions are confirmed as suitable hosts for *Echinococcus* species other than *E. felidis*, and the first wildlife cycle of *E. equinus* is described. The diversity of other taeniids in wild mammals was found to be far higher than expected.

##### P-45. Veterinary management of Alveolar Echinococcosis in zoo gorillas

Wenker Christian, Hoby Stefan

Zoo Basel, Basel, Switzerland

wenker@zoobasel.ch

**Background:** Between 1999 and 2013 alveolar echinococcosis (AE) was diagnosed in seven lowland gorillas (*Gorilla g. gorilla*), originating from Zoo Basel in Switzerland. Four gorillas died and AE was confirmed at necropsy. Three clinical cases were diagnosed in 2007 (5-yr-old male gorilla) and in 2010 (21-yr-old male silverback gorilla and 42-yr-old female gorilla, respectively) during routine health check exams under general anesthesia, using imaging techniques and serology. These individuals are continuously treated with albendazole (Valbazen 10%, Pfizer AG, CH-8052 Zurich, Switzerland).

**Clinical and epidemiological observations:** Despite therapeutic drug plasma levels, the lesions are slowly progressing and the silverback male gorilla started to show emaciation and phases of lethargy in autumn 2013. Further investigation under general anesthesia revealed hypalbuminemia with hydropericard, hydrothorax, and ascites. No cases have occurred in the Sumatran orangutans (*Pongo abelii*), but one chimpanzee (*Pan troglodytes*) has been tested serologically positive repeatedly in 2010 and 2011. All the three species of the great apes are housed under similar conditions and direct contact to the red fox (*Vulpes vulpes*) can be excluded. The incubation period of the disease in humans is 5–15 years and infection may also remain undetected in gorillas for years. It is assumed that food or other material which was taken into the enclosure was contaminated with fox faeces and infected the gorillas. Possible sources were evaluated, but no specific cause was identified. Preventive sanitary measures for the preparation and storage of introduced food and materials including treatment with low heat may help reduce AE infections. A thermal treatment protocol for great ape food is currently investigated at the Zoo Basel in cooperation with the Institute of Parasitology of the University of Zurich.

**Discussion and conclusion:** It is assumed that lowland gorillas run an increased risk of becoming aberrant hosts and of developing AE as compared with other great ape species or humans. It has also been speculated that the immune system of gorillas has not co-evolved with this pathogen because it has never been present in their natural habitat in equatorial Africa. This could explain why the disease occurs earlier and progresses faster, and why albendazole therapy is less effective than in humans. Since highly socialized non-human primates like gorillas are difficult to monitor, and daily treatment and supportive care are limited to oral medication, at least in untrained animals, veterinary care and management remain challenging. Therefore, new preventive and treatment modalities are urgently needed for the highly endangered zoo gorillas in areas where *Echinococcus multilocularis* is present.

## Session 3. New tools for treatment

### 3.A. Innovative interventions

#### State-of-the-art

##### L-05. Non-surgical and non-chemical attempts to treat echinococcosis: do they work?

Tamarozzi Francesca^1^, Vuitton Lucine^2^, Brunetti Enrico^1^, Koch Stéphane^2^

^1^WHO-Collaborating Centre for Clinical Management of Cystic Echinococcosis, Pavia, Italy

^2^WHO-Collaborating Centre for Prevention and Treatment of Human Echinococcosis, Besançon, France

f_tamarozzi@yahoo.com; enrico.brunetti@unipv.it; lvuitton@chu-besancon.fr; skoch@chu-besancon.fr

Cystic (CE) and Alveolar Echinococcosis (AE) are chronic, complex and neglected diseases. Their treatment depends on a number of factors, such as location, size and stage of the cysts/lesions, and availability of therapeutic options. We performed a literature review of non-surgical and non-chemical interventions in CE and AE. This included 1) non-surgical drainage techniques (with a parasite-killing objective); 2) non-conventional procedures of treatment; and 3) non-surgical drainage techniques with a palliative objective.

In CE, although prospective clinical studies are still missing more than 20 years after their introduction in clinical practice, non-surgical drainage techniques with a parasite-killing objective, such as PAIR and its modifications (catheterization, MoCaT) have received clinically documented evidence of efficiency and are now considered to be a useful alternative to conventional surgical and medical treatment. Systematic literature search did not show any attempt of this kind of technique in AE, even experimentally.

Few clinical or experimental attempts have been made to test and/or develop alternative techniques to treat CE or AE. In CE, some of these techniques, like Radiofrequency Thermal Ablation (RTA), after giving some hope to clinicians, have been shelved, while others, such as High Intensity Focused Ultrasound, appear promising but are still in a preclinical phase. In AE, unexpectedly, RTA was never tested, and only experimental work is available on experimental in-vivo conventional radiotherapy, or in-vitro heavy-ion therapy. Clinical use of Gamma knife radiosurgery in a non-operable cerebral AE showed this could be an alternative. Such attempts provide a framework to further study the effect of radiation therapy on *E. multilocularis*.

Finally, non-surgical drainage techniques with a palliative objective may be required in both CE and AE when the disease location makes radical treatment impossible, or as a bridge before a curative surgical procedure in symptomatic patients presenting life-threatening bacterial and/or fungal infection. US- or CT-guided percutaneous biliary drainages, centro-parasitic abscesses drainages, or vascular stenting in hepatic veins were performed successfully, despite a number of adverse events. Recently, they have been progressively replaced by ERCP-associated interventional techniques, to manage biliary fistulas in CE and biliary obstructions in AE. In AE, their efficacy relies on extensive biliary lavage before stent placement, and the insertion of several stents to enlarge the biliary strictures and reduce the number of procedures.

Data obtained from the existing literature suggest that not all non-surgical non-chemical techniques of treatment have been explored for the treatment of echinococcosis, and especially AE. Development of good pre-clinical animal models would allow new attempts using all techniques developed for other indications, including cancer. Best use of PAIR and associated procedures should be assessed using prospective trials. Proper evaluation of the various types of palliative drainage will also determine their best indications and techniques.

**References**

Akhan O, Gumus B, Akinci D, Karcaaltincaba M, Ozmen M. Diagnosis and percutaneous treatment of soft-tissue hydatid cysts. Cardiovasc Intervent Radiol 2007 May-Jun;30 (3):419–25.

Ambregna S, Vuitton L, Koch S, Sulz M C, Chevaux J B, Moradpour D, Bichard P, Prat F, Vanbiervliet G, Kull E, Richou C, Vuitton DA, Bresson-Hadni S. Per endoscopic management of alveolar echinoccosis biliary complications: a european survey. ImE-2014, Besançon: O-28

Bao YX, Zhang YF, Ni YQ, Xie ZR, Qi HZ, Mao R, Yang YG, Wen H. [Effect of 6 MeV radiotherapy on secondary *Echinococcus multilocularis* infection in rats]. Zhongguo Ji Sheng Chong Xue Yu Ji Sheng Chong Bing Za Zhi 2011 Apr 30;29(2):127–9 (in Chinese).

Bastid C, Ayela P, Sahel J. Percutaneous treatment of a complex hydatid cyst of the liver under sonographic control. Report of the first case. Gastroenterol Clin Biol 2005 Feb;29 (2):191–2.

Bresson-Hadni S1, Delabrousse E, Blagosklonov O, Bartholomot B, Koch S, Miguet JP, Mantion GA, Vuitton DA. Imaging aspects and non-surgical interventional treatment in human alveolar echinococcosis. Parasitol Int 2006;55 Suppl:S267–72.

Bret PM, Paliard P, Partensky C, Bretagnolle M, Blanchut P. [Treatment of cholestasis caused by stenosis of the intrahepatic bile ducts in alveolar echinococcosis. Trial of biliary drainage by the percutaneous transhepatic approach]. Gastroenterol Clin Biol 1984 Apr;8 (4):308–13 (in French).

Brunetti E, Filice C. Radiofrequency thermal ablation of echinococcal liver cysts. Lancet 2001 Oct 27;358 (9291):1464.

Brunetti E, Kern P, Vuitton DA; Writing Panel for the WHO-IWGE. Expert consensus for the diagnosis and treatment of cystic and alveolar echinococcosis in humans Acta Trop 2010 Apr;114(1):1–16

Cai H, Chen LL, Ye B, Liu AB, Zhang J, Zhao YF. The destructive effects of high-intensity focused ultrasound on hydatid cysts enhanced by ultrasound contrast agent and superabsorbent polymer alone or in combination. Parasitol Res 2013 Feb;112(2):707–17.

Canyigit M, Gumus M, Cay N, Erol B, Karaoglanoglu M, Akhan O. Refractory cystobiliary fistula secondary to percutaneous treatment of hydatid cyst: treatment with N-butyl 2-cyanoacrylate embolization. Cardiovasc Intervent Radiol 2011 Feb;34 Suppl 2:S266–70.

Du XL, Ma QJ, Wu T, Lu JG, Bao GQ, Chu YK. Treatment of hepatic cysts by B-ultrasound-guided radiofrequency ablation. Hepatobiliary Pancreat Dis Int 2007 Jun;6 (3):330–2.

Dziri C, Haouet K, Fingerhut A, Zaouche A. Management of cystic echinococcosis complications and dissemination: where is the evidence? World J Surg 2009 Jun;33(6):1266–73.

Filice C, Brunetti E. Use of PAIR in human cystic echinococcosis. Acta Trop1997 Apr 1;64 (1–2):95–107.

Gabal AM, Khawaja FI, Mohammad GA. Modified PAIR technique for percutaneous treatment of high-risk hydatid cysts. Cardiovasc Intervent Radiol. 2005 Mar-Apr;28 (2):200–8.

Golemanov B, Grigorov N, Mitova R, Genov J, Vuchev D, Tamarozzi F, Brunetti E. Efficacy and safety of PAIR for cystic echinococcosis: experience on a large series of patients from Bulgaria. Am J Trop Med Hyg 2011 Jan;84 (1):48–51

Haddad MC, Sammak BM, Al-Karawi M. Percutaneous treatment of heterogenous predominantly solid echopattern echinococcal cysts of the liver. Cardiovasc Intervent Radiol 2000 Mar-Apr;23 (2):121–5.

Kovalenko FP, Biriukov IuV, Moiseev VS, Abdrimov EG, Krotov AI. [Effect of low-frequency ultrasound on the viability of embryonal elements of the larvocysts of *Echinococcus granulosus* and *Echinococcus multilocularis* in vitro]. Med Parazitol (Mosk) 1986 Nov-Dec;(6):31–6 (in Russian)

Lamonaca V, Virga A, Minervini MI, Di Stefano R, Provenzani A, Tagliareni P, Fleres G, Luca A, Liu AB, Cai H, Ye B, Chen LL, Wang MY, Zhang J, Zhao YF. The damages of high intensity focused ultrasound to transplanted hydatid cysts in abdominal cavities of rabbits with aids of ultrasound contrast agent and superabsorbent polymer. Parasitol Res 2013 May;112 (5):1865–75.

Liu AB, Cai H, Ye B, Chen LL, Wang MY, Zhang J, Zhao YF. The damages of high intensity focused ultrasound to transplanted hydatid cysts in abdominal cavities of rabbits with aids of ultrasound contrast agent and superabsorbent polymer. Parasitol Res 2013 May;112(5):1865–75.

Marin Gorríz F. [Experimental contribution to radiobiology of the hydatid cyst]. An R Acad Nac Med (Madr) 1976;93(1):117–39 (in Spanish).

Men S, Yücesoy C, Edgüer TR, Hekimoğlu B. Percutaneous treatment of giant abdominal hydatid cysts: long-term results. Surg Endosc 2006 Oct;20 (10):1600–6.

Mohan S, Garg SK, Kathuria M, Baijal SS. Mechanical suction through wide bore catheters for nonsurgical management of Gharbi type III hepatic hydatid cysts. Trop Gastroenterol 2011 Jul-Sep;32 (3):189–95.

Nasseri-Moghaddam S, Abrishami A, Taefi A, Malekzadeh R. Percutaneous needle aspiration, injection, and re-aspiration with or without benzimidazole coverage for uncomplicated hepatic hydatid cysts. Cochrane Database Syst Rev 2011 Jan 19;(1):CD003623. Review.

Nasseri Moghaddam S, Abrishami A, Malekzadeh R. Percutaneous needle aspiration, injection, and reaspiration with or without benzimidazole coverage for uncomplicated hepatic hydatid cysts. Cochrane Database Syst Rev 2006 Apr 19;(2):CD003623. Review. Update in: Cochrane Database Syst Rev. 2011;(1):CD003623. Review.

Neumayr A, Troia G, de Bernardis C, Tamarozzi F, Goblirsch S, Piccoli L, Hatz C, Filice C, Brunetti E. Justified concern or exaggerated fear: the risk of anaphylaxis in percutaneous treatment of cystic echinococcosis – a systematic literature review. PLoS Negl Trop Dis 2011 Jun;5(6):e1154.

Pohle S, Ernst R, MacKenzie C, Spicher M, Romig T, Hemphill A, Gripp S. *Echinococcus multilocularis*: the impact of ionizing radiation on metacestodes. Exp Parasitol 2011 Jan;127(1):127–34.

Ramia JM, Figueras J, De la Plaza R, García-Parreño J. Cysto-biliary communication in liver hydatidosis. Langenbecks Arch Surg 2012 Aug;397(6):881–7.

Rustamov IR, Shishkin VN, Kurbaniiazov ZB, Murtazaev ZI, Akhmedov AZ. [Effectiveness of laser treatment of patients with hepatic echinococcosis]. Klin Khir 1991;(11):66. (in Russian)

Saremi F, McNamara TO. Hydatid cysts of the liver: long-term results of percutaneous treatment using a cutting instrument. AJR Am J Roentgenol 1995 Nov;165(5):1163–7.

Schipper HG, Laméris JS, van Delden OM, Rauws EA, Kager PA.Percutaneous evacuation (PEVAC) of multivesicular echinococcal cysts with or without cystobiliary fistulas which contain non-drainable material: first results of a modified PAIR method. Gut 2002 May;50(5):718–23.

Schmid M, Pendl G, Samonigg H, Ranner G, Eustacchio S, Reisinger EC. Gamma knife radiosurgery and albendazole for cerebral alveolar hydatid disease. Clin Infect Dis1998 Jun;26(6):1379–82.

Schnider P. Gamma knife radiosurgery in cerebral echinococcosis. Clin Infect Dis 1999 Feb;28(2):409.

Ulger S, Barut H, Tunc M, Aydin E, Aydınkarahaliloğlu E, Gokcek A, Karaoğlanoğlu N. Radiation therapy for resistant sternal hydatid disease. Strahlenther Onkol 2013 Jun;189(6):508–9.

Vizzini G, Palazzo U, Gridelli B. Cystic echinococcosis of the liver and lung treated by radiofrequency thermal ablation: an ex-vivo pilot experimental study in animal models. World J Gastroenterol 2009 Jul 14;15(26):3232–9.

Vogel J, Görich J, Kramme E, Merkle E, Sokiranski R, Kern P, Brambs HJ. Alveolar echinococcosis of the liver: percutaneous stent therapy in Budd-Chiari syndrome. Gut 1996 Nov;39(5):762–4.

Zhang J, Ye B, Kong J, Cai H, Zhao Y, Han X, Li F. In vitro protoscolicidal effects of high-intensity focused ultrasound enhanced by a superabsorbent polymer. Parasitol Res 2013 Jan;112(1):385–91.

Zhang YF, Xie ZR, Ni YQ, Mao R, Qi HZ, Yang YG, Jiang T, Bao YX. Curative effect of radiotherapy at various doses on subcutaneous alveolar echinococcosis in rats. Chin Med J (Engl) 2011 Sep;124(18):2845–8.

Zhou X, Zhao Y, Zhou R, Zhang H. Suppression of *E. multilocularis* hydatid cysts after ionizing radiation exposure. PLoS Negl Trop Dis 2013 Oct 24;7(10):e2518.

Zou X, Wang J, Zhao H, Zhang J, Wu W, Ye B. *Echinococcus granulosus*: protoscolicidal effect of high intensity focused ultrasound. Exp Parasitol 2009 Apr;121(4):312–6.

#### Oral communications

##### O-27. Laparoscopic approach for total pericystectomy in treating hepatic cystic echinococcosis

Li Haitao^1,2^, Shao Yingmei^1,2^, Aili Tuergan^1,2^, Zhang Jinhui^3^, Kashif Kafayat^1^, Ma Qinglong^1^, Ran Bo^1^, Wen Hao^1,2^

^1^Hepatobiliary & Hydatid Department, Digestive and Vascular Surgery Centre, First Affiliated Hospital of Xinjiang Medical University, Urumqi, 830011, China

^2^State Key Lab Incubation Base of Xinjiang Major Diseases Research (2010DS890294) and Xinjiang Key Laboratory of Echinococcosis, First Affiliated Hospital of Xinjiang Medical University,Urumqi, 830011, China

^3^Department of Liver and Laparoscopic Surgery, Digestive and Vascular Surgery Centre, First Affiliated Hospital of Xinjiang Medical University, Urumqi, 830011, China

dr.wenhao@163.com

**Background:** With the development of minimally invasive surgery, higher demands have been requested by the patients for non-invasive surgeries. Laparoscopic approach for total pericystectomy has been proposed for treating hepatic cystic echinococcosis (HCE) and has been commonly used in clinical practices. So, we also tried to develop this approach for treating hepatic cystic echinococcosis.

**Methods:** To evaluate the reliability and feasibility of the laparoscopic approach for total pericystectomy in treating hepatic cystic echinococcosis, A retrospective review of the medical records of 22 patients diagnosed with hepatic cyst echinococcosis between June 2009 and June 2013 was conducted at first affiliated hospital of Xinjiang medical university. Seven patients were forced to transfer open surgery (OS) in the course of laparoscopy for operational complexity. Total pericystectomy of hepatic cyst echinococcosis was performed by laparoscopy (LC) in fifteen selected patients. The postoperative items were evaluated.

**Results:** A total of 15 patients were done total pericystectomy of hepatic CE. The average time of surgery was about 174 min (160 ~ 210 min). Bleeding of surgery was about 102.6 ml (80 ~ 200 ml). The mean duration of hospitalization was 7.3 days (6 ~ 15 days). Additional 7 patients were transferred to OS in the course of laparoscopy. For these seven patients, the average duration of surgery was about 177.1 min (150 ~ 230 min). Bleeding of surgery was about 237.1 ml (160 ~ 350 ml), and the mean duration of hospitalization was 10.1 days (8 ~ 15 days).The most frequent postoperative complications were hydrops in the surgical area (2 cases in LC and 3 cases in OS), bile leakage (1 patient in LC group). Recurrence was not seen in any cases in both groups by following 6 ~ 12 months. Total pericystectomy of hepatic cyst echinococcosis was superior to open surgery in selected cases for less bleeding, less mean duration of hospitalization and rapid recovery.

**Conclusions:** Total pericystectomy of hepatic cyst echinococcosis is a safe and effective method for selected patients with unique, small-sized, superficially located cysts.

##### O-28. Per endoscopic management of alveolar echinoccosis biliary complications: a European survey

Ambregna Sylvain^1^, Vuitton Lucine^1^, Koch Stéphane^1^, Sulz Michael Christian^2^, Chevaux Jean Baptiste^3^, Moradpour Darius^4^, Bichard Philippe^5^, Prat Frederic^6^, Vanbiervliet Geoffroy^7^, Kull Eric^8^, Richou Carine^1^, Vuitton Dominique A.^1^, Bresson-Hadni Solange^1^

^1^University Hospital, & WHO-Collaborating Centre for Prevention and Treatment of Human Echinococcosis, Besançon, France

^2^Kantonsspital, St. Gallen, Switzerland

^3^University Hospital, Nancy, France

^4^University Hospital, Lausanne, Switzerland

^5^University Hospital, Geneva, Switzerland

^6^Cochin University Hospital, Paris, France

^7^University Hospital, Nice, France

^8^Metz-Thionville Regional Hospital, Metz, France

WHO-AE-endoscopy@chu-besancon.fr

**Backround:** Cholestasis and cholangitis are common and life-threatening complications of Alveolar Echinococcosis (AE), because of the liver invasion by the metacestode associated with extensive fibro-inflammatory host reaction. Biliary complications are usually treated by surgery or radiological percutaneous biliary drainage. During the last decade the indication of per-endoscopic biliary drainage following endoscopic retrograde cholangio-pancreatography (ERCP) has markedly increased in various types of biliary tree obstructions. AE is an emerging indication in this field; only little is known about its efficacy and safety. Our aim was to collect and analyse the European experience in per-endoscopic management of AE biliary complications.

**Methods:** All European physicians practicing ERCP and/or in charge of AE patients were recruited directly or through various professional/scientific associations to participate in the retrospective survey. Data were collected from May, 2013 to Jan, 2014. Physicians were asked to report any AE case with ERCP. Data on patients’ and disease characteristics, endoscopic techniques and follow-up filled-out through an online questionnaire were analysed. Ethical Committee of Franche-Comté approved this study.

**Results:** Between 1986 and 2014, 12 centres performed ERCP for AE in Europe. Detailed data available for 23 patients (18 men and 5 women) in 8 centres were analysed. Sixty-one ERCP were performed (median: 2 {1–9} ERCP per patient). Patients were 55-years-old at AE diagnosis and 60-years-old at first ERCP. Indications for ERCP were: biliary pain, 14 (23%), cholangitis, 24 (39%), jaundice or chronic cholestasis, 22 (36%). Seventy-four plastic stents and 7 fully (5) or partially (2) covered self-expandable metallic stents (SEMS) were placed in the biliary tree. The average time between two stenting procedures was 19.5 weeks (1–98). Two patients needed surgical intervention or radiological drainage because of endoscopic treatment failure. There were 11 adverse events (18%): 1 perforation, 1 hepatic collection, 4 acute pancreatitis, and 5 cholangitis. Cholestasis disappeared in 42/44 therapeutic ERCP (95.4%). Biliary duct calibration in 8 patients after an average of 2 procedures; stent removal was possible after a median time of 45 weeks of treatment. Among them, recurrence of stenosis led to a new stent placement 4 years later in 1 patient and gallstones extraction in 2 patients.

**Conclusion:** Endoscopic retrograde drainage is efficient in AE with biliary obstruction. Less invasive than surgery and avoiding long-term external drainage, it is a valid alternative to radiological and surgical drainage. Several procedures are usually needed to ensure long-term efficacy and antibiotics are necessary during and after the ERCP.

##### O-29. Is adjuvant albendazole treatment really needed with PAIR in the management of liver hydatid cysts? A prospective randomized trial with short term follow-up

Akhan Okan^1^, Yildiz Adalet Elcin^1^, Akinci Devrim^1^, Yildiz Dogu^2^, Ciftci Turkmen^1^

^1^Hacettepe University, Ankara, Turkey

^2^Numune Teaching Hospital, Ankara, Turkey

akhano@tr.net

**Objective:** The aim of this study was to determine the safety and efficacy of adjuvant Albendazole medication in percutaneous liver hydatid cyst treatment with puncture, aspiration, injection and reaspiration (PAIR) method.

**Materials and Methods:** Between November 2007 and May 2011, total of 39 patients with newly diagnosed liver hydatid cyst (total of 77 cysts) were prospectively randomized and enrolled in 3 groups. In the first group, cysts (*n* = 14) were treated with PAIR without Albendazole. In the 2nd (*n* = 16) and 3rd group (*n* = 47), cysts were treated with PAIR with Albendazole 1 week before and 1 month post-procedure; with Albendazole 1 week before and 3 months post-procedure respectively.

**Results:** Technical and clinical success rates were 100% and 96.1% respectively. In 3 of 77 cysts (3.9%) findings of recurrence were detected on US imaging. All recurrent cysts were in group 1 and recurrence rates in this group were statistically different from cysts of 2nd and 3rd group (*p* = 0.005). Side effects of Albendazole were detected in 7 of 29 patients (24.1%) and no statistically significant difference was observed between 2nd (15.3%) and 3rd (38.4%) group (*p* = 0.378).

**Conclusion:** Use of Albendazole medication as an adjuvant to percutaneous treatment of liver hydatid cyst decreases the recurrence rate.Although there is no statistically significant difference between groups 2 and 3 in terms of efficacy and recurrence rate, patients in group 3 had a higher rate of side effect. Therefore, we conclude that Albendazole treatment 1 week before and 1 month after PAIR treatment is sufficient to reduce/prevent recurrences.

##### O-30. Long-term results of percutaneous treatment of CE 2/CE 3b (Gharbi type III) liver hydatid cysts: a retrospective comparison study of three percutaneous techniques

Akhan Okan^1^, Erbahceci Aysun^2^, Akinci Devrim^1^, Islim Filiz^2^, Ciftci Turkmen^1^, Akpinar Burcu^1^

^1^Hacettepe University, Ankara, Turkey

^2^Bakirkoy State Hospital, Ankara, Turkey

akhano@tr.net

**Objectives:** To determine the safety, effectiveness and recurrence rates of different percutaneous treatment techniques used for treating Gharbi type III (CE 2/CE 3b) liver hydatid cysts with long-term results and to introduce successful results of the new treatment technique we named MoCaT.

**Materials and Methods:** A total of 75 Gharbi Type III liver hydatid cysts (Type CE 2, CE 3b based on the WHO classification) in 73 patients who were treated percutaneously between March 1991 and August 2008 were evaluated retrospectively. The ages of patients were ranged between 6 and 79 (mean 38.6). Twenty-three of the cysts were treated with PAIR (percutaneous aspiration injection reaspiration), 26 with standard catheterization (catheterization technique with hypertonic saline and alcohol) and 26 with MoCaT (Modified catheterization) techniques. All of the procedures were carried out under general anaesthesia and guided by Ultrasonography and Fluoroscopy. Mean follow-up period after the interventions was 55.26 months (range 3–197 months).

**Results:** The technical success rate was 100%. No mortality occurred. Major and minor complication rates were 21.8% (n: 17) and 18% (n: 14), respectively. No recurrence was observed after treatment with MoCaT technique while recurrence was observed in the 43.5% of the patients who were treated with PAIR and 10.7% of the patients who were treated with standard catheterization techniques. All recurrences were also treated successfully by percutaneous techniques except one patient treated surgically after recurrence. The average catheterization duration in all the catheterized patients was 9.32 days. The hospital stay of all the patients was between 0 and 82 days with an average of 5.81 days.

**Conclusion:** Percutaneous treatment with MoCaT technique we developed is an effective and safe option in the treatment of Gharbi type III (CE 2/CE 3b) liver hydatid cysts. MoCaT is associated with lower recurrence rate and acceptable and higher complication rate when comparing the results of PAIR and Catheterization techniques.

#### Posters

##### P-46. Results of first line non-surgical strategy in the management of liver hydatid cyst with biliary fistula

Benazzouz Mustapha, Khannoussi Wafaa, Bakari Ghizlane, Afifi Rajaa, Essamri Wafaa, Benelbarhdadi Imane, Ajana Fatima Zohra, Essaid Abdellah

Medecine C Hepato-Gastroenterology Unit, Ibn Sina University Hospital, Rabat, Morocco

benaz21@hotmail.com

**Introduction**: Liver ecchinococcosis is an endemic disease in Morocco. Intrabiliary rupture is a frequent complication (5–25%) it can be occult or a frank rupture causing biliary obstruction. The main treatment strategy in such situation is surgery.in this study our objective is to evaluate the non-surgical treatment as a first line therapy.

Patients and methods: We included all patients managed with liver hydatid cyst (LHC) complicated with occult fistula (yellowish liquid on ultrasound guided percutaneous punction), or frank fistula (duct dilation ± hydatid material on imaging). In the first case, the treatment was based on percutaneous drainage alone, associated in second line with endoscopic biliary clearance in case of failure. In the second case, the first line treatment was endoscopic alone, associated in second line to percutaneous drainage in case of failure. For percutaneous drainage we used a large bore catheter 12–14 Fr .In case of failure of both strategies, surgery was the third line. In all cases Albendazole therapy was used for 3 months.

**Results:** In 18 years we managed 227 LHC, 47 (20.7%) had biliary rupture: 31 occult fistulas (65.9%) and 16 frank fistula (34.1%). In the first group, percutaneous drainage alone was curative in 24 cases (80.6%).Endoscopic treatment was associated with percutaneous drainage in one case with good result and in the other six cases surgery was performed. In the second group, endoscopic treatment alone succeeded in 9 cases (56%). Combined strategy was performed in 7 cases (44%) and failed in 3 cases leading to surgery. In final 38 patients were treated without surgery (80.8%) No complication was seen in all percutaneous and endoscopic procedures.

**Conclusion:** In our experience, the non-surgical treatment was successful in 80.8% of cases of LHC with biliary fistula. It was important to distinguish between two types of rupture to adopt the best treatment strategy. The first line is percutaneous drainage in case of occult fistula and endoscopic drainage in case of frank fistula. It was an efficient and safe strategy challenging the standard first line surgical therapy.

### 3.B. New drug targets

#### State-of-the-art

##### L-06. Albendazole and mebendazole: what else? Chemotherapy of echinococcosis: novel drugs on the horizon

Hemphill Andrew^1^, Stadelmann Britta^1^, Aeschbacher Denise^1^, Spiliotis Markus^1^, Gorgas Daniela^2^

^1^Institute of Parasitology, University of Bern, CH-3012 Bern, Switzerland

^2^Department of Clinical Veterinary Medicine, Clinical Radiology, University of Bern, CH-3012 Bern, Switzerland

andrew.hemphill@ipa.unibe.ch

The current chemotherapy of alveolar echinococcosis (AE) relies on albendazole and mebendazole. These are presently used for the treatment of non-surgical cases, and as a supplementary treatment prior and post-surgery. During AE, metacestodes proliferate asexually by exogenous and/or endogenous budding and infiltrate the neighbouring tissue. They form a complex mass consisting of peripherally proliferating metacestodes and centrally located necrotic tissue, all intermingled with connective tissue and immune cells, which is difficult to treat. While the current application of benzimidazoles has surely contributed to a significant improvement of living conditions and life span of affected patients, treatment failures and the occurrence of side effects have been reported, leading to discontinuation of treatment or to progressive disease. In any case, novel chemotherapeutical options should be developed to cure AE and CE.

One approach to speed up drug discovery for AE is to find new uses for already approved drugs, or compounds and compound classes that are already in development This piggi-back approach, called “drug repositioning”, has been widely used in the field of rare and neglected diseases such as AE, where the interests of the pharma companies are not compatible with the expected financial benefits in drug development. Thsi process also has significant commerical value for pharma companies, as it extends the range of applications for a compound and has only limited financial risks. In addition, for researchers as well as patients, the risk is also diminished, since the work is carried out on druggable targets, materials and data on e.g. long-term toxicity studies are available and can be presented to the relevant authorities, and this can lead to a reduced time frame between research and actual application.

Our laboratory has developed applied a range of *E. multilocularis* cell-based and metacestode-based drug screening assays employing compound libraries from different commercial and non-commerical sources (FDA, MMV-malariabox, several academic collaborators). We identified several mefloquin-, artemisinin- and pentamidine-derivatives (Küster et al., 2011, 2014a), as well as ruthenium-based organometallic compounds (Küster et al., 2012a), which show interesting capacities to kill the metacestodes *in vitro*. Recent investigations on several benzmidazoles showed that they all exhibited similar efficacies as albendazole *in vitro* and *in vivo*, and ultrastructural studies point towards a similar mode of action of these benzimidazoles, not necessarily related to the previously described disruption of microtubular cytoskeletal organisation (Küster et al., 2014 b). For *in vivo* studies, the mouse model has been further optimized and refined by establishing (i) a subcutanous model that allows closer monitoring of parasite growth (Küster et al., 2013a); (ii) voluntary oral application of drugs emulsified in honey as opposed to gavage (Küster et al., 2012b); and (iii) more recently we have been exploring ultrasound imaging for real time monitoring of effectsof anti-parasitic drugs. *In vivo* studies in the mouse model demonstrated the efficacy of oral application of the anti-malarial compound mefloquin, which will be followed up by studies on a range of mefloquine-derivatives that are predicted to have a lower capactiy to cross the blood-brain barrier. Promising *in vivo* effects were also obtained with a pentamidine derivative, DB1127, however, only when the drug was applied intraperitoneally (Küster et al., 2013b). In addition, investigations on potential targets of selected drugs in *Echinococcus* are being carried out in order to explore their potential mechanism of action in the metacestode stage.

**References**

Küster T, Stadelmann B, Hermann C, Scholl S, Keiser J, Hemphill A. In vitro and in vivo efficacies of mefloquine-based treatment against alveolar echinococcosis. Antimicrob Agents Chemother 2011. 55(2):713-21. doi:10.1128/AAC.01392-10.

Küster T, Lense N, Barna F, Hemphill A, Kindermann MK, Heinicke JW, Vock CA. A new promising application for highly cytotoxic metal compounds: η6-areneruthenium (II) phosphite complexes for the treatment of alveolar echinococcosis. J Med Chem 2012a. 55(9):4178–88. doi: 10.1021/jm300291a.

Küster T, Zumkehr B, Hermann C, Theurillat R, Thormann W, Gottstein B, Hemphill A. Voluntary ingestion of antiparasitic drugs emulsified in honey represents an alternative to gavage in mice. J Am Assoc Lab Anim Sci 2012b. 51(2):219–23.

Küster T, Hermann C, Hemphill A, Gottstein B, Spiliotis M. Subcutaneous infection model facilitates treatment assessment of secondary Alveolar echinococcosis in mice. PLoS Negl Trop Dis 2013a. 7(5):e2235. doi:10.1371/journal.pntd.0002235.

Küster T, Kriegel N, Boykin DW, Stephens CE, Hemphill A. In vitro and in vivo activities of dicationic diguanidino compounds against Echinococcus multilocularis metacestodes. Antimicrob Agents Chemother 2013b. 57(8):3829–35. doi: 10.1128/AAC.02569-12.

Küster T, Kriegel N, Stadelmann B, Wang X, Dong Y, Vennerstrom JL, Keiser J, Hemphill A. Amino ozonides exhibit in vitro activity against Echinococcus multilocularis metacestodes. Int J Antimicrob Agents 2014a. 43(1):40–6. doi: 10.1016/j.ijantimicag.2013.09.012.

Küster T, Stadelmann B, Aeschbacher D, Hemphill A. Activities of fenbendazole in comparison with albendazole against Echinococcus multilocularis metacestodes in vitro and in a murine infection model. Int J Antimicrob Agents 2014b. in press (http://dx.doi.org/10.1016/j.ijantimicag.2014.01.013)

Stadelmann B, Küster T, Scholl S, Barna F, Kropf C, Keiser J, Boykin DW,Stephens CE, Hemphill A. In vitro efficacy of dicationic compounds and mefloquine enantiomers against Echinococcus multilocularis metacestodes. Antimicrob Agents Chemother 2011. 55(10):4866–72. doi: 10.1128/AAC.00478-11.

#### Key-note

##### L-07. *Echinococcus multilocularis* genomics: an opportunity to disclose new therapeutic targets

Brehm Klaus

Institute of Hygiene and Microbiology, University of Würzburg, Würzburg, Germany

kbrehm@hygiene.uni-wuerzburg.de

In 2004, the author’s *Echinococcus* research group and the group of Matt Berriman (Wellcome Trust Sanger Institute) engaged in a project aiming at whole genome sequencing of *Echinococcus multilocularis* as a model cestode, which was later joined by research groups from Uruguay (C. Fernandez), Argentina (M. Rosenzvit), Mexico (J.-P. Laclette; UNAM), China (X. Cai), and the London Natural History Museum (P. Olson) to also include *E. granulosus*, *Taenia solium*, and *Hymenolepis microstoma* (model for adult worms) in genome sequencing. In parallel to genome sequencing, and necessary for gene annotation, extensive EST- and NextGen transcriptome sequencing of several *E. multilocularis* life-cycle stages was carried out. In spring 2013, this project led to a highly recognized publication in Nature (Tsai et al., 2013), and was followed shortly after by a second paper on the *E. granulosus* genome (Zheng et al., 2013), largely confirming the data obtained for *E. granulosus* by Tsai et al. (2013). When compared to genomes of free-living flatworms (e.g. planarians) and schistosomes, extensive gene loss (e.g. cholesterol synthesis genes) and gene gain (e.g. antigens for lipid uptake) associated with the evolution of parasitism was observed. Most notably, cestodes, like trematodes, have a highly modified stem cell system and obviously lack stem cell specific markers such as *vasa* and *piwi*, which are associated with transposon silencing and genome integrity maintenance of all other bilateria (Tsai et al., 2013; Koziol et al., 2014).

In addition to highly interesting data concerning parasite biology and host-parasite interaction, these genomes also revealed novel clues and targets concerning anti-parasitic chemotherapy. Current benzimidazole chemotherapy (targeting beta-tubulin) is obviously ineffective in eliminating *Echinococcus* germinative cells, which is the only cell type in these parasites capable of dividing and yielding all differentiated cell types (Koziol et al., 2014). The likeliest reason for this is that germinative cells express a potentially benzimidazole-resistant beta-tubulin (Olson et al., 2012). A highly interesting group of druggable targets that are expressed in germinative cells are protein kinases (250 in the entire genome) which regulate cell proliferation and differentiation. Several anti-kinase small molecule compounds that are currently in use in cancer therapy have already been shown to also affect *Echinococcus* kinases, larvae and stem cells *in vitro* at concentrations comparable to those found in serum of cancer patients (Gelmedin et al., 2008; Hemer et al., 2012, 2014). These molecules are currently in use as lead compounds to identify drugs that act with higher specificity on parasite kinases than on the orthologous human kinases.

Additional targets of potential use for anti-echinococcosis drug design are involved in purine metabolism, oxygen detoxification, or cell-cycle regulation (Tsai et al., 2013) and respective small-molecule compounds are currently tested in cultivation systems for the parasite metacestode (Spiliotis and Brehm, 2009) and germinative cells (Spiliotis et al., 2008).

Finally, the recent description of a nerve net in the metacestode (Koziol et al., 2012) and of a family of highly druggable G-protein-coupled receptors that are nerve net-specifically expressed should be of high interest for the development of novel anti-echinococcosis chemotherapeutics.

**References**

Gelmedin V, Caballero-Gamiz R, Brehm K. Characterization and inhibition of a p38-like mitogen activated protein kinase (MAPK) from *Echinococcus multilocularis*: antiparasitic activities of p38 MAPK inhibitors. Biochem Pharmacol 2008;76: 1068–1081.

Hemer S, Brehm K. In vitro efficacy of the anti-cancer drug Imatinib on *Echinococcus multilocularis* larvae. International J Antimicrob Agents 2012; 40: 458–462.

Hemer S, Konrad C, Spiliotis Koziol U, M., Schaack D, Förster S, Gelmedin V, Stadelmann B, Dandekar T, Hemphill A, Brehm K. Host insulin stimulates *Echinococcus multilocularis* insulin signalling pathways and larval development. BMC Biol 2014;12: 5.

Koziol U, Krohne G, Brehm K. Anatomy and development of the larval nervous system in *Echinococcus multilocularis*. Frontiers Zool 2013;10: 24.

Koziol U, Rauschendorfer T, Zanon-Rodriguez L, Krohne G,

Brehm K. The unique stem cell system of the immortal larva of the human parasite *Echinococcus multilocularis*. EvoDevo 2014;5: 10.

Olson PD, Zarowiecki M, Kiss F, Brehm K. Cestode genomics – progress and prospects for advancing basic and applied aspects of flatworm biology. Parasite Immunol 2012;34: 130–150.

Spiliotis M, Lechner S, Tappe D, Scheller C, Krohne G, Brehm K. Transient transfection of *Echinococcus multilocularis* primary cells and complete in vitro regeneration of metacestode vesicles. Int J Parasitol 2008;38: 1025–1039.

Spiliotis M, Brehm, K. Axenic in vitro cultivation of *Echinococcus multilocularis* metacestode vesicles and the generation of primary cell cultures. Meth Molec Biol 2009;470: 245–262.

Tsai IJ, Zarowiecki M, Holroyd N, Garciarrubio A, Sanchez-Flores A, Brooks KL, Tracey A, Bobes RJ, Fragoso G, Sciutto E, Aslett M, Beasley H, Bennett HM, Cai J, Camicia F, Clark R, Cucher M, De Silva N, Day TA, Deplazes P, Estrada K, Fernández C, Holland PW, Hou J, Hu S, Huckvale T, Hung SS, Kamenetzky L, Keane JA, Kiss F, Koziol U, Lambert O, Liu K, Luo X, Luo Y, Macchiaroli N, Nichol S, Paps J, Parkinson J, Pouchkina-Stantcheva N, Riddiford N, Rosenzvit M, Salinas G, Wasmuth JD, Zamanian M, Zheng Y; Taenia solium Genome Consortium, Cai X, Soberón X, Olson PD, Laclette JP, Brehm K, Berriman M. The genomes of four tapeworm species reveal adaptations to parasitism. Nature 2013;496: 57–63.

Zheng H, Zhang W, Zhang L, Zhang Z, Li J, Lu G, Zhu Y, Wang Y, Huang Y, Liu J, Kang H, Chen J, Wang L, Chen A, Yu S, Gao Z, Jin L, Gu W, Wang Z, Zhao L, Shi B, Wen H, Lin R, Jones MK, Brejova B, Vinar T, Zhao G, McManus DP, Chen Z, Zhou Y, Wang S. The genome of the hydatid tapeworm *Echinococcus granulosus*. Nature Genetics 2013;45: 1168–1175.

#### Oral communications

##### O-31. Genome-wide sequencing of small RNAs of *Echinococcus granulosus* shows micro-RNAs may be involved in life cycle stage development and differentiation

Bai Yun^1^, Zhang Zhuangzhi^2^, McManus Donald P.^3^, Zhang Wenbao^2,4^, Wang Shengyue^1^

^1^Shanghai-MOST Key Laboratory of Health and Disease Genomics & Chinese National Human Genome Center at Shanghai, Shanghai, China

^2^Veterinary Research Institute, Xinjiang Academy of Animal Sciences, Urumqi, Xinjiang, China

^3^Molecular Parasitology Laboratory, QIMR Berghofer Institute of Medical Research, Brisbane, Queensland, Australia

^4^State Key Laboratory Incubation Base of Xinjiang Major Diseases Research, Clinical Medical Research Institute, First Affiliated Hospital of Xinjiang Medical University, Urumqi, Xinjiang, China

wangsy@chgc.sh.cn; wenbaozhang2013@163.com

**Background:** MicroRNAs (miRNAs) are important post-transcriptional regulators which control growth and development in eukaryotic species. The cestode *Echinococcus granulosus* has a complex life-cycle involving different development stages but the mechanisms underpinning this development, including the involvement of miRNAs, remain to be elucidated.

**Methods:** Using Illumina deep-sequencing technology, we sequenced at the genome-wide level three small RNA populations from the adult, protoscolex and cyst membrane of *E. granulosus*.

**Results:** A total of 176 miRNA candidates were *in silico* predicted. Through comparison, we found that 48 mature miRNAs and 17 miRNA*s were expressed in different patterns in the three life stages. Most of the differentially expressed miRNAs exhibited obvious up-regulated expressions in the adult stage and down-regulation in the hydatid cyst. In addition, two new miRNA clusters were identified in *E. granulosus* and *E. multilocularis*. They contained similar sequence at the “seed region” and mainly expressed at adult stage. A total of 3,622 genes were predicted to be targets of 126 mature miRNAs and 50 miRNA*s. Further analysis of the differentially expressed miRNAs and their potential targets indicates that they may be involved in bi-directional development, nutrient metabolism and nervous system development in *E. granulosus*.

**Discussion/Conclusion:** Our results, for the first time, provided a comprehensive description of the different expression patterns of miRNAs in the three distinct life cycle stages of *E. granulosus*. Understanding the regulatory processes involving miRNAs in *E. granulosus* may help in the exploration of the mechanisms of interaction between this parasitic worm and its definitive and intermediate hosts and provide new clues to developing new intervention strategies for control of cystic echinococcosis.

##### O-32. The novel CD4^+^ CD25^+^ regulatory T cell effector molecule Fibrinogen-Like Protein 2 (FGL2) contributes to the outcome of murine alveolar echinococcosis

Wang Junhua^1,2,3^, Huber Cristina^1^, Müller Norbert^1^, Vuitton Dominique A.^4^, Blagosklonov Oleg^3,4^, Lu Xiaomei^2^, Lin Renyong^2^, Wen Hao^2^, Gottstein Bruno^1^

^1^Institute of Parasitology, University of Bern, Bern, Switzerland

^2^State Key Lab Incubation Base of Xinjiang Major Diseases Research (2010DS890294) & Xinjiang Key Laboratory of Echinococcosis, First Affiliated Hospital of Xinjiang Medical University, Urumqi, Xinjiang, China

^3^Department of Nuclear Medicine, University Hospital, Besançon, France

^4^WHO-Collaborating Centre for the Prevention and Treatment of Human Echinococcosis, University of Franche-Comté and University Hospital, Besançon, France

xjwangjh@126.com; bruno.gottstein@vetsuisse.unibe.ch; dominique.vuitton@univ-fcomte.fr

**Background:** The immunology of murine alveolar echinococcosis (AE) is characterized by the development of immune tolerance against the *Echinococcus multilocularis (E.multilocularis)* metacestode allowing the parasitic tumor-like tissue to continuously proliferate and metastasize. Parasite proliferation is dependent on the nature of the periparasitic inflammatory and other immune-mediated processes. In a previous study, fibrinogen-like protein 2 (FGL2) was found to be up-regulated in AE-infected *vs* non-infected control animals. So far, nothing is known on the contribution of this novel CD4^+^ CD25^+^ regulatory T cell (Treg) effector molecule to the control of a helminth infection.

**Methods:** FGL2^−/−^ mice were experimentally infected with *E. multilocularis,* and age-and-gender-matching wild type (WT) animals were used as controls. Mice were sacrificed at 1 and 4 month(s) post-infection (p.i.). As a key parameter for infection outcome, parasite load was measured by wet weight determination of the metacestode tumor-like tissue, and serum FGL2 levels were measured by sandwich enzyme-linked immunosorbent assay. Spleen cells were firstly analyzed ex-vivo and secondly after being cultured with ConA stimulation for 48 h or with *E. multilocularis* Vesicle Fluid (VF) antigenic stimulation for 96 h. Spleen cells from non-infected WT mice were cultured with rFGL2/anti-FGL2 or rIL-17A/anti-IL-17A for further functional studies. For the Treg immune suppression assay, high purity of CD4^+^ CD25^+^ Tregs (N > 99%) was achieved by MACS then FACS. These purified cells were incubated, together with CD4^+^ effector T cells and irradiated spleen cells as APCs, with ConA for 48 h. Flow cytometry and real time RT-PCR were used to determine T cell subpopulations including Treg numbers and phenotypes, Th17-, Th1-, Th2-type immune responses, and maturation of dendritic cells and B cells.

**Results:** FGL2-deficient mice infected with *E. multilocularis* exhibited a significantly decreased parasite load, associated with increased T cell proliferation in response to ConA, impaired Treg numbers and function, relative Th1 polarization, and increased numbers of antibody-producing B cells, as compared to infected WT mice. Both relative number and maturation status of dendritic cells were higher in fgl2^−/−^ mice, and CD80 and CD86 were more expressed in DCs following ConA and VF stimulation. Additional experiments confirmed that IL-17A contributes to FGL2 secretion in this model.

**Conclusions:** Our data demonstrate that FGL2, together with IL-17 and by promoting Treg cell activity, appears as a key-player in the orchestration of the outcome of *E. multilocularis* infection; this study gives evidence for a role of IL-17 in FGL2 regulation, and suggests that targeting FGL2 could be used for the development of novel treatment approaches in infectious diseases.

#### Posters

##### P-47. Experience of long-term follow-up for liposomal albendazole in treating complex hepatic alveolar echinococcosis

Li Haitao^1,2^, Song Tao^2,3^, Qin Yongde^4^, Shao Yingmei^1,2^, Tuergan Aili^1,2^, Ahan Ayifuhan^1,2^, Ran Bo^1^, Wen Hao^1,2^

^1^Hepatobiliary and Hydatid Department, Digestive and Vascular Surgery Centre, First Affiliated Hospital of Xinjiang Medical University, Urumqi 830011, China

^2^State Key Lab Incubation Base of Xinjiang Major Diseases Research (2010DS890294) and Xinjiang Key Laboratory of Echinococcosis, First Affiliated Hospital of Xinjiang Medical University, Urumqi 830011, China

^3^Department of Ultrasonography, First Affiliated Hospital of Xinjiang Medical University, Urumqi 830011, China

^4^Department of nuclear medicine, First Affiliated Hospital of Xinjiang Medical University, Urumqi 830011, China

dr.wenhao@163.com

**Background:** In clinic, hepatic alveolar echinococcosis (HAE) which cannot be excised radically or with extra-hepatic organs being involved is called “complex hepatic alveolar echinococcosis” (C-HAE), Chemotherapy usually must be taken by such patients with C-HAE for a lifelong time.

**Methods:** We summed clinical effect of liposomal albendazole (L-ABZ) in treating these patients with C-HAE by longtime follow-up. After retrospectively recording the clinical cases in our hospital with a follow-up over five years, 52 cases were included in our study. The follow-time was from 5 to 23 years with an average of 9.8 years.

**Results:** Among the 52 cases, the lesion of 28 cases was located in the liver alone; the lung was involved in 17 cases, the kidney in 2 cases, and the brain in 9 cases. General five-year survival rate was 82.6% in 52 cases (43/52), the five-year survival rate of patients treated with chemotherapy alone was 82.7% (24/29), the five-year survival rate of patients treated with palliative surgery plus chemotherapy was 82.6% (19/23), there was no statistical difference between two groups (precise probability, *P* = 1.000). All patients were followed up by computed tomography (CT) or nuclear Magnetic Resonance examination (MRI) to evaluating the clinical effect of chemotherapy, different organs showed different change in images: The hepatic lesion of all cases showed to be solid (9 cases), to form liquefied necrotic cavity (26 cases) and keep silent (6 cases). The lung lesion of 17 cases showed formation of the hollow (3 case), or kept silent (12 cases). The cerebral lesion of 9 cases showed to become solid and the cerebral edema surrounding the lesion disappeared in 6 cases. Furthermore, the means for judging medical effect in treating HAE not only included CT scan and MRI check, some new methods such as positron emission tomography/computed tomography (PET/CT) and contrast-enhanced ultrasound also was important way to assess clinical effect by observing biological activity of HAE lesion.

**Conclusion:** In our study, long-time treatment of L-ABZ is effective, it not only could relieve patient’s symptoms, thus improving patients’ quality of life, but also could control progress of lesion, thus prolonging patient’s living time. Moreover, the result showed there was no difference of therapeutic efficacy between chemotherapy alone and palliative surgery plus chemotherapy.

##### P-48. Characterization of a P38-like Mitogen-Activated Protein Kinase (MAPK) from *Echinococcus granulosus*: a key molecular mediator between the host TGF-β and *E. granulosus*

Lu Xiaomei^1^, Lin Renyong^1^

^1^State Key Lab Cultivation Base for Xinjiang Major Diseases Research and Xinjiang Key Laboratory of Echinococcosis, First Affiliated Hospital of Xinjiang Medical University, Urumqi, China

renyong_lin@sina.com

**Background:** The p38 mitogen-activated protein (MAP) kinases are a family of serine/threonine protein kinases that play important roles in cellular responses to a wide variety of external stress and play essential role in the survival of parasites.

**Methods:** A gene encoding p38-like mitogen-activated protein kinase (Egp38) of *Echinococcus granulosus* was cloned by RT-PCR and sequenced to analyze the domains, phylogeny and tertiary structure. The expression pattern of EgP38 was tested by Western blot and immuno-histochemisty. To test the relationship between the host cytokine TGF-β and the activity of Egp38, the protoscoleces of *E. granulosus* were treated by human TGF-β in vitro.

**Results:** DNA sequence analysis showed that it is a new p38-homologue gene comprised 1107 bp, coding 368 amino acids, named Egp38, which has 43.04 to 98.64% homology to p38 from *E. multilocularis, D. melanogaster* and *Homo sapiens*, respectively. Bioinformatics analysis predicted that EgP38 contains a highly conserved T-X-Y motif and the activation loop segment of p38-like kinase. Structure homology modelling recovered the conserved structure among the EgP38 and human P38α, implying a common predicted binding mechanism in the ligand domain and the same downstream signal transduction processing in the tyrosine kinase domain as in P38α. Western blot and immuno-histochemisty analysis revealed that Egp38 and its phosphorylated form was expressed in the larval stages germinal layer and protoscolex of *E. granulosus* during an infection of intermediate host. When 1 μM TGF-β was added to in vitro cultivated protoscoleces, the Egp38 activity could effectively be enhanced with time.

**Discussion:** The present results indicate the existence in *E. granulosus* of Egp38, which mediates the interplay between the host and *E. granulosus,* and could be a new target for drug and vaccine development against Cystic Echinococcosis.

##### P-49. Identification and preliminary characterization of a novel molecule, potential target for treatment in secondary hydatidosis

Naidich Ariel, Gutierrez Ariana Marcela

Departamento Parasitología, INEI-ANLIS “Dr. Carlos G. Malbran”, Buenos Aires, Argentina

anaidich@anlis.gov.ar

**Background:** Human hydatid disease produces cysts in several anatomic locations, formed by the larval stage of *Echinococcus granulosus*. If a primary cyst breaks, several new cysts could be produced (secondary hydatid disease). Cyst formation process is not known yet. Some papers describe proteases acting in early events during invasion and penetration in host-parasite interphase. It has been reported that some parasite proteases could produce extracellular matrix disruption. The presence of a protease in this parasite and a possible role in cyst formation may be a potential target for treatment using specific inhibitors. The objective of the present work was to search and characterize proteases in protoescoleces of *E. granulosus*.

**Methods:** A search at EST database for *E. granulosus* was performed using conserved eucariotic protease active site sequences. RNA was extracted from protoscoleces obtained from pig cysts and a RT-PCR reaction with oligo dT was performed to obtain cDNA, which was used as template for PCR reactions with two set of primers, the first designed to amplify conserved flanking sequences of the active site of eucariotic proteases and the second homolog to a set designed to amplify two cystein peptidases of *E. mulitlocularis*. All PCR fragments obtained were cloned and sequenced. Obtained sequences were used as query in BLAST, BLASTx, BLAST CD search tools. Phylogenetic tree (MEGA 3.1, UPGMA) was constructed with similar sequences of related parasites and some *E. granulosus* hosts. Nucleotidic sequence was translated and spatially modelled using SWISS PROT and VMD. The 3D model was aligned based on secondary structures.

**Results:** The ESTs available in databases yielded no sequences with homology to eucariotic peptidases. The first set of primers amplified a fragment of about 500 bp and the second set yielded a 950 bp one. Both sequences were homolog to cystein proteases of C1A family. The two sequences were almost equal among each other in the coverage area. The shortest sequence translated had identity with a cystein peptidase of *E. multilocularis* (93%), and other cysteine peptidases (*Teania solium*, *T. asiatica* and *T. saginata*: 55%; *Fasciola hepatica*: 46%*).* Translated aminoacidic sequences had a conserved domain with corresponding residues of catalytic site and subsite. Phylogenic tree grouped putative peptidase in the same branch as a homolog *E. multilocularis* protein, close in evolution to sequences of *T. saginata, T. asiatica and T. solium*. The 3D model of putative peptidase confirmed spatial similarities to C1A cystein peptidase.

**Discussion/Conclusion:** We found a putative cystein peptidase in *E. granulosus*. Further studies of docking using 3D structure would allow knowledge about inhibitor interactions. We are performing biochemical and catalytical studies and localizing protein expression in parasite stage/location. To date results are promising in the way to identify a new potential target for treatment of secondary hydatidosis.

##### P-50. Axenic culture of *Echinococcus multilocularis* primary cells: a novel tool to carry out high-throughput screening assays on bioactive molecules

Spiliotis Markus, Stadelmann Britta, Hirschi Patrick, Gottstein Bruno, Hemphill Andrew

Institute of Parasitology, University of Bern, Switzerland

markus.spiliotis@vetsuisse.unibe.ch

**Background and aims:** Effects of bioactive molecules on cestode parasites have always been notoriously difficult to screen by high-throughput assays, since large amounts of host-free parasite material are needed and cestode in vitro culture under laboratory conditions is a laborious and cost-intensive undertaking. One of the best described cestodes with human impact is *E. multilocularis*. Metacestodes can be relatively easily cultured in vitro, and it is possible to produce large amounts of clean in vitro generated parasite material, such as metacestode vesicles or primary cells isolated from these metacestodes. So far, efficacy studies on bioactive molecules have been employing axenic metacestode cultures using a medium-throughput format, with phosphoglucose isomerase (PGI) as reporter for metacestode damage. Assays on primary cells could not be performed until now because no axenic primary cell culture system, devoid of feeder cells, was available.

**Results:** Here we describe the establishment and two distinct applications of a 96- or 384-well format axenic *E. multilocularis* primary cell culture system.

First, we applied and comparatively assessed the cell-based system on a small panel of drugs that have been reported earlier to be effective, or non-effective, against *E. multilocularis* metacestodes in vitro. All compounds tested (albendazole, amphotericin B, nitazoxanide, praziquantel, mefloquin, DB 1127, artesunate, artemether and OZ401) exhibited almost similar activities in both culture systems, and could discriminate clearly between effective and non-effective compounds. Based on the parasite material needed, approximately 20 times the numbers of compounds can be analyzed in the cell assay compared to the conventional metacestode assay. Thus, cell-based screening could be used as an initial, relatively rapid and easy-to-handle screening tool to identify active compounds, which are further validated in a second step in metacestodes. In vivo experiments in laboratory hosts are finally carried out to verify drug efficacy in a natural host-parasite system.

Second, we applied the axenic cell-based culture system to study the effects of growth factors such as insulin, EGF, FGF or heparin on parasite growth and development. Here, we observed clear differences between the different growth factors, and especially by adding FGF/heparin a highly accelerated differentiation from cell-aggregates to newly formed metacestodes within 7 days of culture was detected.

**Conclusion:** The possibility to induce the entire development of metacestodes, starting from primary cells to fully differentiated parasite larvae under axenic conditions allows now to dissect the events leading to growth factor-induced development or the identification and characterization of drug-induced inhibition without the interference of feeder cell derived side effects.

##### P-51. Cloning and analysis of ribosomal protein S9 gene from *Echinococcus granulosus* and effect of anti-hydatid drug intervention on the gene

Wang Jianhua^1^, Zhao Jun^1^, Xiao Yunfeng^1^, Lü Guodong, Gao Huijing^1^

^1^Department of Pharmacy, First Affiliated Hospital of Xinjiang Medical University, Urumqi 830054 China

^2^Clinical Medical Research Institute, First Affiliated Hospital of Xinjiang Medical University, Urumqi 830054 China

jhw716@163.com

**Background:** Ribosomal protein S9 (RPS9), widely distributed in yeast, bacteria, parasites, and mammals, is highly conserved and may be involved in DNA repair, translation regulation, developmental regulation and cancerisation, and is a potent drug target for tumors and some parasitoses. In schistosome research it was found that RPS9 provided new ways for development of new diagnostic reagent, another research showed that cell response to inhibit cancer cell proliferation could be caused after silencing the ribosomal protein S9, then activated P53 pathway, in the rapid growth of tumor cells. RPS9 may thus be effective to guide the different process or can induce senescence and apoptosis, suggesting that RPS9 can provide new ideas for drug design.

**Methods:** *Echinococcus granulosus* RPS9 (EgRPS9) gene was cloned from the cDNA of *E. granulosus* protoscolex and its sequence was analyzed by biology informatics. The gene expression pattern of EgRPS9 gene at different developmental stages of *E. granulosus* and drug intervention group were detected by real-time qPCR assay.

**Results:** According to the biology informatics analysis, EgRPS9 gene had a 554 bp open reading frame, which encoded 188 amino acids. Molecular weight of the encoded protein was 22.0 ku, with an isoelectric point of 10.32. SMART analysis showed that the region from 107 to 176 amino acids is S4 domain. Real-time qPCR results showed that there was no significant difference of the expression of EgRPS9 at different developmental stages of E. granulosus (*P* > 0.05). There was no statistically significant difference between groups of DMSO, dihydroartemisinin, praziquantel, nitazoxanid and control group (*P* > 0.05). However, it was significantly raised post-intervention in groups of albendazole, artemisinin, artesunate and artemether compared with control group (*P* < 0.05). In hydrogen peroxide-treated groups, the gene content were also dramatically increased (*P* < 0.001), and there was a dose-effect relationship within a certain range.

**Conclusion:** EgRPS9 gene was cloned for the first time. The gene was expressed at different developmental stages. In drug-intervention groups, EgRPS9 gene was significantly raised. Thus, we concluded that the mechanism of anti-hydatid may be related with oxidative damages. All results indicated that EgRPS9 gene may be a potential target for *E. granulosus* treatment. The research could establish a fundation for the novel drug of hydatid disease.

### 3.C. Round Table: How to improve patient care in echinococcosis?

#### 

##### L-08. Integrating echinococcosis into clinical practice: a centre-based approach

Junghanss Thomas

Section Clinical Tropical Medicine, University Hospital Heidelberg, Heidelberg, Germany

thomas.junghanss@urz.uni-heidelberg.de

Care for patients with cystic and alveolar echinococcosis has, as in most NTDs/NIDs, to overcome several bottlenecks: lacking recognition of the scale of the problem, lacking awareness of clinicians, lacking standards with diagnostic and treatment guidelines based on very limited evidence, need for interdisciplinary coordination in advanced stages of the disease. Paradoxically, in regions where the echinococcoses are most prevalent, resources are scarce, whereas in resource-rich countries patients are few, though increasing in numbers at the current scale of mobility.

To optimize care in low prevalence settings a centre-based approach secures best clinical practice for the individual patients’ benefit and generation of new knowledge by exploiting the availability of high tech resources such as various imaging modalities, immunological and molecular tools and interventional techniques to investigate new venues of diagnosis and treatment. Cross talking between clinicians from NTD/NID endemic countries and NTD/NID centres in high tech environments will facilitate translation of results into interventions adapted to settings where the diseases most prevail.

The setting and the impact of the Echinococcosis Centre at Heidelberg University Hospital is presented where infectious disease/tropical medicine physicians, radiologists, abdominal and thoracic surgeons, gastroenterologists and parasitologists work closely together to stage patients and to tailor currently available treatment options to the needs of the individual patient and to generate new knowledge by investigating carefully observed prospective patient cohorts.

##### O-33. Interdisciplinary trends in therapy of advanced stage alveolar echinococcosis

Hillenbrand Andreas^1^, Barth Thomas FE^2^, Henne-Bruns Doris^1^, Gruener Beate^3^

^1^Department of General and Visceral Surgery, University Hospital and Medical Center, Ulm, Germany

^2^Institute of Pathology, Ulm University, Ulm, Germany

^3^Division of Infectious Diseases, University Hospital and Medical Center, Ulm, Germany

andreas.hillenbrand@uniklinik-ulm.de

**Background:** Therapy of alveolar echinococcosis (AE) caused by E. mulitlocularis is challenging. We report our therapy stratification in patients referred to our hospital in 2012 and 2013 with newly diagnosed AE. Our therapy concept was evaluated by multi-disciplinary consensus and consists of medical therapy, interventional therapy, palliative and curative operations.

**Methods:** In 2012 and 2013 64 patients were referred to our hospital with newly diagnosed AE. The male : female ratio was 26:37 female with a median age at initial diagnosis of 54 years (range 16–71) resp. 56 years (range 12–79). At diagnosis six patients were younger than 20 years and six were between 20 and 30 years old. 27 patients presented with PII lesions, 12 patients with P III and 25 patients with P IV, respectively. 5 patients presented with disseminated AE disease (M-stage). 32 patients presented with resectable liver lesions suitable for surgery in a curative intent (i.e. in a preoperative assessment, operation could be performed with a safety margin).

**Results:** 57 patients received medicamentous therapy, seven did not. Reasons for no medication in these seven patients were incompatibility of the medication, missing activity in PET-CT, surgical resection, and differential diagnosis was more likely than AE. Curative surgery with safety margin was performed in 16 patients. In 7 patients AE was completely removed with safety margin <1 cm, so curative character of surgery was not for sure. Indications for surgery were haemorrhage, cava compression, infection, early age of diagnosis, incompatibility of the medication in young patients, or pain. According to the WHO guidelines, postoperative medication was continued for two years. Interventional treatment was performed in three patients (endoscopic stent placement, drainage system).

**Conclusion:** At diagnosis of AE all eligible patients should be treated with benzimidazole compounds. Patients regarded as eligible for surgery should have complete removal of the parasitic lesions. Surgery without safety margin may also be curative in certain cases, especially with adequate postoperative treatment.

##### L-09. What biological follow-up for what echinococcosis patients?

Gottstein Bruno

Institute of Parasitology, Vetsuisse Faculty and Faculty of Medicine, University of Bern, Switzerland

bruno.gottstein@ipa.unibe.ch

*CE, cystic echinococcosis:* Any kind of treatment for any case of CE implies a long-term follow-up of the patient, which is needed to detect relapses. On a first hand, standardized US-based monitoring is primarily used for the follow-up of CE patients, for some specific cases MRI or CT is alternatively applied (Stojkovic et al., 2014). The most important feature of this classification is the division of CE cysts into “active”, “transitional” and “inactive”. In addition, metabolic viability assessment of CE using high-field 1H-MRS of cyst content has been proposed to provide further information on cyst viability (Hosch et al., 2008). In parallel, follow-up laboratory analyses to monitor drug tolerance include liver enzymes (aminotransferases) and leucocyte count. The search for immunological parameters useful in the staging and prognosis of CE in treated patients has yielded scarce practical tools so far. Siracusano et al. (2012) reported on HSP20 as a potential marker of active CE. Their immunoblot analysis revealed anti-HSP20 antibodies in a statistically significant higher percentage of sera from patients with active disease than in sera from patients with inactive disease. Anti-HSP20 antibody levels significantly decreased over the course of chemotherapy in sera from patients with cured disease, when compared to sera from patients with progressive disease. Hernandez-Gonzalez et al. (2008) showed that the B2t-IgG-ELISA is of a better prognostic value for the follow-up of surgically treated CE patients than other conventional *Echinococcus*-ELISAs. Similar findings were reported by Ben Nouir et al. (2009), who documented a relatively rapid decrease of anti-recP29 antibody levels in young CE that had been successfully treated by surgery, while relapsing juvenile CE patients continued or newly developed an anti-recP29 level following treatment. Post-treatment positive specific IgG antibody seroconversion against recAgB, in initially seronegative, CE1 patients was considered a good indication for positive therapeutic efficacy of albendazole, as reported by Tiaoying et al. (2011). Prospectively, promising new tools helpful for post-treatment monitoring of CE patients by laboratory methods may rather be developed in the domain of circulating antigen detection, the disappearance/absence of which should more rapidly reflect parasite inactivation than conventional antibody detection (Zhang et al., 2012).

*AE, alveolar echinococcosis:* Diagnosis, staging and activity assessment of AE is primarily based on imaging, with US, MRI, CT and PET-CT or PET-MRI being primarily used in dependence of location of the parasite lesion(s). In unclear cases when imaging and serology do not lead to a diagnosis, fine-needle puncture and aspiration, analysed by histology, immunohistochemistry and most easily by PCR, is a valuable tool for the differential diagnosis, in particular of malignancies (Diebold-Berger et al., 1997). Testing of parasite viability can be performed with RT-PCR of biopsies and fine-needle aspirates upon various constitutively expressed gene targets (e.g. 14-3-3), within the limits of the sensitivity of this method (Ito and Craig, 2003, Matsumoto et al., 2006; Yamasaki et al., 2007).

Better than for CE, some specific serologic tests are valuable to assess the efficacy of treatment in combination with imaging during follow-up of patients (Siles-Lucas and Gottstein 2001; Ito and Craig 2003). Follow-up after surgery and chemotherapy is probably best life-long. Recurrences have been observed almost 20 years after surgery. After successful surgery and/or chemotherapy leading to inactivation of the parasite, anti-Em18 (and to a certain extent anti-Em2^+^) antibodies decline is rapid, and seroconversion to undetectable levels correlates well with curative resection (Ammann et al 2004; Tappe et al 2009). As for CE, there is evidence that for post-treatment monitoring of AE patients the detection of circulating antigens may more directly reflect parasite metabolism and thus viablity, or, in case of absence, parasite inactivation.

Prospectively, there is a remarkably strong demand by clinicians for improved imaging tools to assess in vivo the viability or non-viability status of treated hepatic and extra-hepatic AE-lesions. [18F]- fluorodeoxyglucose (FDG) is a validated tracer of AE lesions; however, it does not directly reflect parasite viability but rather peri-parasitic host inflammatory processes. The ideal tracer should be able to assess the course of AE upon direct uptake by the metacestode through its metabolic activity. Respective candidates may be searched among other experimental PET tracers such as [18F]-fluorotyrosine (FET), [18F]-fluorothymidine (FLT), [18F]-fluorometylcholine (FMC), and sodium [18F]-fluorine (NaF) (Porot et al., 2013). Therapeutically, one might envisage to use a similar tracer or ligand technique upon use of other isotopes such as Indium 111, Lutetium 177, Yttrium 90 or Zirconium 89. Such methodologies will require appropriate animal models suitable to be tested by micro-PET CT or MRI analyses, mice and rats appear as the most suitable animal species so far for susceptibility studies, while minipigs may be used to address host resistance mechanisms.

**References**

Ammann RW, Renner EC, Gottstein B, Grimm F, Eckert J, Renner EL, Swiss Echinococcosis Study Group: Immunosurveillance of alveolar echinococcosis by specific humoral and cellular immune tests: prospective long-term analysis of the Swiss chemotherapy trial (1976-2001). J Hepatol 2004;41: 551–559

Ben Nouir N, Gianinazzi C, Gorcii M, Müller N, Nouri A, Babba H, Gottstein B. Assessment of Echinococcus granulosus Somatic Protoscolex Antigens for Serological Follow-Up of Young Patients Surgically Treated for Cystic Echinococcosis. Transactions of the R Soc Trop Med Hyg 2009;103: 355–364.

Diebold Berger S, Khan H, Gottstein B, Puget E, Frossard JL, Remadi S. Cytologic diagnosis of isolated pancreatic alveolar hydatid disease with immunologic and PCR analyses – A case report. Acta Cytol 1997;41: 1381–1386.

Hernández-González A, Muro A, Barrera I, Ramos G, Orduña A, Siles-Lucas M. Usefulness of Four Different *Echinococcus granulosus* Recombinant Antigens for Serodiagnosis of Unilocular Hydatid Disease (UHD) and Postsurgical Follow-Up of Patients Treated for UHD. Clin Vaccine Immunol 2008;15: 147–153.

Hosch W, Stojkovic M, Jänisch T, Kauffmann GW, Junghanss T. The role of calcification for staging cystic echinococcosis (CE). Eur Radiol 2007;17:2538–2545.

Ito A, Craig PS. Immunodiagnostic and molecular approaches for the detection of taeniid cestode infections. Trends Parasitol 2003;19: 377–381.

Li A, Ito A, Pengcuo R, Sako Y, Chen X, Qi D, Xiao N, Craig PS. Post-Treatment Follow-Up Study of Abdominal Cystic Echinococcosis in Tibetan Communities of Northwest Sichuan Province, China. PLoS Negl Trop Dis 2011; 5(10): e1364.

Li T, Xingwang Chen X, Ren Zhen R, Jiamin Qiu J, Dongchuan Qiu D, Ning Xiao N, Akira Ito A, Hu Wang Hu, Patrick Giraudoux, Yasuhito Sako, Minoru Nakao, Philip S Craig. Widespread co-endemicity of human cystic and alveolar echinococcosis on the eastern Tibetan Plateau, northwest Sichuan/southeast Qinghai, China. PLoS Negl Trop Dis. 2011; 5(10): e1364.

Matsumoto J, Müller N, Hemphill A, Oku Y, Kamiya M, Gottstein B. 14-3-3- and II/3-10-gene expression as molecular markers to address viability and growth activity of *Echinococcus multilocularis* metacestode. Parasitology 132: 83–94, 2006.

Porot C, Wang J, Germain S, Seimbille Y, Camporese D, Knapp J, Vuitton DA, Blagosklonov O, Gottstein B: In vitro and in vivo investigations to develop functional imaging by Positron Emission Tomography (PET) for murine and human alveolar echinococcosis. Abstract book, 24th WAAVP Meeting, Perth, Australia, 2013.

Siles-Lucas S, Gottstein B. Review: Molecular tools for the diagnosis of cystic and alveolar echinococcosis. Trop Med Int Health 2001; 6: 463–475.

Siracusano A, Delunardo F, Teggi A, Ortona E. Host-Parasite Relationship in Cystic Echinococcosis: An Evolving Story. Clin Develop Immunol 2012; 639362, doi:10.1155/2012/639362.

Stojkovic M, Gottstein B, Junghanns T. Echinococcosis. In: Manson’s Tropical Diseases, 23rd edition. Eds: Farrar J, Hotez PJ, Junghanss T, Kang G, Lalloo D, White N. Elsevier Saunders, 2014; pp. 795–819.

Tappe D, Sako Y, Itoh S, Frosch M, Grüner B, Kern P, Ito A. Immunoglobulin G Subclass Responses to Recombinant Em18 in the Follow-Up of Patients with Alveolar Echinococcosis in Different Clinical Stages. Clin Vaccine Immunol 2010; 17: 944–948.

Yamasaki H, Nakaya K, Nakao M, Sako Y, Akira Ito. Significance of Molecular Diagnosis using Histopathological Specimens in Cestode Zoonoses. Trop Med Health 2007;35: 307–321.

Zhang W, Wen H, Li J, Lin R, McManus DP. Immunology and Immunodiagnosis of Cystic Echinococcosis: An Update. Clin Develop Immunol 2012; 101895, doi:10.1155/2012/101895.

##### L-10. Organization of echinococcosis care management in China and input of telemedicine. Analysis of telemedicine application in North-Western China for the diagnosis and treatment of human echinococcosis

Li Yong, Wen Hao, Xiu Yan, Sun Liang, Zhang Xi, Han Yuezhen

The First Affiliated Hospital of Xingjiang Medical University, Urumqi 830054, China

dr.wenhao@163.com

**Objective:** Tele-consultation services were developed in order to improve the general level of diagnosis and treatment of human echinococcosis prevention in Xinjiang and the Northwesten Regions in China.

**Methods:** Statistical analysis of 2,550 cases of human echinococcosis using tele-consultation from April 2008 to December 2013 in our hospital: number of consultations, consultation purposes, consultation characteristics, local differences and conversion rate to on-site consultations.

**Results:** 1.There was a trend of increase year by year: 38 cases in 2008, 134 cases in 2009, 345 cases in 2010, 482 cases in 2011, 678 cases in 2012, 873 cases in 2013. So far, the total number of consultations is 42,200; echinococcosis accounted for 6.04% of total tele-consultations. 2. The purpose of consultation for the diagnosis, treatment and defining the operation method, accounted for about 68.3% of the total echinococcosis consultations .Main aim of tele-consultation was solving the critical cases. The results show that the demand is beyond ordinary consultation mode, more high-end oriented and diversified mode. 3. Liver echinococcosis accounted for 99.45% of tele-consultations, then pelvic (0.24%), lung (0.19%), and cerebral echinococcosis (0.12%). It basically corresponded to the distribution of human echinococcosis. 4. The distribution of consultations in the region was non-uniform. Regions with the higher number of consultations include Altay, Changji, Kashgar and Aksu sub-regions, and Mongolian autonomous prefecture, which accounted for 72.6% of the total amount of consultation. 5. The transfer rate of remote consultation to the referral center was 8.03%; 91.97% of the patients with remote consultation effectively got appropriate treatment locally, on the site of their residence.

**Conclusions:** As our three-stage remote network system improves, there is an upward trend in the number of tele-consultations. The telemedicine demand is diverse. Telemedicine applications broaden from tele-diagnosis to remote education training, remote surgery teaching, guidance on drug treatment, follow-up of patients, remote online instruction, multicenter remote online communication and other high-end fields. It also deals with the local prevention and control: echinococcosis prevention platform, online assistance to sub-center case screening. Telemedicine actually strengthened regional echinococcosis scientific management, at the regional level.

##### L-11. Networking between hospitals and small health care units for the treatment of cystic echinococcosis in Morocco

Benazzouz Mustapha

Department of Medicine C, Ibn Sina Hospital Rabat, Morocco

benaz21@hotmail.com

Cystic echinococcosis is a medical and public health problem in Morocco. Management of this disease is one of primary public health problem in our country. The management of this disease is heterogeneous across the country. WHO-IWGE experts proposed a stage-specific approach which is not used in all provincial hospitals, and surgical treatment is still the main treatment. All therapeutic options are not available in small health care units, especially PAIR. For the classification, the most used is GHARBI classification; the WHO classification is not well known and not used in daily practice in this area. For all those factors we have created a networking between teaching hospital, provincial hospitals and small health care units. The objectives of this network are:

- Teach WHO CE ultrasound classification
- Diffuse WHO new guidelines for the treatment of CE
- Adapt these new guidelines to our context
- Teach and diffuse PAIR, mainly in endemic areas

Target physicians for this program are:

- General practitioners
- Gastroenterologists
- Radiologists
- Surgeons

All our activities are performed with the collaboration of:

- Direction of epidemiology and diseases control of
- National experts from teaching hospital
- Delegates of the ministry of health in endemic areas.

Recently with the collaboration of Ministry of Health of Italy, Ministry of Health of Morocco, Department of Medicine C, Ibn Sina Hospital Rabat, World Health Organization and WHO Collaborating Centre for Clinical Management of Cystic Echinococcosis at the University of Pavia this networking was reinforced. During this project proposed activities are as follows:

- Basic training of medical and paramedical personnel based in endemic area (Ifrane and El Hajeb), on its epidemiology, diagnosis, and on the criteria for the “rational selection” of the most appropriate treatment option
- Training of non-specialist medical personnel on ultrasound diagnosis and classification with implementation of criteria for the “rational selection” of the most appropriate treatment option
- Advanced training of hospital-based, specialist medical personnel based in target areas on percutaneous treatment (PAIR) of uncomplicated abdominal echinococcal cysts
- Basic education on CE of the local population.

##### L-12. Reference-centre network for the care management of alveolar echinococcosis: the FrancEchino and EchinoVista experience

Bresson-Hadni Solange, Grenouillet Frédéric, Knapp Jenny, Demonmerot Florent, Richou Carine, Vuitton Dominique A., Millon Laurence, the FrancEchino and EchinoVista networks

WHO-Collaborating Centre for Prevention and Treatment of Human Echinococcosis & Centre National de Référence “Echinococcose alvéolaire”, University of Franche-Comté and University Hospital, 25030 Besançon, France

dr.bresson.hadni@wanadoo.fr

The University Hospital of Besançon, located in the highest endemic area for alveolar echinococcosis (EA) in France, has been deeply involved in AE patient care for many years. During the past fifteen years, we have dedicated energy to set up a network of French physicians, based on 8 Reference University Hospitals located in endemic areas, aiming to optimize the care management of this severe parasitic disease in our country.

Historically, the first official network of physicians concerned by human AE care in France was included into the European network, EurEchinoReg, initiated in 1996, supported by the European Commission, DGXXIV (currently SANCO); its primary objective was to record human cases of AE, in order to better assess the epidemiology of the disease in Europe. European funds allowed us to initiate links between centres in charge of AE patients, i.e. 4 reference centres of the French endemic area, Nancy, Lyon, Clermont-Ferrand and Besançon. The European Commission support, maintained through the EchinoRisk project (DG RESEARCH) ended in 2002; each country had thus to find a solution to maintain case registration and to develop other types of activities, including coordinated management of patients. In France, the network, named “FrancEchino” could be maintained thanks to the financial support from the National Institute for Health Surveillance (InVS), as from 2004.

Most of the work performed by the network consisted in exhaustively collecting and recording AE cases in France, by crossing registration sources and actively retrieving cases. Epidemiological data in humans could thus be correlated to findings in animal hosts and progression of the disease towards western France, as analysed by the eco-epidemiologists of the network (academic teams and the inter-district organization against zoonoses, ELIZ) However, thanks to this case recording throughout years (retrospectively from 1982 to 1996, then prospectively), we became able to give useful information on clinical and therapeutic changes, and to observe if the current therapeutic attitudes well fitted with the recommendations of the WHO-Informal Working Group on Echinococosis (IWGE) published in 1996 and 2010. Because of its active involvement in the WHO-IWGE and networking activities, in 1995, our centre also received mandate by WHO to collaborate with this institution on the various issues dealing with prevention and treatment of echinococcosis.

The WHO-Collaborating centre through the FrancEchino network has served as a basis to disseminate the expertise gained in managing the highest number of patients with AE in France, on a multidisciplinary model. Clinicians (hepatologists, surgeons, digestive endoscopists), medical imaging specialists (radiologists, ultrasonographists, nuclear medicine specialists), medical biologists (parasitologists, clinical pharmacologists, immunologists) and pathologists, from Besançon University Hospital, and researchers working at Franche-Comté University, have informally ensured different missions for several years. Besides case recording and analysis, the first of these missions concerned expert advice for AE patients, especially for isolated physicians inside and outside the recognized endemic areas of France for *E. multilocularis* infection. That way, primary care hospitals and their physicians in the traditional and newer endemic zones were aggregated to the initial network. Progressively, this informal network has been institutionalized to become part of the first National Reference Centre (CNR) for Alveolar Echinococcosis formally qualified by the French Ministry of Health, its “hub” being located in the Dept of Parasitology of Besançon University Hospital. This recognition has strengthened the network and offered financial and human resources to optimize the links existing between different structures concerned by this disease and set up a quality control system for serology.

It has soon appeared that a real coordination of care management, at least at the inter-region level, was a relevant objective which would make prospective studies possible and would ensure a better and more equal access to care for AE patients. The EchinoVista project (“*Alveolar echinococcosis: Parasite viability and innovative markers for the follow-up of patients treated with Albendazole*”) made this objective realistic. It is a prospective clinical study, within the framework of the Hospital Clinical Research Program, supported by an inter-regional funding of the Ministry of Health. The project includes 6 French centers (Besançon, Dijon, Lyon, Nancy, Reims, and Strasbourg University Hospitals) and the Parasitology Institute of Bern, Switzerland, as partner for the development of serum markers. It has received ethical clearance from the Committee for the Protection of People of the Franche-Comté Region on March 1st 2012. Its main objective is to optimally manage AE patients treated with albendazole, and to make appropriate and timely decision of treatment withdrawal. It also includes an exploratory analysis of existing and newly developed biological and imaging exams, for diagnosis and follow-up, and a study of the relationship of these markers to the viability of the parasite and/or the activity of the parasitic lesions. Two groups of patients are prospectively included in the study a) patients with radical operation (liver resection), with the prospect of studying albendazole withdrawal after 1-yr of treatment on the basis of negative PET-CT and serology; b) patients without surgical operation, with the prospect of evaluating the possibility of albendazole withdrawal from the 4th year of treatment. Additional studies on albendazole pharmacokinetics, on cytokine and other immunological markers, and on the prognostic value of the respect of a “safety margin” by surgeons at operation, are associated with the prospective follow-up of patients. Until now, 21 patients (among them, 8 with radical operation), have been included in this project which is the first collaborative clinical study ever performed in France in the field of AE care management. A similar project is being set up at the first Affiliated Hospital of Xinjiang Medical University in Urumqi, China. “EchinoVista” may serve as a pilot project for a more ambitious multicenter European trial to explore new strategies of treatment and/or test new drugs.

**References**

Chauchet A, Grenouillet F, Knapp J, Richou K, Delabrousse E, Dentan C, Capelle S, Di Martino V, Deconinck E, Blagoskloonov O, Vuitton DA, Bresson-Hadni S, FrancEchino Network. Emergence of a new opportunistic infection in Europe: hepatic alveolar echinococcosis. A fifty-case report. J Hepatol 2013;58:S 381.

Combes B, Comte S, Raton V, Raoul F, Boué F, Umhang G, Favier S, Dunoyer C, Woronoff N, Giraudoux P. Westward spread of Echinococcus multilocularis in foxes, France, 2005–2010. Emerg Infect Dis. 2012 Dec;18(12):2059–62.

Crouzet J, Grenouillet F, Delabrousse E, Blagosklonov O, Thevenot T, Di Martino V, Piarroux R, Mantion GA, Bresson Hadni S. Personalized management of patients with inoperable alveolar echinococcosis undergoing treatment with albendazole: usefulness of positron-emission-tomography combined with serological and computed tomography follow-up. Clin Microbiol Infect. 2010 Jun;16(6):788–91.

Grenouillet F, Knapp J, Millon L, Raton V, Ricou C, Piarroux M, Piarroux R, Mantion G, Vuitton DA, Bresson-Hadni S. L’échinococcose alvéolaire humaine en France en 2010. Bull Epidemiol Hebd (Hors-série) 2010 : 24–25.

Kern P, Bardonnet K, Renner E, Auer H, Pawlowski Z, Ammann RW, Vuitton DA, Kern P; European Echinococcosis Registry. European echinococcosis registry: human alveolar echinococcosis, Europe, 1982–2000. Emerg Infect Dis. 2003Mar;9(3):343–9.

Piarroux M, Piarroux R, Giorgi R, Knapp J, Bardonnet K, Sudre B, Watelet J, Dumortier J, Gérard A, Beytout J, Abergel A, Mantion G, Vuitton DA, Bresson-Hadni S. Clinical features and evolution of alveolar echinococcosis in France from 1982 to 2007: results of a survey in 387 patients. J Hepatol. 2011 Nov;55(5):1025–33.

Piarroux M, Piarroux R, Knapp J, Bardonnet K, Dumortier J, Watelet J, Gerard A, Beytout J, Abergel A, Bresson-Hadni S, Gaudart J; FrancEchino Surveillance Network. Populations at risk for alveolar echinococcosis, France. Emerg Infect Dis. 2013 May;19(5):721–8.

Said-Ali Z, Grenouillet F, Knapp J, Bresson-Hadni S, Vuitton DA, Raoul F, Richou C, Millon L, Giraudoux P; Francechino Network. Detecting nested clusters of human alveolar echinococcosis. Parasitology. 2013 Nov;140(13):1693–700.

Vuitton DA, Wang Q, Zhou HX, Raoul F, Knapp J, Bresson-Hadni S, Wen H, Giraudoux P. A historical view of alveolar echinococcosis, 160 years after the discovery of the first case in humans: part 1. What have we learnt on the distribution of the disease and on its parasitic agent? Chin Med J (Engl). 2011 Sep;124(18):2943–53.

Vuitton DA, Zhou H, Bresson-Hadni S, Wang Q, Piarroux M, Raoul F, Giraudoux P. Epidemiology of alveolar echinococcosis with particular reference to China and Europe. Parasitology. 2003;127 Suppl:S87–107.

##### O-34. Current management of cystic echinococcosis; a survey of specialist practice

Nabarro Laura E, Chiodini Peter L

Hospital for Tropical Diseases, Mortimer Market Centre, London, WC1E 6JB, U.K.

laura.nabarro@nhs.net

**Background and aims:** Cystic echinococcosis (CE) is a significant public health problem around the world. Despite the WHO endorsed “Expert consensus for the diagnosis and treatment of cystic and alveolar echinococcosis” there remains minimal evidence to guide treatment. Our aim was to describe the current management of CE around the world.

**Method:** Clinicians interested in CE were identified by searching PubMed using the terms “cystic echinococcosis”, “hydatid” and “*Echinococcus granulosus*”. Infection and parasitology societies were contacted for emails of relevant clinicians. Using the online tool, SurveyMonkey, a questionnaire was produced detailing history, examination, serological results and imaging of 5 clinical cases. Clinicians were asked how to manage each case using tick boxes and short answer questions.

**Results:** 42 clinicians responded from 23 countries. *Patient 1:* Type 2 hepatic hydatid cyst. 65% performed surgery; 12.5% performed PAIR. PAIR is contraindicated in type 2 cysts (Expert consensus). Pre procedure drug treatment ranged from nil to 6 months; post procedure treatment ranged from 3 to 24 weeks. *Patient 2:* Disseminated CE with abdominal, pelvic and pleural disease. Medical management with albendazole preferred (64.7%) but 71% of requested surgical review. Length of medical treatment varied from under 3 months (12%) to life-long treatment (24%). *Patient 3:* Type 3A hepatic cyst which had eroded into the lung. 70% opted for surgery; 20% performed PAIR (contraindicated in pulmonary disease). Drug treatment ranged from nil to one month before procedure and from one to twelve months after procedure. *Patient 4:* Type 2 cyst with rupture into biliary tree. History of acute hepatitis secondary to albendazole. 67% performed surgery; 14% percutaneous procedure; 4% watch and wait. Many were cautious with albendazole, starting at low dose; others used praziquantel as sole agent (15%) or mebendazole (10%). Of concern, 55% inserted a scolicidal agent into the cyst despite connection with the biliary tree, putting patients at risk of sclerosing cholangitis. *Patient 5:* Both pulmonary and hepatic cysts. 82% performed surgery; 7% suggested PAIR or drug therapy alone. Albendazole is the most commonly used drug in CE. Worryingly, some clinicians still use interrupted courses. Mebendazole used by some but at doses far below the recommended 50 mg/kg/d. Praziquantel used infrequently as a synergistic agent in type 2 cysts (10%); increasingly in patients with disseminated disease (29%) or drug reactions (15%). Doses ranged widely from 100 mg BD to 40 mg/kg once per week (Expert Consensus) to 50 mg/kg/d.

**Conclusion:** Current management is extremely diverse with a number of common but unsafe practices. The Expert Consensus lacks clarity; needs rewriting to be more prescriptive on drug doses and duration of treatment. A pan-European CE registry should be established for prospective data collection. Clinical trials are needed to look at the length of albendazole treatment, role of praziquantel and role of percutaneous procedures to spare surgery.

##### O-35. Albendazole efficacy in cystic echinococcosis: how does current evidence translate into practice?

Stojkovic Marija

Section Clinical Tropical Medicine, Heidelberg University Hospital, Germany

marija.stojkovic@med.uni-heidelberg.de

Metaanalysis of studies published on albendazole (ABZ) is precluded by the heterogeneity of inclusion criteria, dosage and duration of treatment, outcome measures, and follow-up of patients. We therefore initiated EchinoMEDREV, a collaborative effort of CE specialists, to collect individual patient data from patients treated with benzimidazoles.

The main findings of our study on which treatment decisions can be built are: a) around 50% – 75% of CE1 cysts smaller than 6 cm in diameter show inactivation 1–2 years after ABZ treatment and b) CE 2 and CE3 are rather unresponsive.

With a 50–75% chance of cure (permanent inactivation of cyst) these findings are of individual patient and public health relevance. In individual patient care a 2 step approach is worth considering with an around 1:2 chance to be cured after a single course of ABZ treatment. Important in this approach is that the limits are clearly set. We suggest as a rule a single 3 months course of ABZ and final assessment of cure by ultrasound one year after completion of treatment. In patients not cured by ABZ alternative treatment options are considered in a second step. The public health benefits are obvious with regard to savings on health service resources and cost.

### 3.D. Clinical cases and series

#### Posters

##### P-52. Surgical management of bilateral pulmonary hydatid cysts

Achour Karima, Laribi Abdesslam, Nekhla Ahmed, Dehal Siham, Ghebouli Noureddin, Ameur Soltane

Mustapha University Hospital, Algiers, Algeria

achour.karima@gmail.com

**Objectives:** Hydatidosis is a parasitic disease common in Mediterranean countries; the most often affected organs are the liver and the lungs. It’s usually asymptomatic and discovered incidentally. However there are a number of patients who present with bilateral forms and will require a complex management with significant morbidity and mortality rates .The purpose of this work was to report the specific management of these complicated forms. It should be considered that there are many situations with no consensus, such as: conventional regular surgery or not, approaching the bilateral cysts in one session or two, place of pleurectomy for intrapleural ruptured cysts, and timeline in the management for the associated KHP and liver location . The important criteria are a more simple immediate postoperative course and less long term recurrence.

**Methods:** We reviewed the records of 2’041 patients operated on for pulmonary hydatid cyst in our department during a 30-year period (1983–2012).

**Results:** The PHC essentially affected young men. 9.16% had PHC scattered throughout both lungs, including 63 patients (3.07%) had pulmonary extra localization (mainly liver). 81.28% of patients (*n* = 152) benefited two-stage surgery. In our series, the morbidity was 19% and the mortality was 2.67%.

**Conclusion:** The PHC is a surgical pathology whose prognosis depends fundamentally on the stage of evolution, the cysts location, the number of cysts, and the quality of the initial treatment.

##### P-53. Cardio-vascular cystic echinococcosis: success and limits of the diagnosis and treatment*

Cretu Carmen-Michaela^1,2,4^, Smarandita Lacau Ioana^3^, Mihailescu Patricia^4^, Chiriac Babei Catalin^1,5^, Popa Loredana Gabriela^1,2^

^1^University of Medicine and Pharmacy “Carol Davila”, Bucharest, Romania

^2^Colentina Clinical Hospital, Bucharest, Romania

^3^Hiperdia Medical Centre, Elias Hospital, Bucharest, Romania

^4^Eco-Para-Diagnostic Laboratory, Bucharest, Romania

^5^Emergency Clinical Children Hospital Grigore Alexandrescu, Bucharest, Romania

michaelacarmen.cretu@gmail.com

**Background:** Cystic echinococcosis (CE), due to accidental development in human body of cestoda larval stage of *E. granulosus,* parasite of the dog, is quite common in Central and Eastern Europe. Romania is a territory with many cases of CE, but the real epidemiological parameters (incidence, prevalence) remain unknown. The most common locations of the cysts are liver and lung, but less common locations are present in different organs, including cardiovascular.

**Methods:** a retrospective analysis of 9 CE cases with cardio-vascular location, out of 1164 CE cases admitted in Colentina Clinical Hospital, Parasitology Department, during 1995–2013.

**Results:** 9 cases of cardiovascular CE (0.77%) were found out among patients registered during 1995–2013, one associating a vascular dissemination. Imaging diagnosis consisted in abdominal and heart US, chest X-ray, thorax and abdomen CT and IRM. Serology by ELISA: positive in 7 cases. Positive Western Blot increased accuracy of diagnosis in 1 case. Discovery of CE was fortuitous in 6 cases (primary location) and during post-surgery follow-up in 3 cases (recurrence). Single cysts were present in 8 cases; one case presented multiple pericardial and atrium cysts. Heart was the only organ involved in 3 cases, and associated with liver and lung cysts in 4, and 2 cases respectively. Location was pericardium in 4, inter-ventricular septum in 2, inter-atrium septum in 1, and intra-atrial cavity in 2 cases. Surgical interventions followed by albendazole were recommended in 4 cases and medical treatment with albendazole as single therapeutic alternative in 5 cases (1 case refused surgery and in 4 cases surgery was delayed or reconsidered). Satisfactory course and prognosis were noticed in 6 cases; in 2 cases death of the patients occurred during or immediately after surgery. One patient died of sudden death. Three clinical cases will be presented: A 12-years old boy with heart and liver CE, with a very good evolution following albendazole therapy; A 32-years old male, with multi-organs CE (both lungs, mediastinum, pericardial and atrial locations), with vascular dissemination (popliteal artery occlusion due to membranes and thrombi) with good evolution following albendazole treatment; A 28-years old female with liver and pericardial locations of CE, who developed albendazole intolerance.

**Discussion:** in endemic areas for CE, heart locations can be disclosed; they are often associated with other locations. Full check-up of all new CE cases is recommended. Evolution of cases remains unpredictable, but albendazole is recommended in all cases, either as single therapeutic alternative or associated to surgery, even if, for certain reasons, surgical removal of the cyst should be delayed.

*Work funded by FP7 Project – 602051.

##### P-54. Multivisceral hydatidosis, diagnosis and treatment challenges*

Popa Gabriela Loredana^1,2,3^, Popa Alexandru Cosmin^1,2^, Mastalier Bogdan^1,2^, Tanase Iulia^4^, Mihailescu Patricia^3^, Popa Mircea Ioan^1,2^, Cretu Carmen Michaela^1,2,3^

^1^Colentina Clinical Hospital (CDPC), Bucharest, Romania

^2^University of Medicine and Pharmacy “Carol Davila”, Bucharest, Romania

^3^Eco-Para-Diagnostic Laboratory, Bucharest, Romania

^4^Hiperdia Medical Centre, Elias Hospital, Bucharest, Romania

michaelacarmen.cretu@gmail.com; dr.gabriela.popa@gmail.com

**Background:** Cystic echinococcosis (CE) is a zoonosis caused by the larval stage of *Echinococcus granulosus*, being considered as a neglected disease. Hydatidosis can be asymptomatic for a long time. It could be detected late, when the hydatid cyst are large, with risks of complication and a reserved prognosis. In our country an important number of cases were registered in the last years.

**Methods:** We followed the WHO recommendations regarding the medical attitude and therapeutic strategy. Our aim was to evaluate the multivisceral hydatidosis in Romania, in order to demonstrate the importance of epidemiological, clinical and laboratory data, and to present some of the most relevant cases managed by our team.

**Results:** During the last 18 years, our team diagnosed and treated 1,164 cases of cystic echinococcosis. Out of them, for more than 72% the localization was unique, while 4.64% were multivisceral hydatidosis (54 patients). We registered relapses in 31.8% of the cases at one year, and in 20.4% of the cases after more than 4 years. The diagnosis was based on a combination of methods (imaging techniques, serology tests) depending on the localization of CE. Most of these cases were admitted to our clinic without a previous diagnosis and/or treatment, some of them presenting complications at the admission date. The treatment (surgical, medical, or combined) was adapted from one case to another. Three of the most illustrative cases will be presented.

**Discussion/Conclusion:** Hydatid disease represents an important issue for the individual, and a public health concern. Hydatidosis often has a subclinical or asymptomatic evolution, leading to the risk of grave, sometimes life-threatening complications. Interdisciplinary cooperation is very important in order to have the positive diagnosis and to implement the most appropriate treatment strategy. Every hydatid cyst evolves as an independent entity; therefore the treatment (and the number of treatment courses) will depend on the particularities of each case.

*Work funded by FP7 Project – 602051

##### P-55. Two cases of femoral hydatidosis treated by albendazole and prosthetic reconstruction

Dupouy-Camet Jean, Leslé Florence, Magrino Baptiste, Sailhan Frédéric, Yera Hélène, Larousserie Frédérique, Rouquette Alexandre, Anract Philippe

Departments of Parasitology, Orthopaedic Surgery & Pathology, Hôpital Cochin, Assistance Publique Hôpitaux de Paris, Paris Descartes University, Paris, France

jean.dupouy-camet@cch.aphp.fr

**Background:** Osseous locations are severe and recurrent complications of hydatidosis. We report here two cases illustrating the severity of this invasive and destructive osseous parasitosis located at the femur and the hip joint, which required extensive resection and prosthetic reconstruction.

**Case reports:** The first case had a long history of liver and lung hydatidosis, only treated surgically. He finally was referred to us for persistent and increasing pain in the right thigh. Radiograph of the pelvis showed a thinned and eroded medial cortex of the proximal right femur. Punctuated osteolysis was seen in the right greater trochanter and the coxo-femoral joint showed a concentric narrowing. The anterior and superior compartment of the femoral head also showed partial collapse and was treated by a wide “en-bloc” extra-articular resection of the right hip joint including the proximal femur associated with a reconstruction massive cemented total hip replacement prosthesis (“Saddle prosthesis”) with a proximal femur allograft. Twenty-six months post operatively, this patient came back presenting pain in the right lower limb. Radiograph of the femur showed osteolytic changes in the proximal part of the remaining femur suggesting local recurrence. The first prosthesis was changed for a longer femoral stem. Histological examination of the excised tissues confirmed the recurrence showing typical lamellar membranes. The second case experienced a sudden excruciating pain in the left femur after getting out of her car. Radiographs revealed a pathological femoral shaft fracture of the left femur. A surgical biopsy was performed and hooks and protoscoleces were seen by the unstained microscopic examination of the removed tissues. Histological analysis showed laminated layers. Considering the extension of the hydatid lesions within the two-thirds of the femur diaphysis “en-bloc” total femur resection was performed and a total femur endoprosthesis replaced the patient’s femur. High titers of specific antibodies were detected in both cases by ELISA and western blot. Preoperative and postoperative chemotherapy with albendazole (associated with praziquantel for the first case) was prescribed.

**Discussion/Conclusion:** Owing to the high occurrence of recurrences a long-term treatment, even up to several years, by albendazole, with regular monitoring of blood levels, should be considered. The treatment could be stopped as soon as the serodiagnosis of hydatidosis becomes negative. These two cases occurred several years after exclusive surgical treatments of recurrent lung or liver hydatidosis and might have been prevented if chemotherapy by albendazole had been initially applied.

##### P-56. Long-term follow-up of patients with alveolar echinococcosis in Germany

Gruener Beate^1^, Kern Petra^2^, Mayer Benjamin^2^, Muche Rainer^2^, Kern Peter^1^

^1^Department of Infectious Diseases and Clinical Immunology, University Hospital, Ulm, Germany

^2^Department of Epidemiology and Medical Biometry, Comprehensive Infectious Diseases Center, Ulm University, Ulm, Germany

beate.gruener@uniklinik-ulm.de; petra.kern@uni-ulm.de; peter.kern@uni-ulm.de

**Methods:** From 1992 to 2011, 312 patients with alveolar echinococcosis (AE) were diagnosed and treated at the specialized outpatient clinic of the Ulm University. Demographic and clinical data were assessed and updated from the patients’ first visits until December 2012 (end of follow-up).

**Results:** At that time, 233 patients (74.7%) were alive, 40 (12.8%) had died, and 39 (12.5%) were lost to follow-up. Patients were treated either by surgery with subsequent benzimidazole prophylaxis for at least 2 years (*n* = 133), or continuous benzimidazole treatment in case of inoperability (*n* = 157). At first diagnosis, 17 patients had inactive lesions (possible cases; Brunetti et al., Acta Trop 2010, 114:1-16); 2 of them converted to an active stage 2.3 and 5.3 years after diagnosis. Imaging and treatment schemes changed during the 20 years of observation. AE was diagnosed more often by chance in patients from 2000 onwards (48.0%) than before 2000 (28.7%). Since 2000, the disease was detected more frequently with lesions at PNM stages I and II (27.0% vs. 15.8%) according to WHO classification (Kern et al. Parasitol Int 2006, 55 Suppl: S283–287); as a consequence, radical resections were feasible in more patients (57.7% vs. 20.0%). Surgical resections were less frequent since 2000 (38.2% vs. 50.5%) as were modifications of the medical treatment (29.7% vs. 59.6%) during follow-up.

Since 1993, PET-CT-scans with ^18^F-FDG were used routinely to visualize larval activity at time of diagnosis. For follow-up, the rationale for performing a PET-CT every other year was to monitor the effect of continuous benzimidazole treatment, and to detect relapses after surgery. At the end of follow-up, medical treatment had been interrupted for 25.3% of the patients, as warranted by a lack of ^18^F-FDG uptake in the liver at two consecutive scans, normal levels of inflammation parameters, and other criteria (see WHO treatment recommendations, Brunetti et al. Acta Trop 2010, 114:1-16). Benzimidazole interruption was possible for 46.6% of the patients with surgery and 10.8% of the patients with medical treatment alone. Of 56 patients with R0 resection, 42 (75%) had stopped medical treatment. At their last visit, the disease status of 73.1% of the patients was judged as stable, in 5.1% as progressive while under medical treatment. AE was considered as being cured in 15.7% of the patients (treatment interrupted). The 5- and 10-years’ survival rates in this cohort were 96.8% and 90.5%.

**Conclusions:** Data analysis of two decades’ experience in the management of AE showed that best care can be provided to the patients when they present at an early stage of the disease. Staging is the prerequisite for a structured therapeutic approach. As the disease is rare, expertise is best acquired in a single specialized institution; a benefit for the patients results from strict adherence to the WHO treatment recommendations.

##### P-57. Splenic hydatid disease recorded in Riga Pauls Stradins clinical university hospital, Latvia: a case report

Pakalinsķe Māra^1^, Krūmiņa Angelika^2^, Strumfa Ilze^3^

^1^Daugavpils University, Riga, Latvia

^2^Infectiology and Dermatology Department, Riga Stradins University, Institute of Pathology, Riga, Latvia

^3^Pauls Stradins Clinical University Hospital, Riga, Latvia

s.laivacuma@inbox.lv

**Background:** Hydatid disease is a zoonotic infection caused by *Echinococcus* spp, where the worldwide distributed *E.granulosus* is mostly responsible for it. The hydatid cyst of the Echinococcus larval stage develops in the liver more often than in any other part of the human body. Lungs are considered to be another important organ affected, but splenic hydatid disease is considered to be rare among patients.

**Clinical observation:** In the present case we decided to focus on one hydatid disease’s patient from Riga Pauls Stradins Clinical University Hospital which had an Echinococcus cyst removed from her spleen. From year 2003 till 2013 this was the only case of splenic hydatid disease in this hospital (one out of 38 other echinococcosis cases). There are no records of a cyst in the liver of this patient in the Riga Pauls Stradins Clinical University Hospital. Splenectomy was performed to the 29 years old woman and a cyst of the size 7.5 × 4.5 × 4 cm was removed 3 cm from the hilum lienis. Spleen echinococcosis was confirmed in the patient with the help of histological examination.

##### P-58. Surgical treatment of pulmonary echinococcosis in children

Namazova-Baranova Leyla^1^, Morozov Dmitriy^1^, Goremykin Igor^2^, Gorodkov Sergey^2^, Haspekov Dmitriy^3^, Gusev Alexey^1^

^1^Scientific Center of Children Health (SCCH), Moscow, Russia

^2^Saratov State Medical University, Moscow, Russia

^3^Children’s Clinical Hospital of St. Vladimir, Moscow, Russia

DrGusev@yandex.ru

**Backround:** Lung echinococcosis is supposed to be rare in the pediatric practice.

**Patients and results:** We compared the surgical treatment results of 74 patients with pulmonary echinococcosis in endemic (Saratov region, close to Kazakhstan Republic) and non-endemic (Moscow) areas of the Russian Federation. Thoracotomy level and access varied depending on the location of the cyst. Thoracoscopic removal of the right lung’s lower lobe with a cyst was performed in 3 children. Ideal cystectomy was performed in 29 children. In 26 children cyst removal was performed after puncture, aspiration of parasitic content and protoscolecide exposure for 10–15 minutes. In the case of active perifocal process the resection of the lung tissue was required: in 9 cases, segment resection, in 15, lobectomy, in 4 cases, pneumonectomy. The pleural cavity drainage was done in 35 cases. In patients with combined lung and liver damage (hepatic cysts on diaphragmatic surface) – echinococcectomy was performed by simultaneous liver cysts’ phrenotomy (in 6 children). In bilateral pulmonary involvement the echinococcectomy carry out on the opposite side was used after 3 to 6 months; in one case both sides were operated on during the same surgical session. No deaths were observed.

**Conclusions:** Due to the relative increase of the disease prevalence during last years, a European network is needed with the function of enhancing detection, early diagnosis and active introduction of minimally invasive treatment possibilities among doctors and patients with lung echinococcosis.

##### P-59. Pelvic bone and hip joint hydatid disease misdiagnosed as tuberculosis: a clinical case

Laivacuma Sniedze, Krumina Angelika, Viksna Ludmila

Riga Stradins University, Riga, Latvia

s.laivacuma@inbox.lv

**Background:** Echinococcus can affect virtually any organ and bone echinococcosis is considered as rare, 0.5–2.5% of all cases. Most of the bone echinococcosis cases are difficult to diagnose, they usually are incurable and have a high level of recurrence.

**Methods:** Presentation of a patient with unusual localization of echinococcosis to highlight the diagnostic difficulties in countries where the disease is not widespread.

**Results:** A 43-year old woman was diagnosed with lymphatic node tuberculosis in the age of 37, later in the same year she had complaints of growing pain in the left hip joint and it was presented as tuberculous process. During the next 6 years she received several courses of antituberculosis medication and underwent 12 left hipbone and hip joint surgeries for the with no real improvement as the destructive process in the above mentioned localization continued involving left side of pelvis as well. According to anamnesis there was a lesion in the left lung, interpreted as tuberculoma.

After those 6 years of unsuccessful treatment she came to the doctor with complaints of difficulty to walk because of the pain and discharge from active fistula, CT scan showed massive destruction of left side of pelvis reaching sacroileal joint, with masses of variable density. Surgery was performed and the finding in the material was necrotizing granulomatous inflammation with atypical structure of granuloma, necrobiotically changed chitin shells and several intact chitin sacs. Serologic testing was performed confirming Echinococcus granulosus infection. Patient received treatment with albendazole. Six moths later due to growing pain in the left hip she was admitted in the hospital, during the investigation the lung CT scan was performed which showed multiple lesions in both lungs, the biggest of them in left lung previously interpreted as tuberculoma. Diagnostic VAT (video assisted thoracoscopy) was performed in order to take out the lesion and in morphologic analysis echinococcus chitin shells were found. At that time the medical documentation for the previous years was reviewed and it was found that the diagnosis of tuberculosis had been based only on morphologic findings of granulomas in the material. So it is possible that initially the destructive process in the bone was confused by Echinococcus granulosus infection based on the constant progression of the destruction and no effect from the treatment.

**Discussion/Conclusions:** This case illustrates the difficulties of diagnosing echinococcosis in those countries where it is not very common so the alternative diagnosis usually is considered more possible. But recognition of this parasitosis is extremely important to avoid unnecessary treatment and development of physical disability.

##### P-60. Clinical analysis of surgical treatment for human hepatic cystic and alveolar echinococcosis

Shao Yingmei, Aji Tuerganaili, Jiang Tieming, Ran Bo, Wen Hao

Hepatobiliary & Hydatid Department, Digestive and Vascular Surgery Centre, First Affiliated Hospital, Xinjiang Medical University, Urumqi, China

syingmei3000@163.com

**Background:** We used retrospective hospital clinical data to evaluate and discuss the indications and efficacy of the various surgical techniques in cystic and alveolar echinococcosis (CE and AE).

**Methods:** A total of 1’356 CE cases and 128 AE cases who underwent surgery from 2002 to 2012 in first affiliated hospital of Xinjiang medical university were retrospectively evaluated. Preoperative diagnosis, surgical procedures, demographic and clinical characteristics and postoperative follow-up were categorized and compared in the current study.

**Results:** 1.CE group: classic endocystectomy (Group A) was the most widely used surgical procedure. However, the incidence of postoperative complications was significantly lower (*p* < 0.01), and drainage time and post-operative hospital stay were significantly shorter in the total cystectomy group (Group B) than in groups undergoing other surgical procedures (*p* < 0.01). Among radical operations, total cystectomy had lower operation time and fewer complications (blood loss, post-operative liver dysfunction) than liver resection (*p* < 0.01). In our study, 1’355 CE cases (99.9%) were cured and only 1 case (0.1%) died peroperatively. 2. AE group: The 10-years survival of the radical resection of AE cases was 100%, and the 10-years survival of palliative procedure of AE cases was 44% (*p* < 0.01).

**Conclusions:** 1.Total cystectomy can be considered as a radical and practicable surgical method for the treatment of human CE, with better results in terms of control of CE recurrence, biliary leakage and cavity-related complications than the classic endocystectomy; it looks also safer when compared with liver resection. 2. Radical extended hepatectomy with biliary reconstruction can be considered as one of the effective surgical procedures for the treatment of human AE cases.

##### P-61. Diagnosis and treatment for biliary complications of hepatic cystic echinococcosis

Aji Tuerganaili, Shao Yingmei, Ran Bo, Jiang Tiemin, Wen Hao

Hepatobiliary & Hydatid Department, Digestive and Vascular Surgery Centre, First Teaching Hospital, Xinjiang Medical University, Urumqi, China

tuergan78@sina.com

**Background:** Our aim was to evaluate the effect of different diagnosis and treatment methods in hepatic cystic echinococcosis (CE) with biliary complications.

**Methods:** The data of 284 hepatic CE patients with biliary complications, surgically treated from January 2002 to January 2010 in first affiliated hospital of Xinjiang medical university, were analysed.

**Results:** (1) The diagnosis of biliary complications of hepatic hydatid cyst was difficult on ultrasound and CT, with sensitivity rates of 78.4% and 85.7%, respectively. MRCP was an effective, noninvasive and useful diagnostic tool in difficult cases; ERCP was used as the gold standard in confirmation. Biliary fistulae were seen in 3 patients (10.7%) treated by suturing the rupture site. In the non-sutured group, 17 patients (74%) developed biliary fistulae after surgery (*p* < 0.01). In three patients the fistula was a high-output type (the fistula output was greater than 250 ml/d). (2) CE communicated with the bile duct and (or) was super-infected in 210 patients.The cavity-related problems and draining time in group C (no bile duct exploration and decompression) were significantly higher than group A (biliary system explored and decompressed through the cystic duct) and group B (biliary system explored and decompressed through the common bile duct), while cavity-related problems and draining time between the A and B groups showed no significant difference. Biliary tract-related problems in group A were significantly lower than group B (*p* < 0. 05).

**Conclusions:** (1) MRCP was an effective, noninvasive and useful diagnostic tool; ERCP was used only as the gold standard in confirming intrabiliary rupture of liver cystic hydatid disease, and also as an effective technique for treating extended postoperative external biliary fistulae. (2) This study indicated that suturing the communication at the rupture site and biliary decompression were effective with low morbidity and mortality rates. (3) Cholangiography and common bile duct exploration through the cystic duct could solve the cavity-related problems while avoiding the T-tube related problems.

##### P-62. Anesthesia during surgical treatment of cardiac and pericardial echinococcosis: report of 18 cases

Yu Xiangyou^1^, Wang Yi^1^, Zhong Hua^1^, Wen Hao^2^

^1^Department of Critical Care Medicine, First Affiliated Hospital of Xinjiang Medical University, Urumqi, 830054 China

^2^Xinjiang Key Laboratory of Echinococcosis, First Affiliated Hospital of Xinjiang Medical University, Urumqi, 830054 China

yu2796@163.com

**Background and aims:** To investigate the effects of anesthesia during the surgical treatment of cardiac and pericardial echinococcosis.

**Methods:** Anesthesia during the surgical treatment of 18 patients (12 males and six females) with cardiac and pericardial echinococcosis in our hospital from 1978 to 2011 was analyzed retrospectively. The average age was 26 years (range, 8–56 years). Cysts were located on the pericardium in seven patients, on the myocardium in seven patients, on the cardiac chamber in three patients, and on both the myocardium and pericardium in one patient.

**Results:** Four patients were treated with cardiopulmonary bypass techniques, and others were treated without cardiopulmonary bypass. Blood gases, ion concentrations, urine volumes, and hemodynamics were normal and stable during the operations. Assisted respiration was used after surgery. The 18 patients recovered and were discharged with no anesthesia-related complications or allergic reactions.

**Conclusions:** Anesthesia during the surgical treatment of cardiac or pericardial echinococcosis is difficult. More attention should be paid to the choice of drugs and the maintenance of anesthesia. The management of respiration and circulation and the prevention of systemic allergies during the operation are very important.

##### P-63. Cystic and alveolar echinococcosis in the Czech Republic: diagnostics and follow up*

Stejskal Frantisek^1,2,3^, Trojanek Milan^2^, Oliverius Martin^3^, Kolbekova Petra^4^, Kolarova Libuse^4^

^1^Deparment of Infectious Diseases, Regional Hospital, Liberec, Czech Republic

^2^1^st^ Department of Infectious Disease, 2^nd^ Medical Faculty, Charles University and Hospital Na Bulovce, Prague, Czech Republic

^3^Transplant Surgery Department, Institute of Clinical and Experimental Medicine, Prague, Czech Republic

^4^Institute of Immunology and Microbiology, Prague, Czech Republic, 1^st^ Medical Faculty, Charles University, Prague, Czech Republic

fstej@lf1.cuni.cz; milan.trojanek@bulovka.cz; maol@ikem.cz; libuse.kolarova@seznam.cz

**Background:** Cystic echinococcosis (CE) represents a common parasitic liver infection imported to the Central and Western European countries from endemic regions. Alveolar echinococcosis (AE) is being recognized as an emerging infection in the Czech Republic (CZ), however, the national surveillance program has not been established yet.

**Methods:** We are presenting an overview of CE and AE cases diagnosed and treated at Hospital Na Bulovce in Prague and at Regional Hospital in Liberec.

**Results:** There have been treated 10 cases of CE at our departments since 2005. Eight cases were diagnosed in migrants from endemic regions: Bulgaria (2x), Romania, Russia, Kazakhstan, Montenegro, Tajikistan, and Uzbekistan and two in Czech citizens. A 36-year old Czech man presented with two subcapsular liver cysts in S4 and S2 regions and a single cyst in the lingula of left lung. He lived in CZ and reported history of short-term visits of Bulgaria, Russia and Mongolia since late 1990s. A 64-year female acquired the liver infection in CZ, probably. She lived in the rural region in Eastern Bohemia and did not travel to the CE endemic countries. The CE cysts have been identified in liver in 8 patients, in lungs only in 1 case, and in both liver and lung in 1 patient. The surgery and subsequent albendazole treatment underwent two patients with pulmonary CE and five patients with liver CE. One patient was treated with albendazole only and watch and wait approach was used in two patients. Currently, the recurrence of liver cysts appeared in three patients with liver CE 4–13 years after primary surgery despite pre- and postoperative albendazole treatment. All patients tolerated albendazole without adverse reactions, except for one with significant elevation of liver function tests. Currently, 4 female patients (32–66 years of age) with advanced inoperable liver AE are in long-term follow-up at our departments. Due to the disease progression and local complications one patient has undergone the orthotopic liver transplantation recently. All our patients are on long-term continuous therapy with albendazole that is well tolerated. Specific anti-echinococcal antibodies were presented in all our patients with both CE and AE at the time of diagnosis.

**Conclusion:** AE is endemic in CZ and there are reported increasing numbers of AE cases in Europe. In our country there have been diagnosed at least 15 autochtonous cases of AE during 2007–2013. Unlike CE, AE is still unfamiliar to our clinicians and the majority of Czech cases have been diagnosed histologically after surgery of liver mass process suspected from malignancy.

*This presentation was partially supported by the Scientific Board of the Regional Hospital Liberec and by grant PRVO-UK of Charles University in Prague.

##### P-64. Human cystic echinococcosis in Bulgaria (2008–2012): a retrospective study of some epidemiological characteristics and approaches in diagnosis and treatment

Marinova Irina^1^, Jordanova Diana, Harizanov Rumen, Rainova Iskra, Kaftandjiev Iskren, Tsvetkova Nina, Muhtarov Marin^2^

^1^National Centre of Infectious and Parasitic Diseases (NCIPD), Sofia, Bulgaria

^2^Department of Gastroenterology; MHAT – Kardjali, Kardjali, Bulgaria

marinova@ncipd.org; mukhtarov@abv.bg

**Background:** Bulgaria is the most affected country with cystic echinococcosis (CE) among the member-states in European Union.

**Methods and Results:** In the country, CE is a notifiable disease. For the period 2008-2012, the number of officially registered patients with CE was 1806 – newly diagnosed were 1627 (90%) and 179 (10%) were with relapses. The number of children was 295 (16.3%). This fact indicates the presence of a very active transmission of the parasite from main reservoir hosts to humans. According to the cyst location 70% of the patients (1264) were with hepatic echinococcosis, 17.5% (317) were with pulmonary echinococcosis and 12.5% (225) – with rare locations (kidney, spleen, brain, abdominal cavity).

For the studied period, 1490 patients were examined for CE at the National Centre of Infectious and Parasitic Diseases (NCIPD). The most common reason for examination was a cyst in the body, visualized with an imaging technique – 976 patients (65.5%). Follow-up serological tests were conducted in 387 (26%) patients treated for CE. In 23 patients (1.5%) the reason for examination was allergic symptoms. For reference diagnosis 104 patients (7%) with positive results in other laboratories but lack of a cyst in the imaging techniques were examined at NCIPD. The sera of all 1490 patients were tested with ELISA. Western blot was used in 130 cases with cysts and borderline results in ELISA or in cases with positive or borderline serology and undetectable cyst.

For the period 2008–2012, 373 patients were sent for treatment with albendazole at NCIPD – in 339 patients who initially were treated by surgery or PAIR was conducted anti-relapse treatment with albendazole and 34 patients contraindicated for surgery or PAIR were treated with albendazole only. The National Health Insurance Fund provides coverage of the albendazole therapy for each patient after approval from appointed commission at NCIPD. The duration of albendazole therapy varied usually between 2 to 4 monthly courses but in some patients treated with albendazole only, up to 8–16 courses were administered.

##### P-65. Primary extrahepatic Alveolar Echinococcosis in the sternum and the cervical spine of a chimpanzee (*Pan troglodytes*)

Federer Karin^1,4^, Hammer Sven^2^, Steinmetz Hanspeter^3^, Sydler Titus^4^, Deplazes Peter^5^

^1^Walter Zoo, Rapperswil, Switzerland

^2^Tierpark Görlitz, Rapperswil, Switzerland

^3^Knies Kinderzoo, Rapperswil, Switzerland

^4^Institute of Veterinary Pathology, University of Zürich

^5^Institute of Parasitology, University of Zurich, Zürich, Switzerland

karin.federer@walterzoo.ch

**Clinical observation:** A 32-year-old female chimpanzee (Pan troglodytes) from a zoo in eastern Switzerland was presented with recurrent skin abscesses in the upper sternum area. Otherwise, the chimpanzee was in good health with body condition and hematologic and serum biochemical variables within reference intervals. A computed tomography (CT) study of the chest revealed a bone swelling of the entire sternum and the first right rib with loss of normal bony structures. There were no abnormal diagnostic findings in other bones or soft tissues. For further clarification, an ultrasound-guided biopsy of the first right rib was performed. Histologic examination revealed the presence of granulation tissue. No other abnormal findings were obtained. Bacteriological cultures revealed unspecific bacterial growth. Bacterial osteomyelitis was suspected and a six-month course of antibiotic treatment was initiated, which however did not result in resolution of clinical signs. During the time of treatment and the following 1.5 years, recurrent small skin abscesses developed and drained spontaneously. Two years later, the chimpanzee became weak and developed a paresis of the right leg. About two weeks later, full paralysis of both legs and weakness in both arms with neurological deficits developed. Because of the poor prognosis, no further examination was performed and the chimpanzee was euthanized.

Post-mortem examination revealed a high-grade multifocal granulomatous osteomyelitis of the sternum, the first right rib and the seventh cervical vertebra, interspersed with vesicles delineated by Periodic-Acid-Schiff (PAS)- and Em2 (immunohistochemistry)-positive laminated layers, suggesting an infection with *Echinococcus multilocularis*. A PCR analysis detected *Echinococcus*-specific nucleic acids in the seventh cervical vertebra, providing proof of an infection with *E. multilocularis*. No abnormalities were found in liver and lungs.

**Discussion/Conclusion:** To the authors’ knowledge, there are no reported cases of alveolar echinococcosis (AE) in chimpanzees. Limited published information is available about AE without liver involvement in humans. Osseous affection is uncommon, occurring in only 1% of all cases. This case described an unusual and extremely rare circumstance involving a primary extrahepatic manifestation of AE in a chimpanzee.

##### P-66. The role of emergency surgery in hydatid liver disease

Yahya Ali Ibrahim, Shwerief Hussen

Zliten University Hospital, Zliten, Lybia

aliyahyaz60@hotmail.com

Hepatic hydatid disease is very common in Libya. In Zliten hospital, we operated 400 patients with hepatic hydatid cysts over period of 20 years. All patients were symptomatic. Their ages varied from 3 to 85 years including 215 female and 185male patients. Their symptoms varied from abdominal pain to abdominal mass. 67 patients were admitted through Accident and Emergency Department with acute presentations including fever, skin rash, jaundice and shock with acute abdominal pain.

Those 67 patients had necessary investigations, resuscitation and underwent emergency surgery. The hepatic cysts in all patients were excised, and the obstructive jaundice was cleared in those patients with obstructive jaundice. Unfortunately, one of the patients died two days after the surgery because of multiple organ failure (MOF) Morbidity was wound Infection, bile leak and recurrence rate, all reported in our series.

##### P-67. Bulgarian experience in the chemotherapy of human liver cystic echinococcosis

Vutova Kamenna, Todorov Todor

Department of Infectious Diseases, Parasitology and Tropical Medicine, Specialized Hospital of Infectious and Parasitic Diseases “Prof. J. Kirov”, Medical University, Sofia, Bulgaria

K_Vutova@abv.bg

**Background:** Echinococcosis is a chronic disease in humans. The most frequently used method of treatment in patients with hydatid disease is surgical, but 33 years ago have started the first two years multicentre clinical trials in the world in five centers (Beirut, Paris, Roma, Sofia and Zurich) on the treatment of human echinococcosis with benzimidazole carbamates, coordinated by the WHO. We reviewed all cases treated during this period.

**Methods and Results:** During the period 1979–2013, two hundred fifty patients with liver echinococcosis are treated with albendazole (10 mg/kg/day for four courses of 30 days) and follow up. Forty four of the patients received mebendazole (30–50 mg/kg/day for 3–6 moths). The treatment response based on objective criteria, provided by imaging methods – Ultrasound, Computed tomography and MRI, confirmed by serological methods (ELISA, indirect hemagglutination, immunofluorescent test, latex agglutination). The cyst changes which characterized the cyst responses is a criterion for determining the final chemotherapeutic effectiveness in individual patient. Following chemotherapy, the initial change in liver cysts we observed endocysts ruptured (detachment) at the end of 1st or 2nd to 3th month therapy. The bigger hydatid cysts (<5 cm) at first increased in size, endocyst detached in 3–4-th months, deformed and after 5-th month start to decreased in size. The subsequent changes (hyperechoic/hyperdense appearance, size reduction), detected in all cysts in following albendazole therapy and had to be considered as damage of hydatid cysts. There were no serious side effects related to the applied drugs. The results of chemotherapy showed positive response of hydatid cysts extinction observed in our patients, with multiple, recurrent, multiorgan echinococcosis and with single cysts.

**Conclusion:** Chemotherapy is an alternative for patients with echinococcosis and the compelling facts are reason to be accepted as a successful method of treatment.

##### P-68. On conservative treatment of human hydatidosis as a combination of albendazole and an immunomodulator (isoprinosin, Respivax^®^) and clinical follow-up

Vuchev Dimitar, Popova-Daskalova Galya

Chair of Infectious diseases, Parasitology and Tropical medicine, Medical University, Plovdiv, Bulgaria

dvutchev@yahoo.com

**Background:** In Bulgaria the morbidity rate of hydatid disease showed an increase over the last two decades. The most important clinical aspects are an adequate treatment and follow-up after making an exact diagnosis. Due to introduction of benzimidazole carbamates as a conservative treatment the therapeutic options have expanded. However, immunosuppression also presents in humans with hydatidosis. Combined treatment with an anthelmintic drug and an immunomodulator was used in patients with liver or lung hydatidosis to achieve better clinical effects.

**Methods:** The immunomodulators (Isoprinosin in liver hydatidosis, Respivax in lung hydatidosis) was administered with an anthelmintic drug – albendazole to three different groups of patients with hydatid disease: (1) after surgery – to prevent recurrence of liver and lung hydatidosis; (2) accompanying PAIR – to prevent recurrence of liver hydatidosis; (3) as a conservative treatment of inoperable cases, recurrent disease, multiple organ involvement, cyst rupture and if patients refuse surgery. After treatment two hundred patients had follow-up using imaging (US of the liver and X-rays of the lungs) and serological testing for a period of 2–10 years.

**Results:** No serious side effects of immunomodulators were noted. No recurrences of the disease were recorded during the follow-up. Clinical symptoms (abdominal discomfort in liver hydatidosis, sputum and haemoptysis in lung hydatidosis) resolved quickly. Most of the patients with pulmonary echinococcosis eliminated the cyst content and the residual membranes through the sputum, but in some patients cyst degeneration was noted on X-ray. In all patients with liver hydatidosis degeneration sings in the cysts were noted, including – significant deformation, size reduction and disappearance of hydatid cysts.

**Conclusion:** On the basis of the accumulated data it might be concluded that combination of albendazole and an immunomodulator (Isoprinosin, Respivax) as treatment in echinococcosis leads to more rapid and better results – on an average within 2–3 months for pulmonary hydatidosis and 6 months for liver ones.

##### P-69. Hydatid embolisms of pulmonary artery

Achour Karima^1^, Laribi Abdesslam^1^, Nekhla Ahmed^1^, Dehal Siham^1^, Ghebouli Noureddine^1^, Riquet Marc^2^, Ameur Soltane^1^

^1^Mustapha University Hospital, Algiers, Algeria

^2^Georges Pompidou European University Hospital, Paris, France

achour.karima@gmail.com

**Background:** Hydatid disease is a frequent pathology in the Maghreb countries (Morocco, Algeria, Tunis). It is a benign disease which may have very aggressive behaviour and course. Normally it becomes symptomatic when there is a complication like intra bronchial rupture or infection. Clinical presentation is variable depending on location and needs different management. We present a very rare case of multiple unilateral hydatid emboli occurred in right pulmonary artery of a patient with history of operated renal hydatid cyst.

**Observation:** A 31 years-old male patient presented with right sided flank pain. Sonography, MRI and Pet-scan showed a ruptured hydatid cyst in secretory system of a unique right kidney. It made compressive effect causing hydronephrosis. Resection in Urology Department was followed by infection necessitating two other surgical interventions. Six months later simple chest radiography revealed a marked augmentation of right pulmonary artery size. Right sided massive pulmonary artery embole of hydatid vesicles was demonstrated by thoracic angio-scanner. Abdominal CT-scan showed a calcified mass which partially obstructed the right renal pelvis and communicated with the renal vein. Right pneumonectomy was performed. Post operative course was complicated by a cavitary empyema due to Pseudomonas aeroginosa and Achromobacter xylosoxidans, managed successfully by double drainage and frequent lavage. The patient was discharged on 43rd postoperative day.

**Discussion/Conclusion**: to our knowledge only one similar case of this rare exceptional entity requiring a pneumonectomy has been observed. The supposed explication is rupture of the cyst into the renal vein but the right unilaterality of disease in both cases is questioning and remains a mystery to us.

##### P-70. Analysis of patients with echinococcosis hospitalized in university centre for maritime and tropical medicine in Gdynia, Poland in 2003–2013*

Sulima Małgorzata^1^, Nahorski Wacław^2^, Kuna Anna^1^, Felczak-Korzybska Iwona^1^, Wołyniec Wojciech^1^, Szostakowska Beata^3^, Lass Anna^3^

^1^University Centre for Maritime and Tropical Medicine, Gdańsk, Poland

^2^Departament of Tropical Medicine, Gdańsk, Poland

^3^Chair of Tropical Medicine and Parasitology, Medical University of Gdańsk, Gdańsk, Poland

m.sulima@poczta.fm

**Background:** Echinococcosis is not considered as an important disease impacting on Polish public health care. However, recently a number of new cases of both types, alveolar and cystic echinococcosis has been increasing in Poland. It is probably due to increasing awareness of this disease among clinicians and patients as well as easier access to diagnostic methods, especially to ultrasonographic examination. In the case of alveolar echinococcosis an increasing population of infected foxes in Poland also plays a vital role.

**Methods:** University Centre for Maritime and Tropical Medicine in Gdynia has been involved in diagnosis and treatment of patients with echinococcosis for many years. Within the last 10 years almost 100 patients were hospitalized in our Clinic to carry out the follow-up examinations and determine further proceedings, including surgery like PAIR, excision of parasitic lesion or liver transplantation. The aim of the work was to analyze the clinical material related to this disease. We present some statistical information on the number of patients admitted to our Clinic with suspected alveolar or cystic echinococcosis, considering diagnostic methods (imaging techniques, serological tests, histopathology) and medical therapy that was instituted.

**Results:** Clinical experience indicates that diagnosis of echinococcosis is sometimes very difficult, moreover treatment is often complicated, especially regarding patients not assigned to radical surgery or after liver transplantation. In inoperable cases pharmacological treatment combined with supportive therapy including decompressing of mechanical jaundice, percutaneous drainage of necrotic lesions in the liver or surgical treatment of hydronephrosis or bowel obstruction, gave a chance for survival and improved the quality of patients’ lives.

**Conclusion:** Echinococcosis still remains a dangerous disease. Increasing number of new echinococcosis cases detected over recent years indicates the need of further action to improve early diagnosis and treatment of this condition.

*This study was partially supported by the Research Grant No. NN402 587140 from the State Committee for Scientific Research, Poland.

